# Embers of autoregression show how large language models are shaped by the problem they are trained to solve

**DOI:** 10.1073/pnas.2322420121

**Published:** 2024-10-04

**Authors:** R. Thomas McCoy, Shunyu Yao, Dan Friedman, Mathew D. Hardy, Thomas L. Griffiths

**Affiliations:** ^a^Department of Computer Science, Princeton University, Princeton, NJ 08542; ^b^Department of Psychology, Princeton University, Princeton, NJ 08542

**Keywords:** cognitive science, artificial intelligence, large language models

## Abstract

The widespread adoption of large language models (LLMs) makes it important to recognize their strengths and limitations. We argue that to develop a holistic understanding of these systems, we must consider the problem that they were trained to solve: next-word prediction over Internet text. By recognizing the pressures that this task exerts, we can make predictions about the strategies that LLMs will adopt, allowing us to reason about when they will succeed or fail. Using this approach—which we call the teleological approach—we identify three factors that we hypothesize will influence LLM accuracy: the probability of the task to be performed, the probability of the target output, and the probability of the provided input. To test our predictions, we evaluate five LLMs (GPT-3.5, GPT-4, Claude 3, Llama 3, and Gemini 1.0) on 11 tasks, and we find robust evidence that LLMs are influenced by probability in the hypothesized ways. Many of the experiments reveal surprising failure modes. For instance, GPT-4’s accuracy at decoding a simple cipher is 51% when the output is a high-probability sentence but only 13% when it is low-probability, even though this task is a deterministic one for which probability should not matter. These results show that AI practitioners should be careful about using LLMs in low-probability situations. More broadly, we conclude that we should not evaluate LLMs as if they are humans but should instead treat them as a distinct type of system—one that has been shaped by its own particular set of pressures.

## Significance

ChatGPT and other large language models (LLMs) have attained unprecedented performance in AI. These systems are likely to influence a diverse range of fields, such as education, intellectual property law, and cognitive science, but they remain poorly understood. Here, we draw upon ideas in cognitive science to show that one productive way to understand these systems is by analyzing the goal that they were trained to accomplish. This perspective reveals some surprising limitations of LLMs, including difficulty on seemingly simple tasks such as counting words or reversing a list. Our empirical results have practical implications for when language models can safely be used, and the approach that we introduce provides a broadly useful perspective for reasoning about AI.

Large language models (LLMs), such as ChatGPT (1), Claude (2), and Llama (3), receive a piece of text as input and generate additional text as output. Virtually any task can be framed in the form of linguistic queries, so LLMs could be applied to virtually any task—from summarizing text to generating computer code. This flexibility is exciting: it led one recent paper to argue that LLMs display “sparks of artificial general intelligence” (4). However, it also hinders us from understanding LLMs holistically. Since we can only run a finite number of tests, how can we understand a system whose potential scope is infinite? Answering this question requires some method for deciding which tests will be most informative about the general strengths and weaknesses of LLMs.

**Fig. 1.**
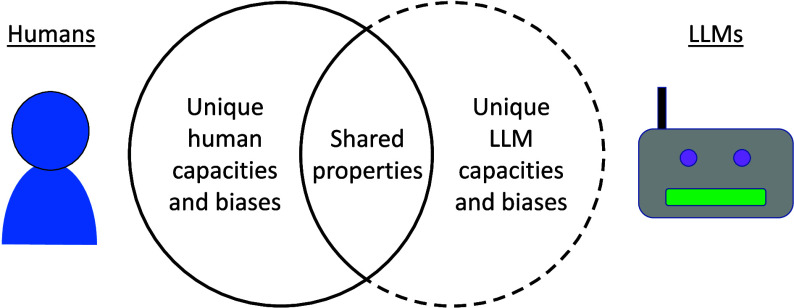
Humans and LLMs have some shared properties and some properties that differ. If we analyze LLMs using tests designed for humans, we risk identifying only the shared properties, missing the properties that are unique to LLMs (the dotted region of the diagram). We argue that to identify the properties in the dotted region, we must approach LLMs on their own terms by considering the problem that they were trained to solve: next-word prediction over Internet text.

One popular way to select evaluations is to use a human-centric approach: test for the properties that are viewed as most important for characterizing human cognition. For instance, GPT-4 was evaluated on the SAT and other real-world exams (1), and many LLMs have been evaluated on tests from cognitive psychology (5), such as tests of analogical reasoning (6). Other analyses have compared the internal representations of LLMs to representations from linguistics (7) and neuroscience (8). Human-inspired analyses are valuable because they allow AI to benefit from the wealth of nuanced tests that have been developed in cognitive science. However, human-centric approaches also have an important limitation: LLMs are not humans, so the tests that might be most informative about them may differ from the tests that are most informative about humans (9). In particular, a human-centric approach runs the risk of highlighting the strengths of these models—their overlap with human abilities—without revealing their idiosyncratic weaknesses (Fig. 1). How can we approach the problem of understanding a new type of intelligence, evaluating it on its own terms?

We argue for another approach that has been very productive in cognitive science: understanding intelligent systems by understanding the sorts of problems that they developed to solve (10–13). This approach—which we refer to as the teleological approach—is complementary to the perspectives discussed above because it focuses on the system’s goals and environment, rather than its representations and processing mechanisms. The crucial question to ask, then, is what problem(s) do LLMs need to solve, and how do these pressures influence them? We focus on perhaps the most salient pressure that defines any machine learning system, namely the task that it was trained to perform. For the LLMs that have been the focus of recent attention in AI, this task is autoregression—next-word prediction (14, 15)—performed over Internet text. (Many LLMs are further optimized for additional objectives, but we focus on next-word prediction because it comprises the bulk of training; see below for discussion.) We argue that a full understanding of LLMs should be strongly informed by the fact that next-word prediction is their primary training objective. We therefore hypothesize that, even when they are asked to perform tasks that seem very different from next-word prediction—e.g., solving math problems—their performance on such tasks is highly influenced by the autoregressive substrate from which all of their abilities emerge.

**Fig. 2.**
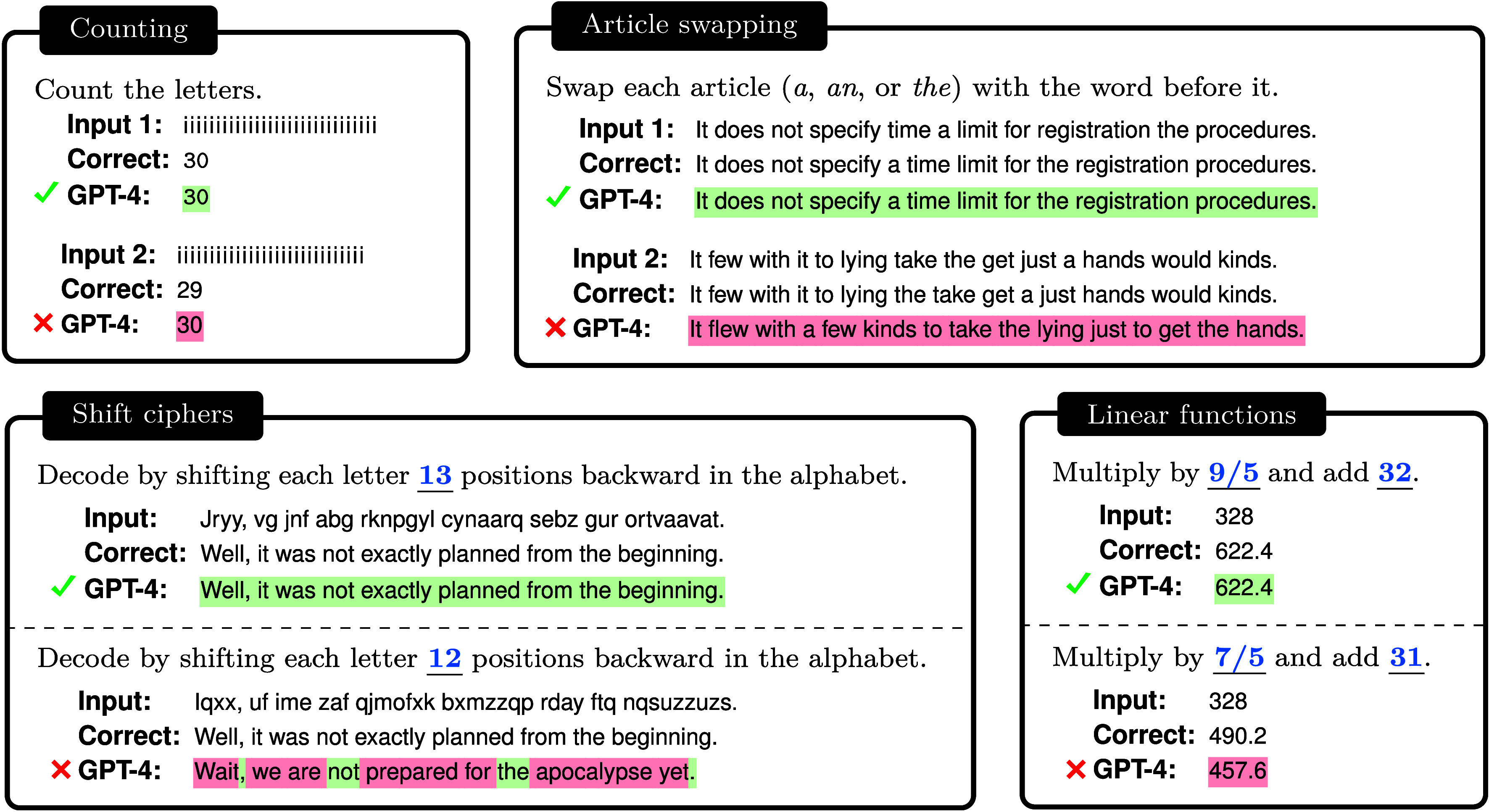
GPT-4 struggles on some seemingly simple tasks such as counting, article swapping, shift ciphers, and linear functions. In the counting and article swapping examples, GPT-4 fails in the cases where the correct output is a low-probability piece of text (for the counting example, we refer to 29 as low-probability because it occurs much less frequently in natural corpora than 30 does). In the shift cipher and linear function examples, GPT-4 performs well on the task variants that are common in Internet text but poorly on the variants that are rare (note that the shift cipher with a shift of 13 is over 100 times more common in Internet text than the shift cipher with a shift of 12; and the linear function f(x)=(9/5)x+32 is common because it is the Celsius-to-Fahrenheit conversion, while the other linear function has no special significance). The GPT-4 predictions were obtained using gpt-4-0613 on the OpenAI API; other model versions (e.g., the online chat interface) may give different predictions.

A mismatch between the problem that a system developed to solve and the task that it is given can have significant consequences. Just as the human drive to obtain sweet and fatty foods can be maladaptive in a world where those foods are easily available, the autoregressive tendencies of LLMs can cause problems when they are given a task that is not next-word prediction. In this paper, we exhibit what might seem like surprising failures of LLMs on tasks that are straightforward for humans to perform, such as using simple ciphers, constructing acronyms, calculating linear functions, and counting; see Fig. 2. These failures can be understood in terms of a conflict between next-word prediction and the task at hand. Being able to reason about when LLMs will fail at a task is critical as these systems become more widely deployed.

Based on an analysis of the problem that LLMs are trained to solve (statistical next-word prediction), we make three predictions about how LLMs will be influenced by their origin in this task—the embers of autoregression that appear in these systems even as they might show sparks of artificial general intelligence. These predictions are listed in Table 1. For example, we predict that, even when the task is a deterministic one that does not depend on probability, LLMs will achieve higher accuracy when the answer is high-probability than when it is low-probability. In the next section, we discuss these hypotheses in more detail.

**Table 1.** Effects on the performance of language models that are attributable to the fact that they are statistical next-word prediction systems

Property	Description	Example
**Sensitivity to output probability**	Even when the task is deterministic, LLMs achieve higher accuracy when the correct answer is high-probability text than when it is low-probability text.	When asked to reverse a sequence of words, GPT-4 gets 97% accuracy when the answer is a high-probability sentence yet 53% accuracy when the output is low probability.
**Sensitivity to input probability**	Even when the task is deterministic, LLMs sometimes achieve higher accuracy when the input text is high-probability than when it is low-probability, but input probability is less influential than output probability.	When asked to encode sentences in a simple cipher (rot-13), GPT-4 gets 21% accuracy when the input is a high-probability sentence yet 11% accuracy when the input is low-probability.
**Sensitivity to task frequency**	Even when there is no difference in the complexity of the tasks, LLMs perform better on tasks that are frequent than ones that are rare.	When asked to translate English sentences into Pig Latin, GPT-4 gets 42% accuracy when using the most common variant of Pig Latin but only 23% accuracy when using a rare variant.

We then test these hypotheses through an extensive set of experiments. Since our goal is to identify sources of difficulty for LLMs, our experiments need to take place in settings where LLMs make at least some errors. To create such settings, we use an adversarial strategy that follows straightforwardly from our hypotheses: we use tasks that push LLMs into low-probability situations where we expect they will perform poorly. For instance, one task we use is decoding simple ciphers, since text written in a cipher is a low-probability subspace of the broader space of possible types of text. Table 2 gives the full list of tasks that we use. As long as models indeed have a nonnegligible error rate on these tasks, we can investigate which factors cause this error rate to increase or decrease—focusing on the factors that we have hypothesized will have an effect.

**Table 2.** The tasks that we used to evaluate LLMs

Task	Description	Example
**Article swapping**	Swap each article (a, an, or the) with the preceding word.	In box the I saw key a. → In the box I saw a key.
**Reversal**	Reverse a sequence of words.	everyone! morning, Good → Good morning, everyone!
**Counting**	Count the words or letters in a list.	lively news exhibit steep → 4
**Acronyms**	Join the first letters of the words in a list.	view inch show into tray → VISIT
**Linear function**	Apply the function f(x)=(9/5)x+32.	328 → 622.4
**Multiplication**	Multiply two three-digit numbers.	351 times 373 → 130923
**Sorting**	Sort a list of words in alphabetical order.	into, trek, game, magic → game, into, magic, trek
**Keyboard cipher**	Replace each letter with the one to the right of it on a keyboard.	Hello world! → Jraap eptaf!
**Shift cipher**	Decode by shifting each letter 13 positions backward in the alphabet.	Fgnl urer! → Stay here!
**Pig Latin**	Move the first consonant cluster of each word to the end and add −ay.	frogs aren’t noisy. → ogsfray aren’tay oisynay.
**Birthdays**	Return the birth date of a provided public figure.	Jeremy Lin → August 23, 1988

To keep this table manageably sized, some of the examples are not from the datasets that we used to evaluate LLMs but are instead shorter examples of the tasks that those datasets target.

Using this strategy, we find robust evidence for the effects described in Table 1: as task probability or example probability varies, LLM accuracy can indeed vary substantially in the ways that we have hypothesized. Overall, our findings illustrate how we can understand LLMs more clearly if we recognize the pressures that have shaped them.

## Background: LLMs

LLMs are trained to take in the start of a piece of text and then predict what word will come next.^*^ The LLM’s prediction takes the form of a probability distribution, specifying for each word in the vocabulary the LLM’s predicted probability that this word will be the next one. For example, given the input *I wrote a __*, an LLM should assign a high probability to *letter* or *book* but a low probability to *waffle* or *the*. In almost all contexts, there are multiple possibilities for what could appear next, so this task is probabilistic rather than deterministic.

The way that an LLM comes to perform next-word prediction is by learning from data. The LLM is defined by a large number of numerical parameters, which govern how it maps an input to a predicted probability distribution. These parameters are initialized randomly and are updated based on data: The LLM is shown many passages of text, and for each position in each passage, it predicts what words are likely to appear in that position. The LLM is then shown the word that actually did appear, and its parameters are adjusted such that, if it were shown the same input again, it would assign a higher probability to the correct word than it previously did. As the training proceeds, the LLM’s text-predicting abilities become stronger. A trained LLM can be used to generate text (such as a chatbot response) by giving it an input and then sampling a word from the probability distribution that it generates. To produce text longer than one word, the process is iterated, using the LLM’s previously produced word(s) as part of the input when predicting each successive word.

LLM training data are typically scraped from the Internet, resulting in a diverse range of types of text. A powerful consequence of this diversity is that learning to perform next-word prediction gives the LLM experience with a wide range of tasks, including many that might seem very different from next-word prediction (15). For example, an LLM can learn to summarize through experience with predicting what comes after *In summary*, and it can learn arithmetic via inputs like *17 + 34 = __*. In this way, a single LLM can learn to perform many tasks that historically would have required multiple specialized systems. Therefore, next-word prediction is a major factor contributing to the power of LLMs—but, as we will see below, it also leads to some important shortcomings.

## A Teleological Approach to Understanding LLMs

To understand an information-processing system such as an LLM, the approach we argue for is to characterize the problem that the system solves and to then use this characterization as a source of hypotheses about the system’s capacities and biases. We refer to this as the teleological approach because it focuses on the system’s goal (*telos* in Greek) (e.g., ref. 16). Teleological explanation is a common strategy in making sense of biological systems, manifest in computational-level (10) and rational (12) analysis in cognitive science and adaptationist explanations in evolutionary biology (17, 18) and evolutionary psychology (12). Just as teleological explanations can be incomplete or misleading in these settings (19, 20), we do not anticipate that all properties of LLMs can be understood via their goals. However, we believe that this is a useful lens through which to study these systems and generate predictions about their behavior. In the rest of this section, we describe more clearly the goals that LLMs are trained to accomplish. We then use this analysis to generate hypotheses about LLM behavior.

### What Problem Do LLMs Solve?.

Anderson (12) argued that the problem solved by a cognitive system can be characterized by three factors: the system’s goal, the environment in which the system pursues this goal, and the computational limitations that constrain the system. For a machine learning system, those factors correspond to the following aspects of a model:

1. Training task: What goal is the system trained to accomplish?
2. Training distribution: What types of examples is the system trained on?
3. Model architecture: What computational tools does the system have access to?

For standard LLMs, the training task is next-word prediction, the training distribution is a distribution over Internet text, and the model architecture is a neural network (specifically, a Transformer: 21). Thus, the problem that an LLM must solve is the following: How to perform next-word prediction on samples of Internet text, given the mechanisms available in a neural network.

Note that many recent LLMs are not solely trained on next-word prediction but also go through a training phase based on instruction tuning (22), which aims to align model behavior with human preferences. In this paper, we only analyze next-word prediction, leaving instruction tuning for future work. We start with next-word prediction because, during LLM training, the next-word prediction phase is much longer than the instruction tuning phase. We therefore conjecture that next-word prediction plays a greater role in shaping LLMs than instruction tuning does, making next-word prediction a reasonable initial focus for the goal of understanding LLMs. Note also that some LLMs (e.g., some recent versions of GPT-4) are augmented with components that go beyond pure text, such as image-processing abilities or code-execution modules. In this paper, we only consider LLMs that process text sequences, though the teleological perspective could be applied to augmented LLMs in future work; see the Discussion.

Importantly, the tasks for which LLMs are used often differ from the problem they were trained to solve. For example, even though they were trained for next-word prediction, LLMs are sometimes asked to develop Python code. Why does this discrepancy matter? When a system is adapted for one purpose but co-opted for a different one, the original purpose may influence the system’s nature in ways that would not make sense if only the new purpose were considered. As an example from biology, the basic mammalian body plan evolved for a life on four legs, but humans recently evolved to instead walk on two legs. Biologists have argued that the friction between our quadrupedal roots and our bipedal lifestyle contributes to several musculoskeletal ailments that are common in humans (23). For instance, having our spines be perpendicular to the ground rather than parallel with it causes strains that make humans susceptible to lower back pain (24). We anticipate that the goals, training distribution, and model architecture used in LLMs create opportunities for such mismatches.

### Hypothesized Embers of Autoregression.

Do LLMs face anything analogous to humans’ back pain—any quirks attributable to a mismatch between what they “adapted” to do and what they are used for? To answer this question, we must consider the types of solutions that would arise from the pressures that shape LLMs. First, the fact that LLMs are neural networks makes them statistical systems. The other two factors—the task of next-word prediction and the training distribution of Internet text—dictate which statistics they are sensitive to: the statistics of word sequences in Internet text. Building on this analysis, we make three predictions (listed in Table 1) about ways in which LLM performance is influenced by frequency and probability. A crucial aspect of these predictions is the first clause of each one. For instance, it is clear that probability is important for next-word prediction, but we are predicting that probability will influence LLMs even in tasks that are not inherently probabilistic, such as reversing a list. In later sections, we develop these hypotheses in more detail and provide extensive evidence supporting them.

### What We Are Not Arguing.

We are arguing that certain types of tasks and examples will be harder for LLMs than others. We do not claim that LLMs are incapable of handling these tasks and examples. That is, our predictions are of the form “for LLMs, X is harder than Y” rather than “LLMs can’t do X.”

This distinction is important for explaining how we set up our experiments. For most experiments, we test LLMs using basic prompting (i.e., simply asking them to provide an answer to a query), even though LLM accuracy can be substantially increased via more sophisticated inference techniques such as chain-of-thought prompting (25, 26) or the tree-of-thoughts framework (27). Our decision to use basic prompting would be problematic if we were claiming that LLMs cannot perform our tasks: such claims would be true only if LLMs fail under all prompting approaches, so investigations of such claims should use the strongest possible prompting approach. However, since this is not the type of claim we are making, it is not necessary to use the strongest available prompting paradigms. Instead, because our claims are comparative (“for LLMs, X is harder than Y”), what is important is ensuring that the two conditions (X and Y) are evaluated in the same way as each other. Since any prompting approach could work for this purpose, we chose the approach that is the fastest and most straightforward to run, namely basic prompting—though see *Discussion* for experiments with other prompting techniques.

In addition, we are not claiming that researchers have ignored the autoregressive origins of LLMs. Indeed, in the “Sparks of artificial general intelligence” paper (4), Section 8 is titled “Limitations of autoregressive architecture highlighted by GPT-4,” and it considers failures of planning in arithmetic and text generation that result from only predicting the next word in a sequence. What we are claiming is that this aspect of LLMs has been neglected in constructing effective evaluations of their capacities. As highlighted in Fig. 1, much of the literature evaluating LLMs has started with tasks that are viewed as important indicators of human abilities and then assessed how well LLMs can do them. By instead starting with tasks that we anticipate will be challenging for systems that are focused on next-word prediction, we get a more balanced view of what kinds of tasks are easy or hard for these systems.

## Motivating Our Predictions: A Bayesian Analysis

In Table 1, we listed several hypotheses about factors that cause difficulty for LLMs. Here, we show how we arrived at these hypotheses by analyzing the problem that LLMs need to solve. To make this discussion more concrete, we will provide brief examples from one particular domain, namely shift ciphers (defined below). Later in the paper, we will further test our predictions with an additional ten tasks.

### Running Example: Shift Ciphers.

In a shift cipher, a message is encoded by shifting each letter forward in the alphabet a certain number of positions. For example, with a shift of 1, *How are you?* becomes *Ipx bsf zpv?* because the letter after *h* is *i*, the letter after *o* is *p*, etc. One particularly prominent case is rot-13 (short for “rotate by 13”), the cipher with a shift of 13 positions. Rot-13 is popular online as a spoiler-free way to share information. For example, in some puzzle-solving forums, members write hints in rot-13 so that those who want to solve the puzzle without help will not read the hint inadvertently.

### A Bayesian Analysis.

LLMs are statistical systems. We therefore expect that their predictions will be influenced by probability, even in deterministic situations where probability should be irrelevant. To motivate this hypothesis more formally, we first frame the LLM’s task as finding the most probable output given some input, where the input is the start of a word sequence, and the output is the sequence’s continuation. In other words, the LLM should find the output that maximizes P(output|input) (the probability of the output given the input). By Bayes’ rule, this problem is equivalent to maximizing P(input|output)P(output). Therefore, as long as there are multiple outputs for which P(input|output) is nonzero, the LLM’s predictions will be influenced by the unconditional probability of the output, P(output): among the candidates that yield a nonzero value for P(input|output), LLMs will be biased toward selecting ones with a high P(output).

The argument made so far only covers nondeterministic situations, when there are multiple outputs that yield a nonzero P(input|output). Now consider deterministic settings, where there is only one output that could go with a given input; rot-13 is one such setting. In principle, P(output) should not matter in such cases because the likelihood, P(input|output), fully determines the output on its own: this value is nonzero for the correct output and zero for all others. In practice, however, an LLM’s likelihood estimates are unlikely to be perfect, meaning that there will in fact be multiple candidate outputs that yield a nonzero likelihood. With multiple output candidates in consideration, P(output) will affect the model’s predictions in practice, even if in theory it should be irrelevant. This analysis points toward several hypotheses about LLM behavior, which we discuss in the next few paragraphs.

### Sensitivity to Output Probability.

**Fig. 3.**
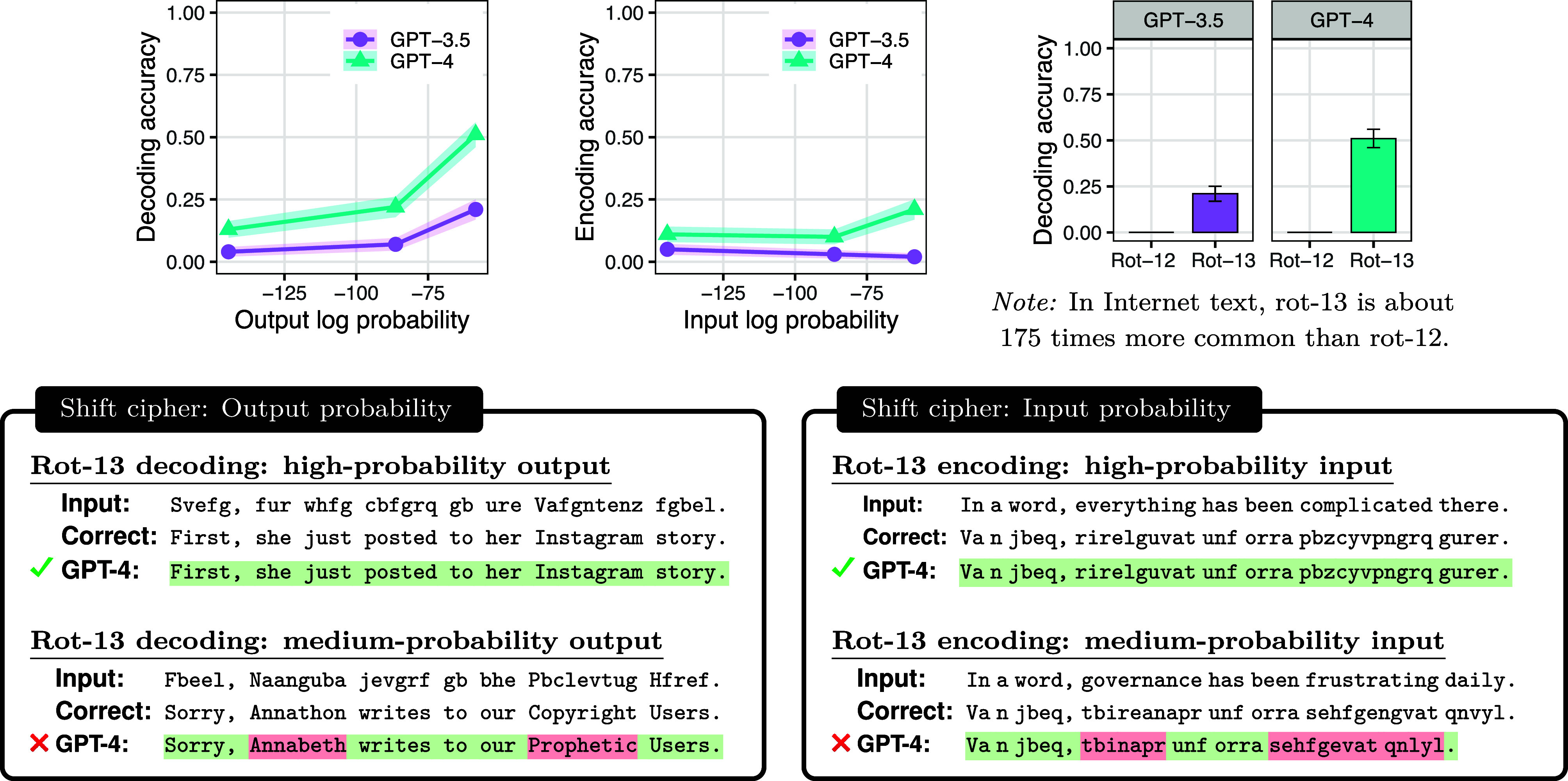
When processing shift ciphers, LLMs are highly sensitive to probability, even though shift ciphers are deterministic. *Top Left*: Effect of output probability. When decoding text written in the rot-13 cipher, both models score better when the answer is high-probability than when it is low-probability. *Top Middle*: Effect of input probability. As predicted, input probability does not show as strong an effect as output probability. *Top Right*: Effect of task probability. Both models score better on a frequently occurring shift cipher (rot-13) than on a rare one (rot-12). All error intervals show one SE. *Bottom*: Examples for output and input probability; see Fig. 2 for task probability examples.

The most straightforward consequence of our analysis is the prediction that LLMs will be biased toward producing outputs that are high-probability word sequences, meaning that they will perform better when the correct output is indeed high-probability than when it is not. The results in Fig. 3 (*Top Left*) support this prediction: for both GPT-3.5 and GPT-4, accuracy increased as the log probability of the correct output was increased, supporting the hypothesis that these systems are influenced by the unconditional probability of potential outputs.

### Sensitivity to Input Probability and Task Probability.

Under our analysis, the factor that causes LLMs to favor high-probability outputs is noise in the LLM’s estimate of the likelihood. We therefore hypothesize that LLMs will be particularly error-prone in situations where their likelihood estimates are particularly noisy. We identify two such situations: when task probability is low and when input probability is low.

Task probability is the probability that a task will be illustrated in a random sample of text. This probability determines how many examples of the task will appear during an LLM’s training. When the task probability is low, the LLM’s likelihood estimates will be noisy because it will not have had enough experience with the task to produce precise estimates of task-relevant statistics, leading to poorer overall performance.

This prediction is borne out for shift ciphers. As mentioned above, rot-13 is a commonly used shift cipher. In contrast, rot-12—which uses a shift of 12 rather than 13—is no more complex than rot-13 but is used rarely. We found that GPT-3.5 and GPT-4 performed much better at decoding messages written in rot-13 than rot-12 (Fig. 3, *Top Right*), supporting the conclusion that LLMs are sensitive to task probability.

Another factor that we expect will reduce the quality of likelihood estimates is the probability of individual examples: a trained LLM will have had less experience with low-probability strings than high-probability ones. Therefore, the information that the model captures may be less robust for low-probability strings than high-probability strings. Information learned during pretraining is important for both processing the input and producing the output. We have already hypothesized that LLMs will be sensitive to output probability; this argument adds the additional hypothesis that they will be sensitive to input probability. Fig. 3 (*Top Middle*) shows that GPT-4 achieves higher accuracy when the input is high-probability than when it is low-probability, illustrating that LLMs can indeed be sensitive to input probability. GPT-3.5 shows no significant effect of input probability. In the next subsection, we describe why LLMs may show less sensitivity to input probability than output probability or task probability.

We have identified these effects as consequences of autoregression, but autoregression is not the only objective that we would expect to produce these effects. Sensitivity to word sequence probability would likely arise in any system that models text distributions, and sensitivity to task probability would likely arise in any system that uses statistical learning. We focus on autoregression because it is central to current LLMs, but future work could use the teleological perspective to investigate whether other objectives also yield these effects.

### Strength of Hypothesized Effects.

We have hypothesized that LLMs will be sensitive to the probability of both the input and the target output. Though these effects sound similar, we have predicted them for two different reasons. Input probability was predicted to matter only when the task depends on information about the input that models mainly learn through experience with that specific input (i.e., when the model’s ability to estimate the likelihood, P(input|output), does not generalize to novel inputs). We expect that such situations only arise occasionally, such that a model’s dependence on input probability may not manifest itself routinely. In contrast, output probability was predicted to matter not only in the same situations where input probability matters but also in an additional context: whenever the model has any uncertainty about what the output is (in which case the model will use the prior probability of the output to help resolve its uncertainty). We expect that this situation is common since neural networks rarely produce probabilities that are precisely equal to one. We therefore expect that output probability will be more broadly influential than input probability.

Another relationship between our hypotheses is that task probability and input probability were hypothesized to matter for similar reasons: both influence the training data in ways that will affect the level of noise in the LLM’s learned likelihood estimates. However, we predict that task probability will lead to such effects more reliably than input probability: we expect that LLMs will often be able to generalize to novel inputs (because neural networks usually perform well on new examples that are similar to ones they have seen), such that low input probability will not always hamper LLM performance. In contrast, novel tasks require a higher-order type of generalization that is more challenging for neural networks, so we predict that task probability will routinely have an effect.

In sum, we have predicted that output probability and task probability will be more broadly influential than input probability. An initial piece of evidence for this hypothesis is the fact that, in Fig. 3, output probability and task probability have a substantial effect, whereas input probability shows only a minor effect and only in one model.

## Overview of Experiments

In the next several sections, we test the hypotheses developed above by analyzing LLM performance on a wider range of tasks. We evaluated five models: GPT-4 (1), GPT-3.5 (1), Claude 3 (2), Llama 3 (3), and Gemini 1.0 (28). GPT-4 is the state-of-the-art in many areas and is the focus of the “sparks of AGI” paper (4) that our work connects to. GPT-3.5 is similar to GPT-4 but smaller, enabling us to investigate the effects of model scale. The remaining models are other prominent LLMs. We accessed all models through APIs and used a temperature of 0.0; see *Materials and Methods* for more details. We also ran preliminary tests with the smaller-scale models OLMo (29) and Llama 2 (3), but their scores were too low to yield meaningful trends, with accuracies of 0% on most tasks.

The tasks that we tested these models on are described in Table 2. We chose these tasks for two reasons. First, as described in the introduction, we selected tasks that push models into low-probability situations so that models will have a nonnegligible error rate—a requirement for our goal of observing what causes the error rate to increase or decrease. Second, most of these tasks can be solved with a simple, deterministic algorithm that is invariant to various changes to the task. For example, consider a shift cipher that moves each letter n positions forward in the alphabet. The most straightforward way to decode this cipher is to shift each letter back n positions—an algorithm that succeeds regardless of the value of n and the identity of the words being processed. Thus, if a person demonstrated that they could decipher one message written in rot-13, we might assume that they knew this algorithm and could therefore perform equally well on any other shift cipher or on any other inputs. However, we have hypothesized that LLMs are not invariant to such properties: we expect their performance to vary based on which task variant is used and which inputs are used. The tasks described in the table allow us to test these predictions. Following the “sparks of AGI” paper (4), we do not tune models on these tasks but rather describe the task in the prompt; see *Materials and Methods*.

Many of our tasks involve character-level manipulations of words. This factor might seem unfair to LLMs because they operate over subword tokens (30) rather than characters. However, we gave all models a spelling test (*SI Appendix*, section L) and found that they robustly encode the spelling of their tokens, making it reasonable to test them on tasks that involve character-level manipulations; see also refs. 31 and 32.

## Sensitivity to Output Probability

We have hypothesized that LLMs will perform better when the correct answer is a high-probability string than when it is a low-probability string, even in deterministic situations where the answer could be determined without considering probability. Here, we test this hypothesis by evaluating models on sets of examples that vary in the probability of their outputs (probabilities were estimated using GPT-2 (15); see *SI Appendix*, section J). For example, for shift ciphers, we evaluate accuracy on three sets of examples corresponding to messages that, when deciphered, are high-, medium-, or low-probability sequences of words; see (1) below for examples:

(1)
  a. High-probability: Are they now building a bridge of their own?
  b. Medium-probability: Are dogs yet climbing a jungle of their own?
  c. Low-probability: Are their jungle of dogs a yet climbing own?

**Fig. 4.**
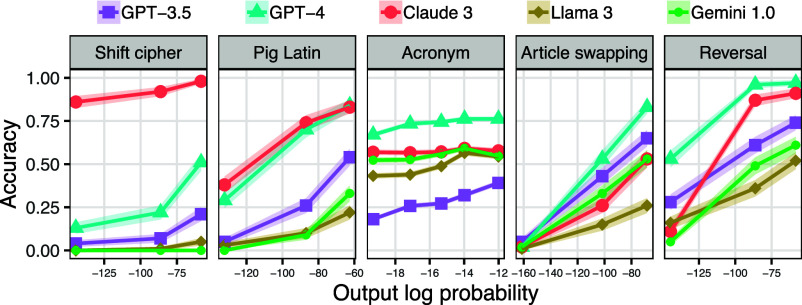
The effect of output probability on model accuracy across five tasks. The intervals around the lines show one SE.

Fig. 4 shows the results. Output probability had a statistically significant effect on accuracy (P<0.05) in all cases except Gemini 1.0 decoding shift ciphers (its accuracy was 0% across conditions) and Claude 3 forming acronyms (P=0.38). Many cases showed large effect sizes; e.g., GPT-4’s article-swapping accuracy ranged from 0.02 on the set of low-probability examples to 0.83 for high-probability examples.

### Analysis of Shift Cipher Errors.

As a more targeted analysis of output probability effects, we gave models messages encoded with a shift of 13 where the correct answer was created by manually changing one word in a high-probability sentence to a new word that was still grammatical but was now low-probability. In such cases, models often “regularized” the output by producing the high-probability sentence that was similar to the correct answer, rather than the correct answer; indeed, GPT-3.5 and GPT-4 produced the regularized version more often than the correct one (see *SI Appendix*, Fig. S23, for quantitative results), as in the following GPT-4 response:

(2)
  a. Correct output: Because of this, their names were chanted for security reasons.
  b. GPT-4 output: Because of this, their names were changed for security reasons.

This tendency to push the output toward a higher-probability sentence further supports the conclusion that LLMs are biased toward high-probability outputs.

### Counting.

**Fig. 5.**
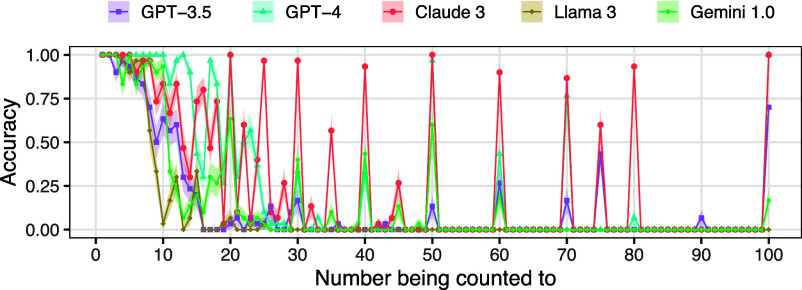
Model performance on counting a list of words, as a function of the length of the list. The intervals around the lines show one SE.

As a final investigation of output probability, we evaluated models on counting how many words are in a list. Fig. 5 plots LLM accuracy as a function of the number being counted to. LLMs often show spikes in accuracy for the numbers that are most frequent in corpora, namely multiples of 10. Further, for all models except Llama 3, accuracy has a higher Spearman correlation with the frequency of the number being counted to than with its magnitude (e.g., the correlation coefficients have an absolute value of 0.84 vs. 0.70 for GPT-4, and 0.85 vs. 0.66 for Claude 3), providing additional evidence for the importance of frequency.

### Summary of Output Probability Effects.

We have shown that the performance of the LLMs we tested is heavily influenced by the probability of the target output, even though the tasks being investigated were not inherently probabilistic. These results support our hypothesis that LLMs are sensitive to the probability of the sequences they must produce.

## Sensitivity to Input Probability

We have hypothesized that LLMs will sometimes perform worse when the input is low probability than when it is high probability. However, we have also hypothesized that the influence of input probability will be less pervasive than the influence of output probability: we predicted that LLMs would use output probability whenever they have some uncertainty about the output—a condition that we expect holds frequently—whereas we predicted that input probability would only matter when an LLM’s ability to process an input is highly dependent on prior experience with that specific input—a condition that we expect to hold less often under the view that, for many tasks, neural networks are not restricted to handling only the inputs they have seen but can also generalize across inputs.

**Fig. 6.**
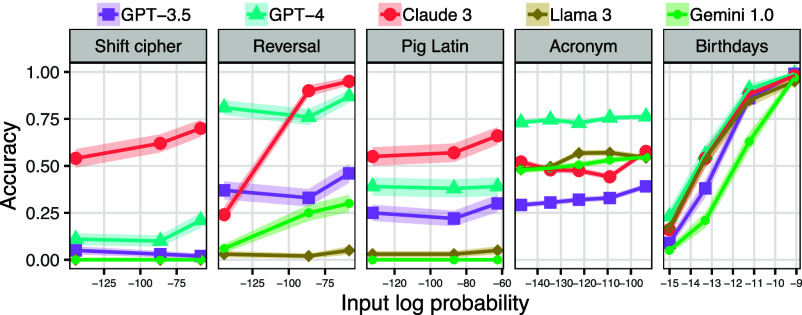
The effect of input probability on model accuracy across five tasks. The intervals around the lines show one SE.

Consistent with these predictions, we found that, for most of the tasks we investigated, input probability had little or no effect on accuracy (Fig. 6); see *SI Appendix*, section E.4 for additional discussion of the asymmetry between input probability and output probability. There was, however, one task—the birthday task—where input probability had a large effect ($P<10^{-15}$ for all models). In this task, a model is given the name of a public figure and is asked to return that person’s birthday. For this task, the answer cannot be deduced from the input alone, so the only way it can be produced is if the model has encountered it during training. This task therefore has the properties that we have hypothesized would lead to input probability sensitivity; the fact that models indeed display this sensitivity therefore supports our analysis.

Additional situations where input probability has a large effect can be found in prior work studying LLM performance on arithmetic (33) and factual recall (34, 35). Overall, we conclude that, under the right conditions, LLM performance can indeed be meaningfully affected by input probability.

## Sensitivity to Task Probability

We have hypothesized that LLMs will perform better on tasks that are frequently illustrated in Internet text than tasks that occur more rarely—even when the rare task is no more complex than the common one. In this section, we test this hypothesis by evaluating models on common and rare variants of several tasks (we use corpus analyses to estimate task frequency; see *SI Appendix*, section K). Fig. 9 summarizes the results.

### Shift Ciphers.

**Fig. 7.**
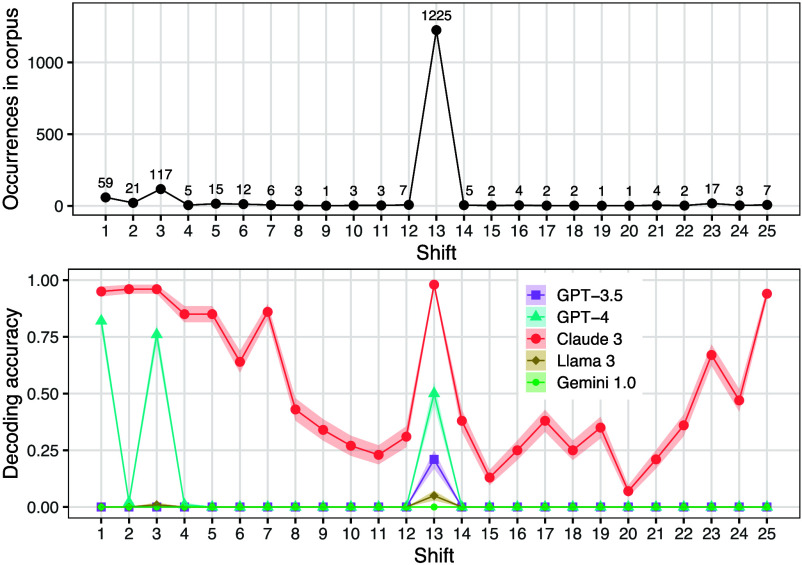
Analysis of shift ciphers with shift levels varying from 1 to 25. *Top*: Number of occurrences of each shift level in the C4 corpus. *Bottom*: Accuracy on decoding shift ciphers with various shifts. Most models display a spike in accuracy at a shift of 13, which the top plot shows to be the most common shift level.

There are 25 possible shift ciphers, corresponding to the 25 unique shifts that can be applied within the alphabet. These different shifts are not all used with the same frequency. Based on an analysis of the C4 corpus (36), we found that the three most common shift levels are rot-1, rot-3, and rot-13 (Fig. 7, *Top*). Rot-1 is common because it is the smallest shift, so it is a natural choice for illustrating the concept of a shift cipher. Rot-3 is common because Julius Caesar famously used this cipher (37), creating a precedent that has influenced many others to adopt a shift of 3 as well (e.g., refs. 38 and 39). Finally, as mentioned above, rot-13 is common because there is a convention of using it in online forums.

If LLMs are indeed sensitive to task probability, we would expect them to perform better on shifts of 1, 3, and 13 than on other shifts. To test this prediction, we evaluated the models on decoding text written with each shift from 1 to 25. The results are striking (Fig. 7, *Bottom*): all models except Gemini 1.0 show a spike in accuracy at 13, which is by far the most common shift. GPT-4 scores 0.50 or above on the three highest-frequency shifts, but its accuracy is less than 0.03 for all other shifts. Claude 3 differs from the other models in that it performs well on some rare shifts, suggesting that it has a more generalizable decoding strategy than other models, but it still also shows a spike in performance at 13. These results therefore follow the prediction that LLMs would perform better on commonly used shifts than rarely used ones.

To test the significance of these task effects, we compared rot-13 to rot-12 (as examples of a common and rare variant, respectively). We only considered GPT-3.5, GPT-4, and Claude 3, as the other models scored near 0% on all shift ciphers. All three of these models had a statistically significantly better performance on decoding rot-13 than rot-12 (P<0.01). We also evaluated these models on encoding; in this case, GPT-4 and Claude 3 still performed significantly better on rot-13 than rot-12 (P<0.01), but for GPT-3.5, there was no significant difference, likely due to floor effects (GPT-3.5’s encoding accuracy was close to 0.0 for both shifts; see Fig. 9).

### Analysis of Shift Cipher Errors.

For shift levels with a low accuracy (e.g., shifts other than 1, 3, or 13 for GPT-4), the incorrect answers that models produce are often recognizable sayings or quotations. For instance, in one rot-10 case, GPT-4 erroneously produced a quote from Shakespeare. Clearly something is rot-10 in the state of Denmark:

(3)
  a. Correct answer: As a doctor of humanities, he was a university professor, founded a university and a newspaper, and won awards in journalism and literature.
  b. GPT-4 output: To be or not to be, that is the question, whether tis nobler in the mind to suffer the slings and arrows of outrageous fortune.

This tendency to produce famous quotations in contexts where accuracy is low is consistent with our Bayesian analysis: when an LLM is highly uncertain about the task, we expect that its likelihood estimates would be close to uniform, so that its predictions would effectively be sampled from its prior—a process that would be likely to produce high-probability sentences such as famous quotations.

### Pig Latin.

Pig Latin is a secret “language” based on English. To convert an English sentence into Pig Latin, the first consonant cluster of each word is moved to the end of the word and then *-ay* is added to the end of the word. We compared Pig Latin to a system that we made up called Boar Etruscan that instead uses *-uv* as the letter pair that is added at the end. For instance, the English word *main* would become *ainmay* in Pig Latin or *ainmuv* in Boar Etruscan.

We tested LLMs on both encoding sentences into these fake languages and decoding sentences from these fake languages. The results are in Fig. 9, in which Pig Latin is the “common” variant and Boar Etruscan is the “rare” one. For encoding, GPT-3.5, GPT-4, and Claude 3 performed much better on Pig Latin than Boar Etruscan, a difference that was statistically significant (P<0.01); the other models showed no significant difference, likely due to floor effects (their accuracy was near 0% for both variants). For decoding, all five models scored better on Pig Latin than Boar Etruscan, but the difference was significant only for GPT-4 and Llama 3 (P<0.05).

In addition to this binary comparison of attested vs. unattested, Pig Latin also provides an opportunity for a finer-grained comparison because there are several variants of Pig Latin that have varying levels of commonness (40). All the major variants handle consonant-initial words the same (by moving the initial consonant cluster to the end and adding *-ay*, such as turning *frog* to *ogfray*), but they differ for vowel-initial words: all the variants add some fixed syllable at the end of vowel-initial words, but the identity of that syllable varies. We identified all mentions of Pig Latin in the Pile dataset (41), as a proxy for the training data of GPT models and found 68 cases where the added syllable was *-way*, 46 where it was *-ay*, 26 where it was *-yay*, and 8 where it was *-hay*; we found similar proportions in the C4 dataset (36). We tested LLMs on these four variants, plus a fifth one where the added syllable was *-say* (which has 0 occurrences in C4 and the Pile). As an example of how these variants differ for vowel-initial words, the word *and* could become *andway*, *anday*, *andyay*, *andhay*, or *andsay*, depending on the variant used.

**Fig. 8.**
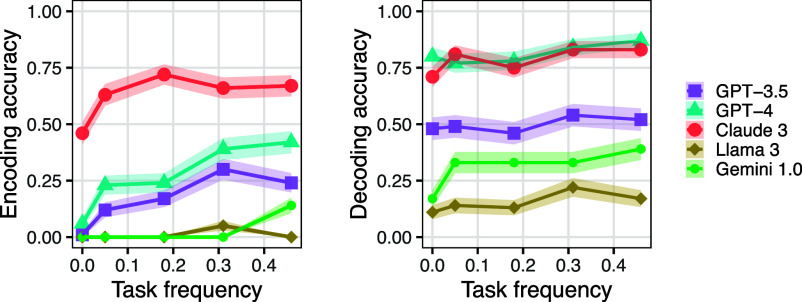
Accuracy on Pig Latin variants when encoding (*Left*) or decoding (*Right*), as a function of the variant’s frequency. The shaded intervals show one SE.

We found that (particularly for encoding) model performance patterns with the corpus frequency of the Pig Latin variant (Fig. 8). The effect of variant was significant for encoding (P<0.01) for all models except Llama 3 (P=0.19), but for decoding, it was significant only for Claude 3 (P<0.01) and Gemini 1.0 (P<0.01). Variant frequency might matter less for decoding than encoding because models may find it easier to remove an unfamiliar ending (what must be done when decoding a rare variant) than to produce an unfamiliar ending (what must be done when encoding a rare variant). This finding is striking because all five of these variants are very similar: they treat most words identically because they differ only for vowel-initial words; and even in these cases, they differ from each other only in a single letter. Nonetheless, these differences are enough to yield markedly differing performance along the lines we predicted.

### Acronyms.

**Fig. 9.**
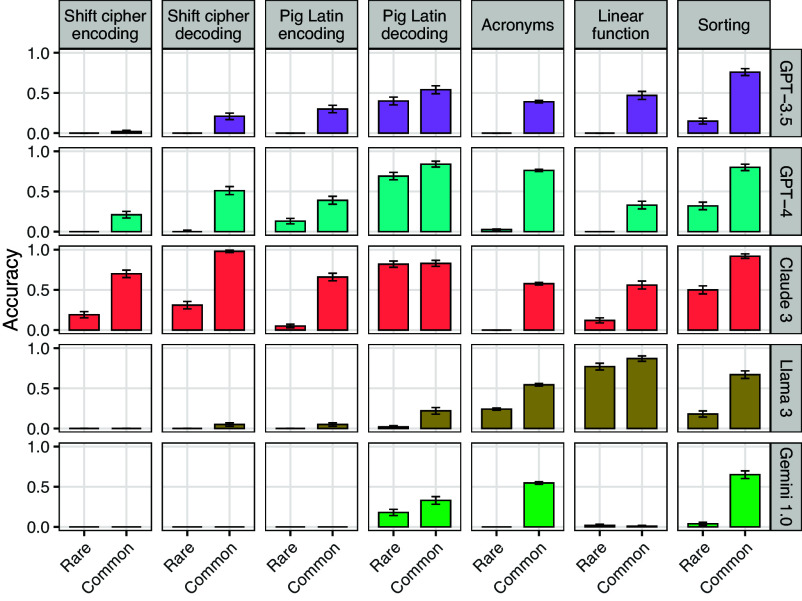
Comparing performance on common vs. rare versions of tasks. For shift ciphers, the common variant that we illustrate is rot-13, and the rare variant is rot-12. For Pig Latin, the common variant is Pig Latin, and the rare variant is Boar Etruscan (see text). For acronyms, the common variant is acronyms formed from the first letters of words, while the rare variant is acronyms formed from the second letters of words. The common linear function is f(x)=(9/5)x+32 (which is the Celsius-to-Fahrenheit conversion), and the rare linear function is f(x)=(7/5)x+31. The common version of sorting is sorting in alphabetical order, while the rare variant uses reverse alphabetical order. Error bars show one SE.

People frequently join together the first letter of each word in a sequence of words, but it is rare to join the second letter of each word. We therefore use first-letter acronym formation as a common task (e.g., producing *RESPOND* from *revolve edifice scrappy panicky outlast negated drizzle*) and second-letter acronym formation as a rare task (e.g., producing *RESPOND* from *prequel leaping ascetic splurge policed invader edifice*). For all five models, performance was much higher on first-letter acronyms than second-letter ones (Fig. 9), and the effect was statistically significant ($P<10^{-4}$).

### Linear Functions.

We compared two tasks where models had to apply a linear function to a number. As a common function, we used f(x)=(9/5)x+32; this function occurs frequently in natural text because it is the function that converts temperatures from Celsius to Fahrenheit, so text written for multinational audiences often includes nearby pairs of numbers illustrating both x and f(x). As a rare function, we used f(x)=(7/5)x+31, which has no special significance and thus does not show up often in natural text. GPT-3.5, GPT-4, and Claude 3 scored reasonably well for the common function yet poorly for the rarer one (Fig. 9), a difference that was statistically significant for all three models (P<0.01). Llama 3 performed slightly better on the common function than the rare one, but this difference was not significant (P=0.22), and Gemini 1.0 performed poorly on both functions.

### Sorting.

We gave models a list of words and told them to sort it into either alphabetical or reverse alphabetical order. Based on an analysis of the C4 corpus, we estimate that alphabetical order is 150 times more common than reverse alphabetical order in Internet text. Paralleling this frequency difference, all five LLMs perform better on alphabetical order than reverse alphabetical order (Fig. 9; $P<10^{-5}$ for all models).

### Summary of Task Probability Effects.

Across seven pairs of tasks, models performed substantially better on common task variants than rare ones. Contemporaneous work by Wu et al. (42) drew similar conclusions: they found that LLMs performed better on the default version of a task (e.g., executing Python code under the true assumption that Python uses 0-based indexing) than on a counterfactual version of the task (e.g., falsely assuming that Python uses 1-based indexing). We view our work as highlighting a general phenomenon of which Wu et al.’s conclusion is a special case: We show that LLMs are sensitive to task probability, a factor that encompasses the default-vs.-counterfactual situations that Wu et al. study but also includes other cases where there is no default, such as linear functions. As part of our more direct focus on probability, we used corpus analyses to measure task frequency for several of our experiments, a type of analysis that was not used in Wu et al.’s work. Overall, because our work and Wu et al.’s use nonoverlapping sets of tasks and draw compatible conclusions, we view these two papers as mutually reinforcing.

## Discussion

Our experiments highlight two scenarios where AI practitioners should be careful about using LLMs. First, we have shown that LLMs perform worse on rare tasks than on common ones, so we should be cautious about applying them to tasks that are rare in pretraining data. Second, we have shown that LLMs perform worse on examples with low-probability answers than ones with high-probability answers, so we should be careful about using LLMs in situations where they might need to produce low-probability text. Overcoming these limitations is an important target for future work in AI. *SI Appendix*, section C also describes several other properties of LLMs that relate to the problem they were trained to solve, including sensitivity to wording, difficulty on tasks that depend on meaning (43–46), and limited compositionality and systematicity (47–51).

Our experiments were inspired by a teleological analysis—an analysis aimed at understanding a system by understanding the problems that it was trained to solve. Our results therefore demonstrate the usefulness of the teleological approach as a way to illuminate important properties of a model.

### Understanding a New Type of Intelligence.

A range of proposals have been made regarding how to think about LLMs ( 52, 53, 54, e.g., refs.][). To understand the benefits of the teleological approach, it is helpful to compare it to a more prevalent approach that we call desideratum-focused evaluation, in which the evaluator tests for properties that they wish for models to have, such as the ability to perform certain tasks (e.g., refs. 55 and 56) or the avoidance of a particular type of error (e.g., refs. 57 and 58). Crucially, this approach is model-agnostic: what we want from a model is not driven by that model’s nature. Therefore, the desideratum-driven approach runs the risk of missing model-specific properties that one would not think to check for based on a generic characterization of the desired behavior. For example, desideratum-focused tests for rot-13 would be likely to miss models’ sensitivity to answer probability because probability is not part of how humans usually discuss this task. In contrast, the teleological approach starts with an analysis of the model and is therefore well-suited for capturing the ways in which a model’s behavior is influenced by its nature.

An additional benefit of the teleological approach is that it reveals unifying principles behind why systems do what they do (10–13). Such explanatory principles are useful because they enable us to make general predictions about the sorts of scenarios that models are likely to handle well or poorly.

To be clear, we are not arguing against testing for desiderata—doing so answers important questions about whether models are doing what we want. Rather, we are arguing that such testing should be informed by a teleological analysis because this analysis can reveal nonobvious ways that models are likely to deviate from the desired behavior.

### Comparing Models to Humans.

We have argued that, to understand LLMs, we should approach them on their own terms rather than evaluating them in the same ways that we test humans. But what if your goal is to compare LLMs to humans? Even in that case, we argue that the teleological perspective is important; much as it should inform testing for desiderata, it should also inform comparisons to humans. Specifically, we argue that human-likeness can be better assessed by the two-step process in (5) than the direct process in (4):

(4) Suboptimal approach for investigating human-likeness: Ask “is this model like a human?”

(5) Better approach for investigating human-likeness:

a. First, use teleologically motivated experiments to characterize the model in its own terms.
b. Then, ask “in what ways is this characterization like a human and unlike a human?”

The direct approach in (4) is suboptimal because the natural way to pursue this path is to evaluate models by using tests developed for humans. Such an evaluation may overestimate similarities to humans because, for many tasks, there is only one way to be correct yet many ways to be incorrect. Thus, to the extent that models are accurate, they will likely be accurate in human-like ways; and to the extent that they make mistakes, a test that is designed for humans may only highlight the sorts of mistakes that humans are liable to make, omitting potential failure modes that are unique to models. The teleological approach in (5) mitigates this risk by evaluating models in a way that is less biased by our view of human cognition.

We have focused on the first step of (5). Without a fair comparison (59–63) that evaluates humans in the same settings, we cannot make strong claims about whether the properties listed in Table 1 make LLMs qualitatively unlike humans. Prior work has shown that some of these properties are present in humans to some extent; e.g., in at least some cases, humans perform better on a task they were trained on than on a similar but new task (64, 65), and a human’s ease of processing a sentence is influenced by that sentence’s probability (66–68)—mirroring the effects of task probability and example probability that we have observed in LLMs. Nonetheless, we suspect that humans are less sensitive to these factors than LLMs, in part because of the diversity of the tasks that humans must perform; that is, although next-word prediction likely plays a role in human cognition (8, 69, 70), humans are also faced with many other tasks. In particular, we expect that humans are better than LLMs at using abstract algorithms that can be equally well applied across task variants (e.g., across different shifts in a shift cipher); humans can use such strategies by leveraging working memory and explicit reasoning, mechanisms that may be less available to LLMs than to humans (71). If this view is accurate, humans would not show such stark differences across tasks as those we have found for LLMs.

### Evaluating Models Fairly: Prompts and Scaling.

All of our experiments have used basic prompting, in which models are simply given a query. For many of our tasks, it is likely that performance could be improved by more advanced prompting techniques; e.g., Wei et al.’s chain-of-thought approach (26) substantially improved performance on last-letter concatenation, which is similar to our acronym task. However, it would not invalidate our conclusions if there are conditions in which LLM performance is greater than what we have observed. We do not claim to be highlighting fundamental incapabilities of LLMs but rather are claiming that some tasks and examples are harder for LLMs than others. Therefore, the existence of any setting that yields the predicted performance differences supports our claims, even if other settings exist where LLM performance is at ceiling for both conditions being compared.

As a first step toward investigating other prompting techniques, we used chain-of-thought prompting and step-by-step prompting to evaluate GPT-4 on shift ciphers. We found that these methods can indeed substantially increase performance, but the basic trends that we have identified (sensitivity to task frequency and output probability) still hold, just with higher overall levels of accuracy; see *SI Appendix*, section D.

A similar conclusion applies to increasing model size. Across almost all tasks, we have observed that GPT-4 substantially outperforms GPT-3.5, showing that increased model scale can provide large benefits on the tasks we have studied. However, GPT-4 still displayed the same qualitative trends as GPT-3.5 (sensitivity to task probability and example probability). Therefore, like prompting techniques, it appears that scaling may improve overall performance but may not fully overcome the ways in which models are influenced by their nature.

We also investigated the effects of few-shot learning, a popular technique for improving LLM performance in which the LLM is given several examples of correct input–output pairs before it is given the input that it must translate to an output. We found that LLMs that underwent few-shot learning continued to be influenced by task frequency and example probability (*SI Appendix*, section B). We also found that LLM performance was influenced by the nature of the examples that were provided: in some cases, LLMs benefitted more from examples that were similar to the one it had to process than examples that were not. These results support the teleological perspective by suggesting that LLMs are influenced by pressures that arise at all stages of their training: we observed effects attributable to the initial training stage of next-word prediction as well as effects attributable to the task-specific examples used in few-shot learning. In this paper, we have focused on one stage of training—the next-word prediction stage—but future work could further investigate other stages such as instruction tuning.

One approach that is likely to perform well on our tasks is to enable LLMs to execute computer programs, since computer code is well suited for handling structured tasks such as those we have focused on (72). Indeed, a recent version of GPT-4 has been augmented with the ability to execute code, and we have anecdotally found that when it utilizes this feature, it can perform much better on our tasks. The fact that LLMs augmented with code execution can score well on our tasks does not invalidate the claims of our paper, because we do not claim that our tasks are impossible for AI. Rather, our point is that we can understand AI systems by reasoning about their nature. The fact that a code-executing system can handle our tasks well—and the fact that augmenting with code execution seems to be necessary—supports our main argument: on our tasks, we expect a next-word prediction system to perform poorly, while we expect Python code to perform well.

Our results have focused on ways in which the teleological perspective illuminates shortcomings of LLMs, but being critical of LLMs is not our goal. Instead, our goal is to promote a perspective that accurately captures the properties of LLMs, both positive properties and negative ones. Though in many cases this perspective has led us to point out model weaknesses, in some ways, it makes LLMs more impressive than they would otherwise seem. Once we fully recognize that LLMs are statistical next-word prediction systems, it becomes remarkable that they can perform rot-13 or acronym formation at all, even if they do not perform these tasks perfectly.

### Conclusion.

Recent paper titles have made many statements about what language models are:

- “Language Models are Unsupervised Multitask Learners” (15)
- “Language Models are Few-Shot Learners” (73)
- “Language Models are General-Purpose Interfaces” (74)
- “Language Models are Multilingual Chain-of-Thought Reasoners” (75)
- “Language Models are Open Knowledge Graphs” (76)

We should absolutely recognize these advanced properties. Nonetheless, we should also remember a simpler fact: Language models are...language models! That is, they are statistical next-word prediction systems. This fact has some important consequences: For instance, as we have shown, language models have greater difficulty with infrequent tasks than frequent ones, even when comparing two tasks that seem equally complex to a human; and they have greater difficulty on examples with low-probability answers than high-probability ones, even when the task is deterministic. Both of these properties—as well as the others discussed above—can be attributed to the way that LLM training focuses on the statistics of word sequences. In sum, to understand what language models are, we must understand what we have trained them to be.

## Materials and Methods

### Models.

We used the most recent time-stamped model versions that were available when we ran the experiments: gpt-3.5-turbo-0613, gpt-4-0613, claude-3-opus-20240229, gemini-1.0-pro-001, and llama-3-70b-chat-hf. We accessed them through the OpenAI API for the GPT models, the Claude API for Claude 3, the Gemini API for Gemini 1.0, and the together.ai API for Llama 3. We used a temperature of 0.0; see *SI Appendix*, section H.7 for discussion.

### Stimuli.

For sentence-based tasks, the high-probability stimuli were sentences from Global Voices, a news service that shares its content under a permissive license that allows sharing and modification. Medium-probability sentences were created by taking the high-probability sentences and using RoBERTa (77) to replace some of the words with others that had a low probability in that context (but were still grammatical). Low-probability sentences were created by shuffling the words of the medium-probability examples, except that the first and last words were left in place.

For tasks based on words, the words were drawn from the CMU Pronouncing Dictionary (http://www.speech.cs.cmu.edu/cgi-bin/cmudict). The acronym inputs were lists of 7 words, the sorting input lists varied in length from 10 to 20, and the counting input lists varied from length 1 to 100. For linear functions, inputs were sampled uniformly from the integers from 0 to 999. For multiplication, each input number was sampled uniformly from the integers from 100 to 999. For the birthday task, public figures and their birthdays were obtained from the WikiBio dataset (78).

The inputs to models included a prompt describing the task to be performed, with an example provided for tasks where an example would help to avoid ambiguity (namely, article swapping, reversal, the keyboard cipher, shift ciphers, and Pig Latin). The sample size was 1,000 for each condition in the acronym task, 30 for each number in the counting task, and 100 for each condition in all other tasks. All stimuli are available on the project GitHub (see below).

### Statistical Tests.

To determine the statistical significance of differences in task variants or example probability, we used logistic regressions where the response variable was 1 if the model produced the correct answer or 0 otherwise. The predictors generally included input probability, output probability, input length, and output length, as well as task variant for conditions where we compared task variants; the predictors other than the one of interest were included to check whether the factors of interest had a significant effect even when potential confounding factors were taken into account. Some predictors were excluded when they were not relevant. See *SI Appendix*, section I for details of all significance tests.

## Supplementary Material

Appendix 01 (PDF)

## Data Availability

All of our materials are publicly available on GitHub (https://github.com/tommccoy1/embers-of-autoregression) (79) with a time-stamped release on Zenodo (https://zenodo.org/records/13763259) (80).

## Footnotes

^*^We use the term *word* loosely. More precisely, LLMs predict what token will come next, where tokens are units of text that often correspond to whole words but sometimes are smaller pieces.
