# Supplementary material for: Embers of autoregression show how large language models are shaped by the problem they are trained to solve
Source: Proc Natl Acad Sci U S A. 2024 Oct 4;121(41):e2322420121. doi: 10.1073/pnas.2322420121 (PMC11474099; doi:10.1073/pnas.2322420121)
Supplement: Supplementary file 1 — Appendix 01 (PDF) [file pnas.2322420121.sapp.pdf]

Supporting Information for

Embers of autoregression show how large language models are shaped by the problem they are trained to solve

R. Thomas McCoy, Shunyu Yao, Dan Friedman, Mathew D. Hardy, and Thomas L. Griffiths

Corresponding author: R. Thomas McCoy
Email: tom.mccoy@yale.edu

This PDF file includes:

- Supporting text (sections A through L)
- Figures S1 to S23
- Tables S1 to S8
- SI References

A Overview of this document

- Section B describes experiments involving few-shot learning.
- Section C describes several additional embers of autoregression (beyond the phenomena relating to probability sensitivity that are covered in the main paper).
- Section D provides results about using step-by-step or chain-of-thought prompting.
- Section E expands upon some of the results in the main paper by providing additional examples, discussion, or experimental conditions.
- Section F provides details of the stimuli we used when evaluating models.
- Section G provides the prompts we used when interacting with LLMs.
- Section H provides details about how we evaluated models (e.g., which types of answers were counted as correct or not).
- Section I describes the significance tests that we ran.
- Section J provides details about how we estimated the probability of specific examples.
- Section K provides details about the corpus analyses we used for estimating the frequency of different task variants.
- Section L describes the spelling test we ran to assess whether models capture the spelling of their subword tokens.

B Few-shot results

Our main results were based on tasks that were either 0-shot or 1-shot, where a task is n-shot if the model is given n examples of the task before being asked to perform it. Specifically, tasks were 1-shot in cases where we thought there were important ambiguities that could be clarified through an example—such as in shift ciphers, where we used an example to illustrate how capitalization and punctuation were handled—and tasks were 0-shot in cases where we decided that no such clarifications were necessary (e.g., counting how many letters were in a list). See Appendix G for the precise prompts that we used, which illustrate for each task whether that task was 0-shot or 1-shot. We focused on 0-shot and 1-shot experiments following (1), whose paper motivated our work: they also used only 0-shot and 1-shot experiments and emphasized that GPT-4 could perform well on a wide range of tasks in this setting, "without needing any special prompting" and "without any fine-tuning or demonstration."

Nonetheless, other authors have observed LLM performance can be further improved by giving them multiple examples (e.g., 2), in which the LLM's behavior is referred to as *few-shot learning.* There are two popular approaches for conducting few-shot learning: **fine-tuning**, in which the LLM (which has already been pre-trained on a large amount of generic training data, such as next-word prediction data) is further trained on a few examples of the target task, and **in-context learning**, in which the additional examples are provided within the prompt that is fed to the model. The critical difference between these approaches is that fine-tuning requires the model's weights to be updated via gradient descent, while in-context learning does not involve any weight updates. In this section, we extend some of our experiments to see how the trends we have observed are affected by the use of few-shot learning.

By applying the teleological perspective to few-shot learning, we make the following hypothesis: **Few-shot learning will mitigate, but not solve, the difficulties that LLMs have when handling low-probability scenarios.** On one hand, we predict that few-shot learning will mitigate these issues because we predict that models will perform better on tasks when they are directly trained to perform those tasks, and few-shot learning involves directly training the model on the task at hand. On the other hand, however, few-shot learning involves only a small amount of training data in the task at hand, which we hypothesize will be insufficient to fully overcome the idiosyncrasies imparted by the much larger amount of pre-training data that the models have received.

B.1 Models

We consider three LLMs for our few-shot experiments: GPT-3.5, GPT-4, and Claude 3. We selected these three because they were generally the strongest-performing models in our other experiments. We conducted few-shot in-context learning with all three models. We conducted few-shot fine-tuning only with GPT-3.5, since fine-tuning is not allowed with the other two models (at the time of this writing).

B.2 Tasks and data

We conducted few-shot experiments with the tasks of counting, shift ciphers, reversal, and sorting. We chose these tasks as an illustrative subset of our full set of tasks: they give us a few examples for each of the main phenomena we studied (counting, shift ciphers, and reversal allow us to analyze example probability, and shift ciphers and sorting allow us to analyze task probability). We did not use our entire set of tasks because it would have been computationally costly to do so.

The few-shot learning experiments required us to generate some additional examples for each task to be used as the examples that were provided to the LLMs; we refer to these examples as **demonstrations**. Counting and sorting both involved synthetically-generated data, so for these tasks we were able to produce more randomly-synthesized examples by running the same scripts we had used to produce examples for our basic experiments (described in Appendices F.7 and F.8). Our basic experiments with shift ciphers and reversal involved 100 sentences taken from news articles; we used the same procedures described in Appendix F.2 to extend the set to 200 sentences so that we would now have 100 sentences for evaluation and 100 sentences for use as demonstrations in a few-shot setting (specifically, we extended our dataset to 200 sentences for each probability bin—high-probability, medium-probability, and low-probability).

In all few-shot experiments, the demonstrations provided to the LLM were drawn from the same distribution as the test examples. For example, when considering a medium-probability condition, both the demonstrations and the test example were taken from the relevant medium-probability set of examples. For counting and sorting, the set of evaluation examples was the same as in our basic experiments. However, for shift ciphers and reversal, the set of evaluation experiments differed from in the main paper, a change that was necessary to ensure that the demonstrations were drawn from the same distribution as the evaluation examples. Specifically, in our basic experiments for shift ciphers and reversal, we had used the 100 highest-probability sentences in the news articles that we based the datasets on. The 100 new examples that we generated for our few-shot experiments were then the 100 next most probable sentences, which meant that they were lower probability than the 100 original evaluation sentences. To prevent this probability difference from making the demonstrations qualitatively different from the evaluation examples, we took all 200 of our sentences and randomly partitioned them into 100 for use as demonstrations and 100 for use as evaluation examples, meaning that the set of examples used for few-shot evaluation differed from the set used for the evaluations in the main paper.

Within a given experiment, we used the same set of demonstrations across all evaluation examples; e.g., when doing a 10-shot experiment with 100 evaluation examples, we used the same 10 demonstrations across all 100 evaluations. We made this decision mainly for efficiency reasons: When doing fine-tuning, using a consistent set of demonstrations meant that we only needed to fine-tune the model once and could then evaluate that single fine-tuned model on all evaluation examples. In contrast, if we had varied the set of demonstrations, we would have needed to fine-tune a different instance of the LLM for every evaluation example, which would have been time-consuming and costly.

B.3 Prompts and fine-tuning procedure

The prompts for the few-shot learning experiments can be found in Appendix G: Appendices G.3 and G.4 for shift cipher encoding, Appendices G.5 and G.6 for shift cipher decoding, Appendices G.14 and G.15 for reversal encoding, Appendices G.16 and G.17 for reversal decoding, Appendices G.25 and G.28 for counting (the same prompt—shown in Appendix G.25—was used for 0-shot counting as for the counting experiments presented in the main paper), Appendices G.33 and G.34 for sorting into alphabetical order, and Appendices G.35 and G.36 for sorting into reverse alphabetical order. In each of the aforementioned pairs, the first section gives the prompt for 0-shot experiments and the second gives the prompt for n-shot

learning with $n > 0$; these two conditions are worded slightly differently because of the presence vs. absence of demonstrations.

The in-context learning experiments proceeded exactly as the basic experiments in the main paper did, except with modified prompts (included in the appendices specified in the previous paragraph) that included demonstrations. For the fine-tuning experiments, we used the 0-shot versions of the prompts for each demonstration that was fine-tuned on and for the evaluations done after fine-tuning had concluded. We fine-tuned GPT-3.5 using the default settings in OpenAI's fine-tuning API.

We initially tried running both in-context learning and fine-tuning in three sub-conditions: 0-shot, 10-shot, and 100-shot. However, for in-context learning, we found that the 100-shot setting did not work because, in some cases, including 100 examples in the prompt made the prompt too long to be fed to the model (LLMs typically have a maximum number of tokens that their prompts can contain). Thus, for in-context learning, the three conditions we ran were instead 0-shot, 5-shot, and 10-shot; for fine-tuning, we were able to continue with our initial setting of 0-shot, 10-shot, and 100-shot.

B.4 Results

Note that the figures in this section use different conventions than the figures in the main paper and elsewhere in this supplement: the figures here use color and point shape to indicate the value of n in n-shot learning, while the figures elsewhere use these features to indicate which LLM is being tested.

Counting Output probability had a statistically significant effect on accuracy for all few-shot cases that we tested ($p < 10^{-12}$ in all cases; see Appendix I.7.1). Thus, the use of few-shot learning does not stop models from relying on output probability. Figure S1 shows counting accuracy in all few-shot settings. The 0-shot settings show clear spikes in accuracy at common numbers such as 50. Impressionistically speaking, this pattern is not as stark for the 5-shot, 10-shot, and 100-shot settings: GPT-3.5 and GPT-4 using in-context learning both have their accuracy decrease substantially in the 5-shot and 10-shot settings, removing many of these accuracy spikes, and the other two cases (Claude 3 with in-context learning and GPT-3.5 with fine-tuning) have additional new spikes arise in the few-shot settings that were not present in the 0-shot setting. Thus, few-shot learning may reduce the influence of output probability, but it does not erase the effect entirely, as shown by the fact that output probability's influence remains highly statistically significant in all cases, even if its influence is not as easy to discern in the graphs. One clear indication of its influence can still be seen, however—all cases display an accuracy spike at the value of 100.

Why do some of the few-shot settings show accuracy spikes in places that did not have such spikes in the 0-shot setting? One possibility is that models might be biased by the numbers that are shown in the demonstrations, making them more likely to guess a number if that number appeared in a demonstration (thereby increasing accuracy in cases where the correct answer appeared in a demonstration). To test this possibility, we included in our logistic regressions a predictor for how many times the correct answer appeared in the model's demonstrations. This predictor had a significant effect in two of our eight few-shot settings: GPT-4 with 10-shot in-context learning ($p < 0.001$) and GPT-3.5 with 100-shot fine-tuning ($p < 0.001$). Note, however, that this significant effect was in the expected direction only in the latter case (GPT-3.5 with 100-shot fine-tuning); in the former case (GPT-4 with 10-shot in-context learning), it was associated with lower accuracy, not higher accuracy as expected. These results support the hypothesis that, at least when fine-tuning is used, model performance in few-shot settings is influenced by the specific answers included in the demonstrations, providing a partial explanation for why the accuracy curves for the few-shot settings look different from those for the 0-shot settings. This bias toward answers seen in the demonstrations is a version of label bias, in which machine learning systems are biased toward the answers (labels) that are common in their training data; although we had one in-context learning result that displayed an effect in the opposite direction from what was expected, label bias in the expected direction has previously been demonstrated in LLM in-context learning by (3). Thus, there is evidence that LLMs performing in-context learning are at least sometimes biased in favor of the answers they have been shown in-context.

Shift ciphers In general, we found that 0-shot and few-shot models did not differ in terms of whether input and output log probability had a statistically significant effect (Figure S2, Appendix I.2.5). Across all 0-shot and few-shot cases, input log probability did not have a statistically significant effect, except in the

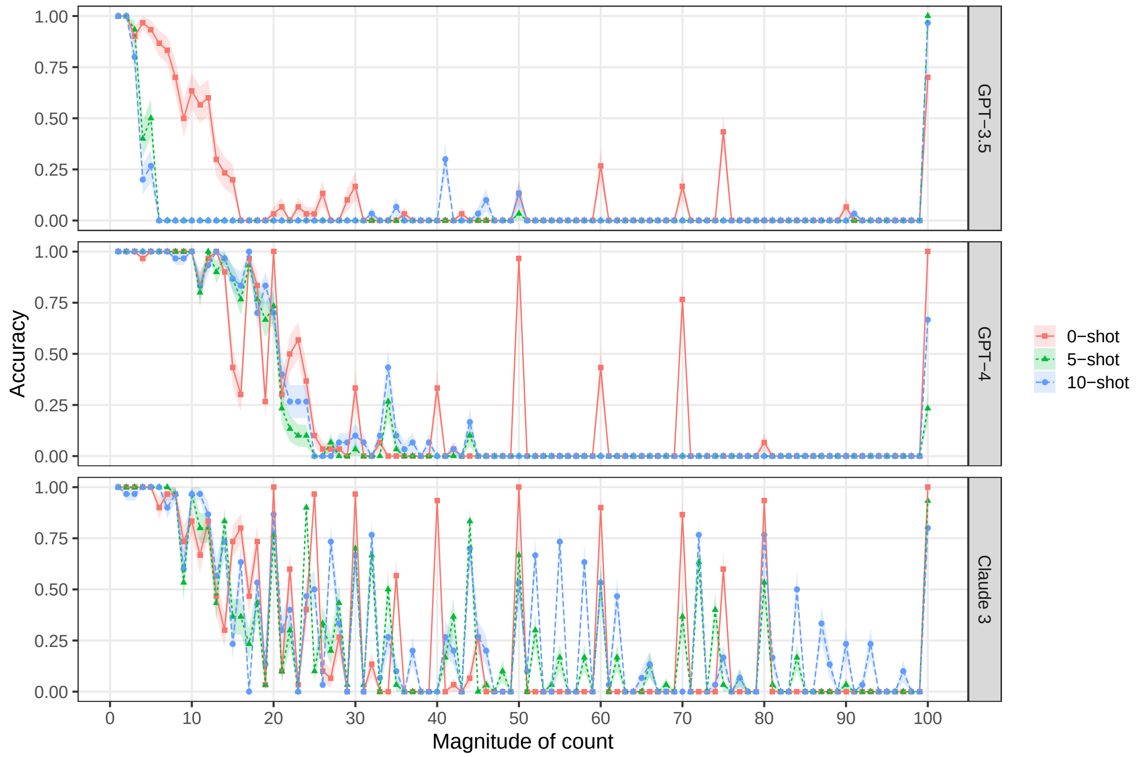

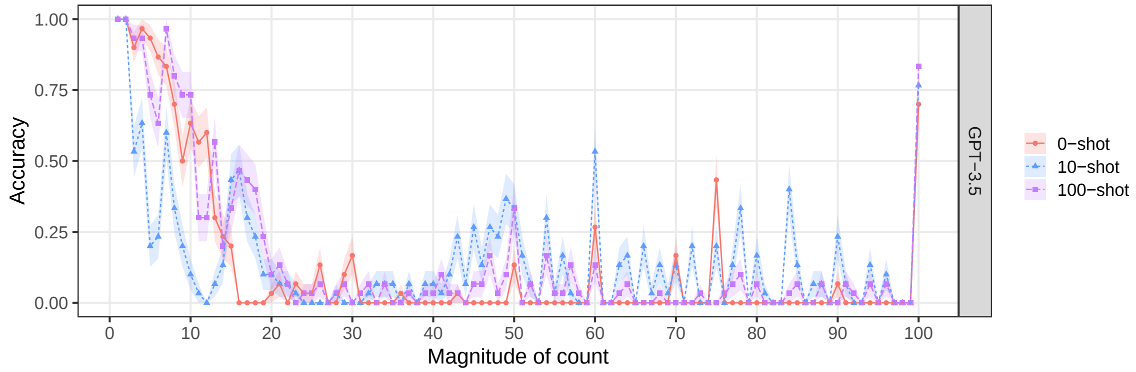

Figure S1: Accuracy at counting the words in a list for models using few-shot learning via in-context learning (top) or fine-tuning (bottom). The intervals around the lines show one standard error.

case of Claude 3 with 10-shot in-context learning ($p < 0.01$). Conversely, output log probability did have a statistically significant effect ($p < 0.05$) in all cases except for Claude 3 using 10-shot in-context learning ($p = 0.07$); the fact that Claude 3 is an exception is likely due to ceiling effects (its accuracy was close to 100% across conditions with 10-shot in-context learning). Thus, the general conclusions that we drew in the main text—namely, that output probability is expected to routinely matter while input probability is not expected to—seem to hold for models using few-shot learning for processing shift ciphers.

We then investigated the effect of shift level by comparing performance on rot-13 (a common shift level) and rot-12 (a rare shift level); see Figure S3. For the GPT models, 0-shot and few-shot conditions do not differ from each other in whether they show a significant effect of shift level: when decoding, GPT-3.5 (both with in-context learning and fine-tuning) and GPT-4 both do significantly better on rot-13 than rot-12 across 0-shot and few-shot conditions; when encoding, GPT-4 also does significantly better on rot-13 than rot-12, while GPT-3.5 (whether using in-context learning or fine-tuning) shows no statistically significant difference between the shift levels, likely due to floor effects.

For Claude 3, however, 0-shot and few-shot cases showed qualitative differences: in the 0-shot cases, Claude 3 performed statistically significantly better on rot-13 than rot-12, both for encoding and decoding, but in few-shot cases it showed almost identical performance on both shift levels. Why might Claude 3 show such similarities across the rare and common shift level when using in-context learning? We hypothesized that, when doing in-context learning, Claude 3 might use a strategy in which it leverages the precise input letter/output letter correspondences that are shown in the provided demonstrations. This strategy would yield the same accuracy on both shift levels because the demonstrations are matched across conditions in such a way that the same correspondences would be illustrated for rot-12 as for rot-13. However, this strategy does not provide an abstract, generalizable way to solve a shift cipher because it only works for the letters that are illustrated; it will not generalize to new letters even though the input/output map is systematic across letters. To test this hypothesis, we ran another set of shift cipher experiments in which the text to be encoded or decoded was now a single word instead of a whole sentence. We studied two variants of this setting. In one variant ("with overlap"), every word in the demonstration shares at least one letter with the test word. In the other variant ("without overlap"), no letters in the test word appeared in the answer word.

The results of this follow-up analysis for Claude 3 are in Figure S4, with significance tests described in Appendix I.2.5. In the "with overlap" condition, providing in-context examples substantially raises Claude 3's performance for rot-12 (for both encoding and decoding), but in the "without overlap" condition, in-context examples do not improve rot-12 performance: accuracy is essentially flat across 0-shot, 5-shot, and 10-shot conditions. This result supports the hypothesis that Claude 3's in-context learning of rot-12 is based on copying the letter correspondences it has seen rather than acquiring an abstract notion of the shift cipher at hand. In contrast, for rot-13, decoding performance improves substantially from the 0-shot to 5-shot conditions in both the "with overlap" and "without overlap" conditions, showing that Claude 3 can perform more generalizable in-context learning of rot-13 than rot-12. For encoding, however, in-context demonstrations do not substantially improve rot-13 performance. In sum, then, few-shot learning does not erase Claude's dependence on task commonness: although it can perform equally well on a common task and a rare task when the in-context demonstrations are selected such that copying from the demonstrations is helpful, there is still a statistically significant difference between the rare and common tasks when no such copying is possible.

Figure S5 collects all few-shot in-context learning results for shift ciphers across the three conditions and the three models, beyond the Claude 3 results highlighted in Figure S4. For GPT-3.5 and GPT-4, accuracy is statistically significantly higher for rot-13 than for rot-12 across all word-based cases.

Reversal Figure S6 shows model performance on reversal using few-shot learning. For all few-shot settings with in-context learning, output log probability (which was tested in the decoding settings) had a statistically significant effect ($p < 0.001$; see Appendix I.3.3). For the settings that used fine-tuning, output log probability had a statistically significant effect in the 0-shot setting but not in either few-shot setting, likely because of ceiling effects: accuracy was close to perfect in all few-shot cases. We have in general hypothesized that output probability will influence LLM performance. These results suggest that output log probability continues to be important even in few-shot settings, except in cases where the use of few-shot learning improves performance so much that models reach ceiling-level performance; when this is not the case, however, the effects of probability remains.

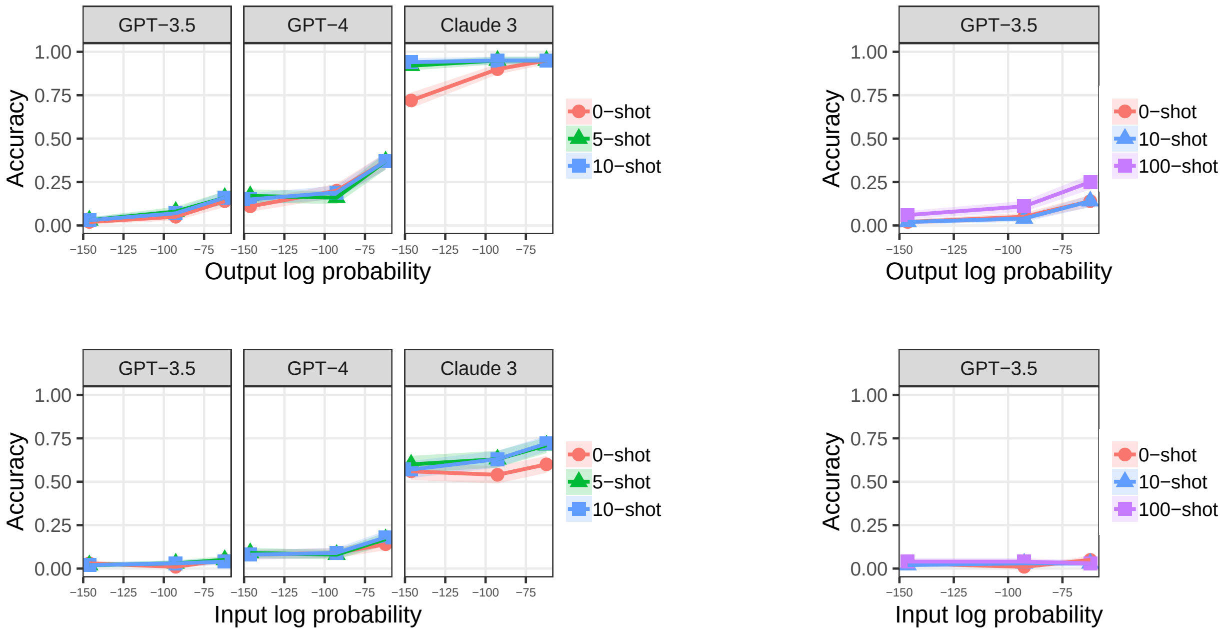

Figure S2: Few-shot shift cipher accuracy using in-context learning (left column) or fine-tuning (right column). The top row shows decoding conditions (with output probability varying), and the bottom row shows encoding conditions (with input probability varying).

We have also hypothesized that input probability would be less important in determining model performance than output log probability. In this set of experiments, we investigated input probability in the encoding settings, and the results show that it indeed can influence model performance but does so less reliably than output log probability does. Specifically, all models using in-context learning show strong effects of output log probability, but the effects of input log probability are in many cases weak or nonexistent for these models. There are, however, some cases where input log probability had a large and significant effect, such as 10-shot in-context learning with Claude 3 ($p < 10^{-15}$), showing that there are some cases within few-shot learning (as in the zero-shot setting) where input log probability matters.

Sorting We evaluated models on sorting lists of words into alphabetical or reverse alphabetical order, where alphabetical order is a more common version of sorting than reverse alphabetical order. Across 0-shot and few-shot settings, models consistently performed better on alphabetical order than reverse alphabetical order (Figure S7), and these differences were statistically significant (Appendix I.8).

B.5 Discussion

In general, the results with few-shot learning show that the core effects that we hypothesized in the main paper continue to play a role in few-shot learning scenarios. Specifically, these effects are the influence of output probability and the influence of task frequency.

Output probability We investigated output probability within the tasks of counting, shift cipher decoding, and reversal. Considering all models, tasks, and numbers of demonstrations, these experiments amounted to a total of 36 conditions. Output probability had a statistically significant effect across 33 of the 36 settings; the 3 exceptions are likely explained by ceiling effects, because all of them are cases where the use of few-shot learning brought accuracy close to 100%. From these results, we conclude that output probability significantly influences LLM performance in few-shot settings, except in cases where the LLM's performance at the task is close to perfect.

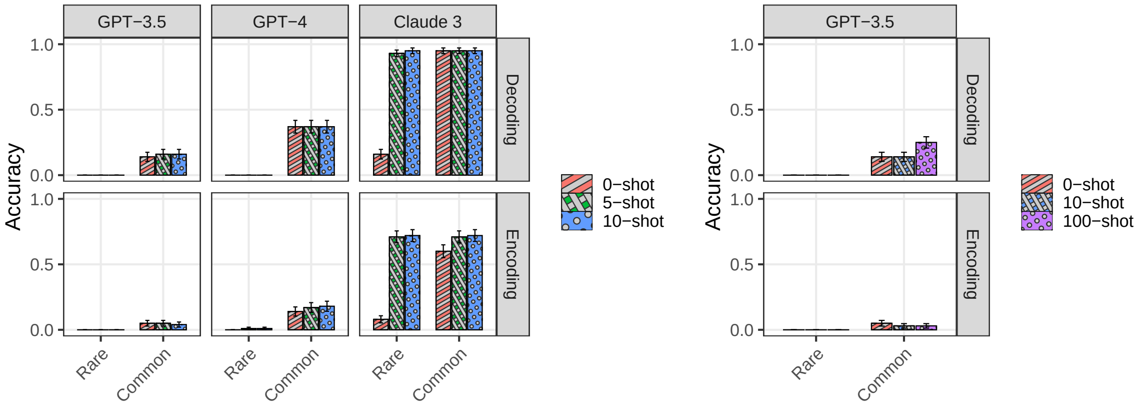

Figure S3: Few-shot accuracy on two shift ciphers: rot-12 ("rare") and rot-13 ("common") for models using in-context learning (left) and fine-tuning (right).

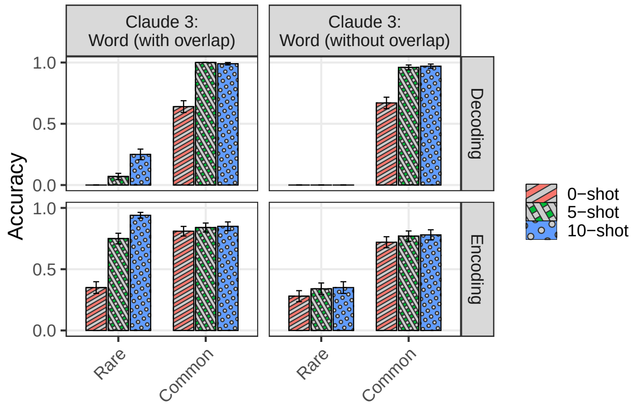

Figure S4: Few-shot accuracy on two shift ciphers: rot-12 ("rare") and rot-13 ("common") for Claude 3 using in-context learning. The stimuli used are individual words. In the left column, the demonstration words contain some of the same letters as the test word; in the right column, the demonstration words have no overlap in letters used with the test word.

Task frequency We investigated task frequency within the domains of shift cipher encoding, shift cipher decoding, and sorting. For sorting, task frequency had a statistically significant effect across all conditions that we investigated. For shift cipher encoding and decoding, task frequency also had a statistically significant effect except in two types of cases: First, for GPT-3.5, encoding performance was near 0% across task variants, meaning that floor effects prevented task frequency from having a significant effect. Second, Claude 3 showed similar performance between task variants for both encoding and decoding when few-shot learning was used, but this was likely because the type of demonstrations we provided facilitated a copying-based strategy that could be utilized equally well for either task variant that we considered. In a follow-up experiment where we modified the demonstrations to remove the possibility of a copying-based strategy (by using demonstrations that did not overlap in content with the test example), Claude 3 now showed a statistically significantly better performance for the common shift cipher than for the rare shift cipher across zero-shot and few-shot settings. Overall, then, we conclude that task frequency plays an important role in few-shot settings, just like it does in zero-shot settings.

Implications for teleological analysis The models that we considered in this setting had first been pre-trained on a large amount of data that was not specific to the tasks we considered, and they then underwent additional few-shot learning (either via in-context learning or fine-tuning) that was specific to the task at

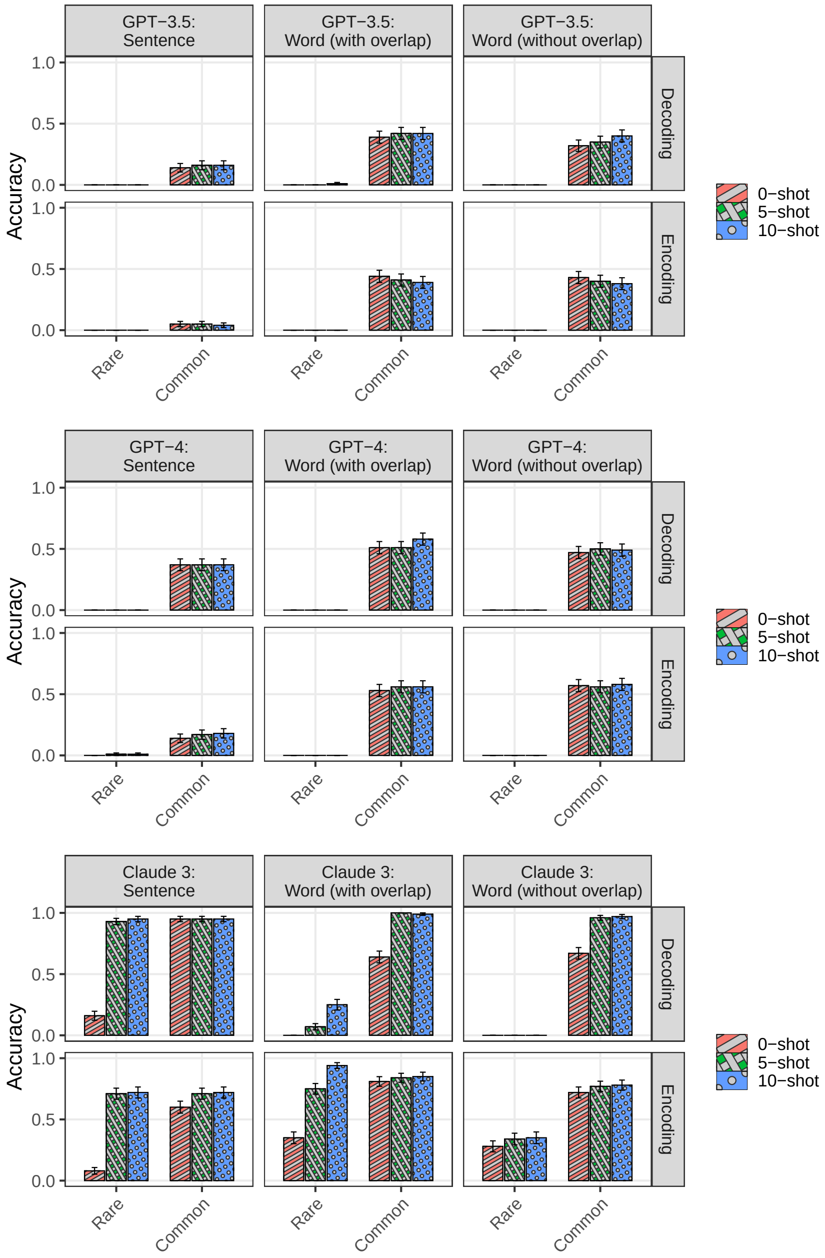

Figure S5: Few-shot accuracy on two shift ciphers: rot-12 ("rare") and rot-13 ("common") for models using in-context learning. There are three types of examples being used: whole sentences (left column of each plot), copied from Figure S3; individual words for which the demonstration words contain some of the same letters as the test word (middle columns); and individual words for which there is no overlap in letters used between the demonstration words and test word (rightmost columns). The Claude 3 word-based conditions repeat from Figure S4.

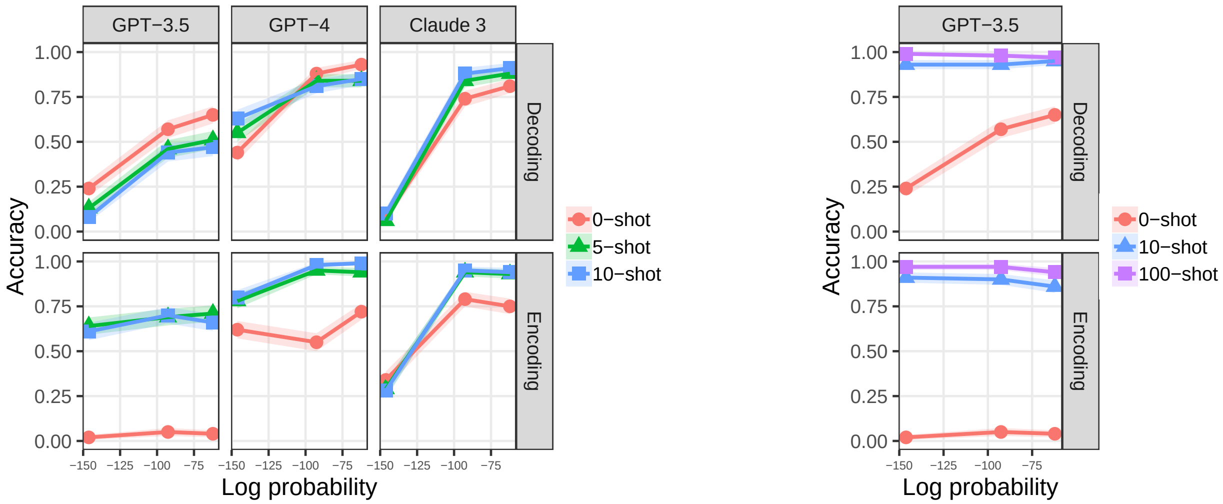

Figure S6: Few-shot reversal accuracy using in-context learning (left) or fine-tuning (right). For decoding conditions, the log probability that is shown is output log probability; for encoding conditions, the log probability that is shown is input log probability.

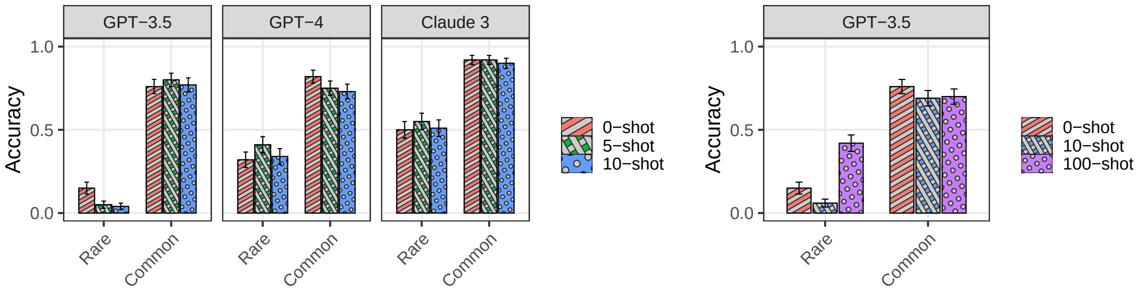

Figure S7: Few-shot accuracy on sorting into reverse alphabetical order ("rare") or alphabetical order ("common") for models using in-context learning (left) and fine-tuning (right). The error bars show one standard error.

hand. We found that models in these cases showed the same basic effects of output probability and task frequency as we saw with the models in the main text (which did not have the benefit of the additional few-shot learning). We conclude from these results that the biases that models acquire during pre-training can persist even after fine-tuning. This conclusion is also supported by our results in the main text: all the models we considered were first pre-trained on next-word prediction and then fine-tuned with instruction tuning, but our hypotheses were derived by considering only the next-word prediction portion of the training. The fact that our hypotheses were supported in the main text thus provides further evidence supporting the conclusion that biases imparted by pre-training continue to influence models even after fine-tuning. Indeed, the very fact that pre-training is useful suggests that biases from pre-training must persist to some extent through fine-tuning: if fine-tuning erased all influences of pre-training, then pre-training would serve no purpose.

We do not mean to suggest that fine-tuning and in-context learning are irrelevant for deriving a teleological understanding of LLMs. In fact, our experiments with few-shot learning provided two results that show that, when a model has undergone fine-tuning or in-context learning, the nature of the few-shot demonstrations can be important for understanding the resulting systems. First, in our counting experiments, we found that, for GPT-3.5 fine-tuned with 100 examples, the presence of a number in the provided demonstrations provided a statistically significant improvement to models' accuracy on examples with that number as a

correct answer, though note that there was also a case—GPT-4 with 10-shot in-context learning—where the presence of a number in the provided examples had a negative effect on accuracy for numbers with that answer. Second, in Claude 3's few-shot performance with shift ciphers (whether encoding or decoding), we found that it partially relied on a strategy that depended on which specific letter-letter correspondences had been illustrated in the demonstrations. In both of these cases, the nature of the few-shot demonstration data played an important role in model performance, much as we argued in the main text that the nature of pre-training data also plays an important role in model performance.

Overall, then, our conclusion is the following: A complete teleological analysis of an LLM should be informed by all of the pressures that have shaped that LLM, whether those pressures have come from pre-training, fine-tuning, or in-context learning. Considering all of these influences can be time-consuming, but we have also shown that productive insights can be gained even by considering just one type of influence—for instance, most of our hypotheses in the main paper were based on considering just the pre-training data but not the data used for instruction tuning, and these hypotheses were well-supported by experimental results. An important direction for future work is to extend the preliminary investigations made in this section about the ways in which pressures from pre-training interact with pressures from fine-tuning.

C Other embers of autoregression

In this section, we discuss several additional ways in which LLMs are influenced by what they are trained to do, beyond the probability-sensitivity that is the focus in the main paper.

C.1 Lack of embodiment

There are some tasks that humans easily solve by relying on our presence in the physical world—e.g., by using information from sensory input, or by offloading work onto external tools and resources. However, LLMs do not have bodies and do not navigate the physical world. All of their experience is in the form of text tokens. Therefore, LLMs can be expected to struggle on the tasks that are most straightforwardly solved by leveraging one's embodiment.

C.1.1 Keyboard cipher

The keyboard cipher is a cipher in which each letter is replaced with the letter to the right of it on a QWERTY keyboard (wrapping around if necessary); e.g., *Good morning* becomes *Hppf zptmomh.* A human with access to a keyboard can easily apply this cipher by referring to the keyboard. However, LLMs do not inhabit the physical world and therefore do not use a keyboard to produce their answers, so we therefore expect for them to struggle at this task. To test this prediction, we asked LLMs to encode our 100 high-probability sentences (Section F.2) into the keyboard cipher. For this experiment, we used a chain-of-thought prompt (shown in Figure S8) because our prediction is an absolute one rather than a relative one and therefore merits testing models in the setting most favorable to them. As predicted, all five LLMs scored 0% on this task (Table S1, "basic prompt").

To some extent, this result may seem obvious—it may not be surprising that LLMs cannot perform this task without a keyboard in front of them. However, if we modify their prompt to include a textual representation of the keyboard layout (shown in (1) below; see Appendix G.44 for the full prompt), performance

	GPT-3.5		GPT-4		Llama 3		Claude 3		Gemini 1.0	
	Acc.	Edit dist.	Acc.	Edit dist.	Acc.	Edit dist.	Acc.	Edit dist.	Acc.	Edit dist.
Basic prompt	0.00	21.5	0.00	18.0	0.00	27.5	0.00	10.0	0.00	47.5
Keyboard provided	0.00	17.5	0.01	8.0	0.00	25.0	0.01	9.0	0.00	38.5
Correspondences provided	0.00	51.5	0.12	4.0	0.01	12.0	0.19	2.0	0.00	18.0

Table S1: Performance on the keyboard cipher. For accuracy (which is abbreviated as *acc.*), higher is better; for edit distance (which is abbreviated as *edit dist.*), lower is better. The edit distances are medians to address the presence of outliers.

Keyboard cipher

Prompt:
The keyboard cipher is a cipher where each letter is replaced with the letter to the right of it on a QWERTY keyboard. If the letter does not have a letter to its right, then you should wrap around to the start of the row; e.g., "p" maps to "q". For example, here is a message to be encoded:
Original message: "Hello world!"

To encode this message, we replace each letter with the one to the right of it on a QWERTY keyboard:
1. The first word is "Hello"
  * H -> J
  * e -> r
  * l -> a
  * l -> a
  * o -> p
  So "Hello" becomes "Jraap"
2. The second word is "world!"
  * w -> e
  * o -> p
  * r -> t
  * l -> a
  * d -> f
  * ! -> !
  So "world!" becomes "eptaf!"

Therefore, the message in the keyboard cipher is: "Jraap eptaf!"

Here is another message. Encode the message in the keyboard cipher, word by word and letter by letter. On the last line, write the words "Keyboard cipher:" followed by the encoded message:
Original message: "But this time, there may also be another reason."

Correct answer:
Niy yjod yozr, yjrtr zsu sadp nr smpyjrt trsdpm.

GPT-4 output:
Niy yjoa yo,r yjtrr ,su saap nr sbpyjrt trsapb.

Figure S8: The chain-of-thought prompt that we use to ask models to apply the keyboard cipher.

improves but is still flawed (Table S1, "keyboard provided"). For example, GPT-4's edit distance is cut approximately in half, meaning that it makes only half as many errors, but the edit distance remains relatively high (8.0, meaning that it typically makes 8 mistakes per sentence). This result shows that the models' failure is not merely due to a lack of information; even with this information provided, they are still not able to reliably use it properly—a difficulty that we conjecture can be tied back to the lack of embodiment: because they do not inhabit the physical world, they lack experience in navigating spatial arrangements such as the provided keyboard layout.

(1) q w e r t y u i o p
a s d f g h j k l
z x c v b n m

When we instead modify the prompt to specify exactly what each letter maps to (*a -> s, b -> n, c -> v, ...*), we see another jump in performance for all models except GPT-3.5 (Table S1, "correspondences provided"), supporting the argument that one reason why this is task is difficult for models is that they are unable

to extract the necessary information from an array of letters. Thus, when we provide this information directly instead of requiring the model to extract it from an array of letters, its performance increases. Even in this final case, accuracy is far from 100%; the remaining errors may be explained by difficulty with systematically manipulating symbols (see Section C.6.2). It is not clear why GPT-3.5's performance is worse in the "correspondences provided" condition than the "keyboard provided" condition. One possible explanation is that the prompt is much longer in this condition than in the others, so GPT-3.5 might have trouble sorting through all of the provided information.

Note that embodiment is not strictly necessary to impart the information required for this task: it can in principle be learned from textual input. Indeed, even in the "basic prompt" condition where models were not provided with a keyboard, most models get many letters right: if we assumed that they only got correct the letters whose correspondences were illustrated in the prompt, and guessed randomly on all others, their edit distances would be 37.0, but most models in fact display much lower edit distances (Table S1). Thus, these models appear to have learned some information about keyboard structure from their training data, just not enough to perform the task well. More generally, then, we are not claiming that text-only models could never acquire the information for which humans typically rely on embodiment. Rather, what we are claiming is that such information is difficult but not impossible for non-embodied models to acquire.

C.2 Sensitivity to wording

Because LLMs are explicitly trained on language but not on ideas, we expect them to be sensitive to the linguistic form of the ideas they encounter: they may yield varying results when given the same idea expressed in different ways. Prior work has identified several ways in which LLMs are sensitive to task-irrelevant changes in wording: they may change their response based on the addition of irrelevant information (4–6), based on textual perturbations such as lowercasing text or introducing typos (7), based on the ordering of information in the prompt (3, 8), or based on the way in which the prompt is phrased (3). In the opposite direction, LLMs are sometimes *less* sensitive to the prompt than they should be, displaying successful performance even when prompts are irrelevant or misleading (9, 10); this result further illustrates how models do not understand their instructions in the same ways that humans likely do but instead rely on some unintuitive statistical cues relating to the way the prompt is worded. Below, we provide two further experiments showing sensitivity to prompt wording (Section C.2.1) and formatting (Section C.2.2).

C.2.1 Linear function

We hypothesized that models would perform better at applying the function $f(x) = (9/5)x + 32$ when it was pointed out that this function was the Celsius-to-Fahrenheit conversion than when the function was simply presented as an abstract equation. This hypothesis is motivated by the fact that, when LLMs have previously encountered input-output pairs illustrating this function, it would usually have been in the context of Celsius-to-Fahrenheit conversion, so mentioning this context may help to activate the components of the LLM that are relevant to this function. To test this hypothesis, we used three conditions illustrated in Figure S9. In the basic condition, models were simply told to apply this function. In the primed condition, they were given the same prompt as in the basic condition, except that it was prefixed with the sentence *In this task, you must convert a number from Celsius to Fahrenheit.* Finally, the priming control condition was similar to the primed condition, but the new sentence that was added at the start was instead the generic statement *In this task, you must return an answer based on an input*; we used this condition to check whether any differences between the primed condition and the basic condition were solely due to the presence of an additional sentence at the start of the prompt.

We were most interested in models' abilities to compute the linear function's output rather than their ability to retrieve a memorized value. Accordingly, we restricted the input values to be greater than 500 and to not be divisible by 10; in a sample of Internet text from C4 (11), we found that these constraints eliminated 99.8% of examples mentioning a Celsius-Fahrenheit equivalence because people rarely discuss temperatures greater than 500°C, and when they do they often use round numbers that are divisible by 10 (see Appendix F.10). Therefore, applying these constraints restricts the evaluation to values that models are less likely to have memorized, though we cannot rule out the possibility that models may have memorized these values because we do not have access to the training corpora for the LLMs that we tested.

Linear function: Prompt variants

Basic prompt

Below is a number. Multiply it by 9/5 and then add 32. Your answer should be a single number:

**Input:**	558	731	842	977
**Correct:**	1036.4	1347.8	1547.6	1790.6
**GPT-4:**	✗ 1046.4	✓ 1347.8	✗ 1517.6	✗ 1750.6

Priming control prompt

In this task, you must return an answer based on an input.

Below is a number. Multiply it by 9/5 and then add 32. Your answer should be a single number:

**Input:**	558	731	842	977
**Correct:**	1036.4	1347.8	1547.6	1790.6
**GPT-4:**	✓ 1036.4	✓ 1347.8	✗ 1537.6	✗ 1760.6

Primed prompt

In this task, you must convert a number from Celsius to Fahrenheit.

Below is a number. Multiply it by 9/5 and then add 32. Your answer should be a single number:

**Input:**	558	731	842	977
**Correct:**	1036.4	1347.8	1547.6	1790.6
**GPT-4:**	✓ 1036.4	✓ 1347.8	✓ 1547.6	✗ 1780.6

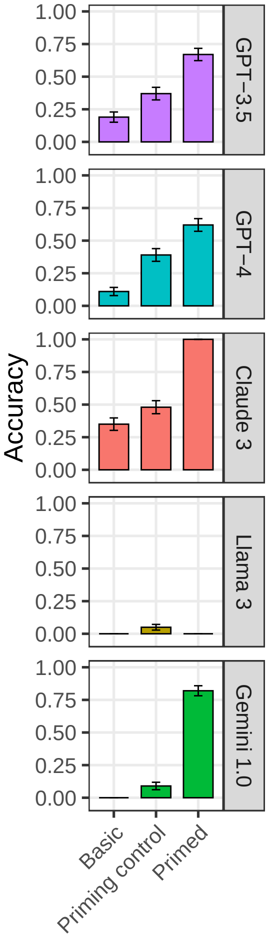

Figure S9: **Left:** The prompts used to ask models to apply the function $f(x) = (9/5)x + 32$, with example predictions from GPT-4. **Right:** Model performance on the different prompts. Error bars show one standard error.

GPT-3.5, GPT-4, Claude 3, and Gemini 1.0—that is, all models except Llama 3—performed much better when the task was framed as Celsius-Fahrenheit conversion (in the primed condition) than otherwise (in the basic and priming control conditions), as shown in the plots in Figure S9. Using mixed-effects logistic regressions, we found that these differences between the primed condition and the other conditions were statistically significant for all four models that showed non-negligible accuracy ($p < 0.01$ in all cases; see Appendix I.9.2). For GPT-3.5, GPT-4, and Gemini 1.0, the difference between the basic prompt and the priming control prompt was also significant, but this difference was not significant for Claude 3. These results further show that models' ability to apply this function is tied to the function's use in a particular scenario, as invoking that scenario improves performance.

At a more detailed level, there are two potential explanations for this result. First, it might be that models have learned the linear function in a systematic way, but their ability to access this systematic ability is increased by an explicit mention of Celsius and Fahrenheit. Second, it might be that models do not have a systematic ability to compute this function but have instead memorized many Celsius-Fahrenheit pairs. Under this account, mentioning the words *Celsius* and *Fahrenheit* is not only helpful but in fact necessary—models have no way to connect the two numbers except in the context of a memorized temperature-related pair. By using rare types of input values, we have attempted to reduce the chance of finding results that only illustrate a memorization-based explanation, pointing instead toward the explanation that posits systematic

abilities that can only be accessed under certain settings, but we cannot rule out the memorization-based explanation: these systems have been trained on so much data that they likely have had opportunities to encounter rare temperature values.

Perhaps the most surprising result in this set of analyses is the fact that GPT-3.5, GPT-4, and Gemini 1.0 performed better in the priming control condition than in the basic condition. These differences were statistically significant for all three models (see Appendix I.9.2). We do not see any way in which the priming control prompt provides more information than the basic prompt, since all of the information in the initial sentence of the priming control prompt is already apparent in the basic prompt by virtue of its structure. This finding shows how models can be highly sensitive to wording choices, reinforcing the point that they are primarily models of word sequences rather than idea-manipulating models.

C.2.2 Multiplication

We gave models a multiplication problem where both numbers being multiplied were random three-digit numbers. There were four conditions, corresponding to different ways of writing the numbers—using digits, lowercase words, all-caps words, or alternating capitals. See Figure S10 for examples. The values of the numbers were identical across the conditions.

Model accuracy often varied substantially depending on how the numbers were formatted (Figure S10, right), and many of the differences between formats were statistically significant (see Appendix I.10). This result illustrates how the models are, first and foremost, language models rather than math models or reasoning models; as a result, the same task expressed in different linguistic forms can be handled very differently by models.

C.3 Difficulty estimating an example's difficulty

In addition to prompt engineering and scaling, another way in which embers of autoregression could potentially be mitigated would be to ask the LLM how difficult the task at hand is for it. Ensuring that LLMs correctly report when they have difficulty is an ongoing area of research (12, 13); to the extent that an LLM is well-calibrated in this regard, its judgments could allow us to recognize when we should be cautious about its outputs (e.g., we might take a judgment that difficulty is high as a sign that we should verify the response with a human or pass the query to a symbolic interpreter that can robustly handle the task). As an initial step toward investigating whether LLMs recognize that the tasks we have studied are difficult for them, we considered five queries for each of three tasks where we found LLMs to struggle:

- Counting a list of 29 letters.
- Performing the article swapping task in the low-probability setting.
- Decoding a message written in rot-10, where the correct answer is a high-probability sentence.

For each of these cases, we tested GPT-4 via the online chat interface (accessed on September 20, 2023) on five examples. Of the fifteen total cases, there were two that GPT-4 answered correctly (both within the counting task); since we are most interested in cases where GPT-4 was incorrect, we regenerated those responses, which yielded an incorrect answer for both. Each of the fifteen queries was asked in a new chat session. After we received GPT-4's incorrect response, we then asked it, "On a scale from 1 to 10—where 1 is easy and 10 is hard—how difficult was this question to answer for you?" In all fifteen cases, GPT-4 responded that the question had a difficulty level of 1—i.e., very easy.

Two examples of GPT-4's difficulty estimates are in Figure S11. In the first case, it states that the task is "straightforward...for a computer program." In the second case, it refers to what its "algorithms" can perform easily. One possible interpretation of these responses is that GPT-4 is estimating difficulty based on a generic definition of computation, rather than by considering its own specific capacities and limitations. Perhaps, then, GPT-4 itself could benefit from the teleological perspective, as this perspective emphasizes approaching systems on their own terms.

Three-digit multiplication: Input format

Digits

Input: `285 times 796`
Correct: `226860`
✓ **GPT-4:** `226860`

Lowercase words

Input: `two hundred eighty-five times seven hundred ninety-six`
Correct: `226860`
✓ **GPT-4:** `226860`

All-caps words

Input: `TWO HUNDRED EIGHTY-FIVE times SEVEN HUNDRED NINETY-SIX`
Correct: `226860`
✗ **GPT-4:** `226620`

Alternating capitals

Input: `tWo HuNdReD eIgHtY-fIvE times sEvEn HuNdReD nInEtY-sIx`
Correct: `226860`
✗ **GPT-4:** `226960`

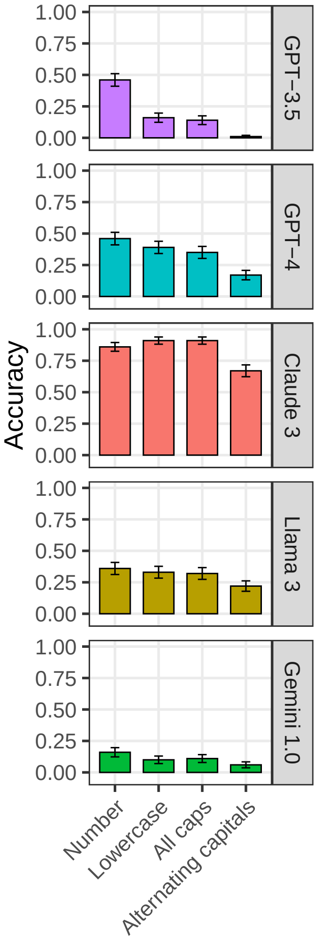

Figure S10: **Left:** Example stimuli for multiplication problems with different types of input formatting. **Right:** Accuracy on multiplication using the different types of number formats. The error bars show one standard error.

C.4 Phenomena relating to the training task

The idea that a system is crucially influenced by the function it performs has a long history in cognitive science (14–16). One important consequence of this idea is that two different systems that have the same goal might converge to similar strategies even if they are implementationally very different. This fact can help us to understand one system by leveraging what we know about a different system that achieves the same goal. For instance, (17) gained insight into the gradient-based meta-learning approach MAML (18) by demonstrating a relationship to hierarchical Bayesian modeling, and (19) illuminated processing strategies of the LLM BERT (20) by showing that, faced with the task of modeling natural language, BERT arrived at a similar analysis pipeline as NLP practitioners who had faced the same goal. Such parallels can also be drawn in the opposite direction: instead of hypothesizing that two systems will work in similar ways because they have similar goals, we can infer that two systems may be shaped by similar goals based on the fact that they work in similar ways—a type of inference at the heart of some work making inferences about the human brain based on similarities to computational models (21, 22). Perhaps most relevant to our perspective is the argument from (23) that those who develop and use LLMs must continually remind themselves of what the LLMs were trained to do, in order to avoid anthropomorphizing these systems; avoiding this temptation was one of our main reasons for pursuing a teleological approach to understanding LLMs.

Difficulty calibration

Experimenter:
How many letters are in the following list? “bbbbbbbbbbbbbbbbbbbbbbbbbbbbb”

GPT-4:
The list provided contains 28 “b” letters. *[Note: The correct answer is 29]*

Experimenter:
On a scale from 1 to 10 - where 1 is easy and 10 is hard - how difficult was this question to answer for you?

GPT-4:
For me, answering that question would be a 1 on the scale from 1 to 10 in terms of difficulty. Counting the number of characters in a string is a straightforward task for a computer program.

Experimenter:
Rot-10 is a cipher in which each letter is shifted 10 positions forward in the alphabet. For example, here is a message written in rot-10 along with the original text that it was created from:
Rot-10 text: “Cdki robo!”
Original text: “Stay here!”

Here is another message in rot-10. Decode this message to produce the original text:
Rot-10 text: “Dryco gry exnobcdkxn dro boqsyx gsvv qod grkd S wokx pbyw drsc cswzvo zrbkco.”
Original text:

GPT-4:
To decode a ROT-10 cipher, you would shift each letter 10 positions backward in the alphabet. Numbers, spaces, and punctuation marks remain the same.

Here’s how to decode your ROT-10 message:

Rot-10 text: “Dryco gry exnobcdkxn dro boqsyx gsvv qod grkd S wokx pbyw drsc cswzvo zrbkco.”

1. For “D”, shifting 10 positions backward gives “A”.
2. For “r”, shifting 10 positions backward gives “i”.
3. For “y”, shifting 10 positions backward gives “m”.
4. For “c”, shifting 10 positions backward gives “w”.
5. For “o”, shifting 10 positions backward gives “e”.

Following this pattern for the entire sentence, the original text becomes:

Original text: “After you understand the method used for this simple message.”

I’ve shifted each letter 10 positions backward in the alphabet to decode the ROT-10 message into its original text.

[Note: The correct answer is “Those who understand the region will get what I mean from this simple phrase.”]

Experimenter:
On a scale from 1 to 10 - where 1 is easy and 10 is hard - how difficult was this question to answer for you?

GPT-4:
For me, the question is very straightforward to answer, so on a scale from 1 to 10, it would be a 1 in terms of difficulty. My algorithms are designed to perform tasks like decoding ROT ciphers relatively easily.

Figure S11: Asking GPT-4 to rate the difficulty of queries. These responses are from GPT-4’s online chat interface, accessed on September 20, 2023.

In addition to helping us understand systems, the importance of a system's goal is also helpful in work aimed at improving models. First, many authors have studied which (pre-)training objectives are most effective for producing desired behavior in a model (11, 24, 25). Second, beyond differences in general training tasks, the influence of a system's goal in driving that system's nature is substantial enough that models can be given highly targeted training objectives that can override a model's inherent inductive biases, causing one type of system to behave like another type of system. In this way, it is possible to distill a symbolic hypothesis space (26) or a probabilistic model (27, 28) into a neural network, or to distill the inductive biases of one type of neural network into another (29).

The next subsections discuss two specific ways in which the LLM training task of next-word prediction influences their behavior.

C.4.1 Difficulty in tasks that depend on meaning

Since LLMs are trained to manipulate sequences of words, they are primarily models of language rather than models of thought (30). They do not directly manipulate ideas or structured world models; to the extent that such processing occurs in LLMs, it has to emerge indirectly. LLMs therefore sometimes struggle in contexts that could be naturally handled by using and updating a model of entities in the world; e.g., they sometimes fail to track entities in a discourse (31, 32) and are sometimes insensitive to the ways that negation affects statements about entities and their properties (33). Outside of particular linguistic phenomena, LLMs also display shortcomings in tasks that require knowledge of the causal properties and affordances of everyday objects (34, 35).

Beyond the realm of specific linguistic phenomena, a philosophical debate has arisen about whether LLMs have any form of understanding or meaning (36). Some have indeed argued that text-only LLMs are fundamentally incapable of capturing meaning, under various construals of meaning (37, 38), while others have pointed to plausible ways in which text-only systems could be said to acquire meaning (39–42). We remain agnostic about whether LLMs truly capture meaning or only capture other properties that correlate with it; what we believe is clear is that meaning-sensitive tasks do not come naturally to systems trained solely on textual input, such that we can expect LLMs to encounter difficulty in handling these tasks.

C.4.2 Inability to modify text that has already been produced

As we mentioned in the introduction, Section 8 of (1) discusses a way in which LLMs are limited by the autoregressive nature of how they interact with language: LLMs produce text one word at a time. They are not explicitly trained to plan far ahead, and they are unable to alter text once they have produced it. These facts sometimes cause inaccuracies in model performance because LLMs will sometimes make mistakes attributable to an inability to plan ahead and cannot later correct that error because they cannot change their previously-produced text. An example of this issue is in Figure S12. (43) refer to this issue as self-delusion: models treat the text that they have previously produced as part of the input from which they should make future predictions, so errors in their previous output propagate forward into later outputs.

C.5 Phenomena relating to the training distribution

When evaluating any system that learns, it is important to consider what the system learns from (44–48). A wealth of papers have therefore highlighted ways in which language model performance is influenced by the model's training data. Some have shown ways in which the choice of dataset plays a role, both with respect to the type of data (e.g., Internet text vs. child-directed speech: (49)) and with respect to the way in which the dataset is curated or filtered (50, 51). On a more fine-grained level, several papers have connected model behavior to specific (pre-)training examples that have contributed to that behavior (52–58). Finally, several abstract abilities of language models have been traced to properties of their training data; such capacities include cross-lingual abilities (59) and in-context learning (60). Below, we highlight two particular properties of LLMs that connect directly to the training distribution.

Arithmetic puzzle

Prompt:
If a pen and a pencil together cost $2.50, and the pen costs $2 less than the pencil, how much does the pen cost? First give your answer, and then explain your reasoning.

GPT-4 output:
The pen costs $1.25.

Explanation:
Let's assume the cost of the pencil is x dollars.
The cost of the pen would then be $x - 2$ dollars.

According to the problem, the pen and pencil together cost $2.50:
$x + (x - 2) = 2.50$
$2x - 2 = 2.50$
$2x = 4.50$
$x = 2.25$

So the pencil costs $2.25, and the pen, which costs $2 less, would be:
$2.25 - 2 = 1.25$
The pen costs $1.25.

Prompt:
If a pen and a pencil together cost $2.50, and the pen costs $2 less than the pencil, how much does the pen cost? First explain your reasoning, and then give your answer.

GPT-4 output:
To solve this problem, we can use a system of equations.

Let P represent the cost of the pen and C represent the cost of the pencil.

1) The first equation comes from the statement "a pen and a pencil together cost $2.50":
$P + C = 2.50$

2) The second equation comes from the statement "the pen costs $2 less than the pencil":
$P = C - 2$

First, we'll solve for one variable in terms of the other using one of the equations. The second equation already gives us P in terms of C, so we can use it to substitute into the first equation:
$P + C = 2.50$
$(C - 2) + C = 2.50$
$2C - 2 = 2.50$
$2C = 4.50$
$C = 2.25$

Now we know the cost of the pencil, C, is $2.25. To find the cost of the pen, P, we can use the second equation $P = C - 2$:
$P = 2.25 - 2$
$P = 0.25$

So, the pen costs $0.25.

Figure S12: When given an arithmetic puzzle, GPT-4 got the wrong answer when it was prompted to first provide its answer and then its reasoning (top), but it got the right answer when it was prompted to provide the reasoning first and then the answer (bottom). These examples were produced by the online version of GPT-4 on September 17, 2023.

C.5.1 Societal biases and spurious correlations

Language models are trained to capture the statistical properties of their training sets. At times, these statistics can have undesirable effects on model behavior. First, neural network models can absorb societal biases that are encoded in natural textual distributions (61–63), leading them to behave in bigoted ways such as producing toxic, prejudiced text (64, 65). Second, neural networks are susceptible to using invalid heuristics that yield the correct answer most of the time in their training distribution but that are not valid strategies in the general case (66, 67). For example, when language models are given several sentences with sentiment labels and are then asked to label the sentiment of a new sentence, their answer is highly influenced by the labels of the examples they were shown: they are biased toward producing the label that is most common among the examples, relying too heavily on the invalid statistical cue of label frequency rather than the cues that truly indicate sentence sentiment (3). Both of these phenomena—societal biases and spurious correlations—are driven by properties of the distribution that a model is trained on, and the fact that models are so sensitive to such properties has driven calls to curate models' training data more carefully (68).

C.5.2 Idiosyncratic memorization

In the main paper, we have documented the fact that LLMs are biased toward sentences that have a high probability. It is important to note that, from the perspective of an LLM, the probability of a sentence is determined by the particular dataset that the LLM was trained on. As a result, if the dataset contains some sentences that are frequently repeated, the model is likely to memorize them—even if they are not important or high-probability sentences in the broader world.

(57) provide one example. They show that several language models trained on the Wikitext-103 dataset (69), when left to their own devices, have a tendency to generate sentences about a rowing competition called the Boat Race, such as the sentence in (2):

(2) The Boat Race is a side-by-side rowing competition between the University of Oxford (sometimes referred to as the "Dark Blues") and the University of Cambridge (sometimes referred to as the "Light Blues").

The reason for this tendency is that the training set is a collection of Wikipedia articles, and it happens to contain 159 articles about the Boat Race (one article for each year that the event has been held: "The Boat Race 1861", "The Boat Race 1862", etc.) There are several sentences that are repeated word-for-word across these articles, so it is no surprise that models have memorized these frequent sentences, even though they are not particularly notable sentences outside the context of this dataset.

C.6 Phenomena relating to the architecture

There are several perspectives in cognitive science which emphasize that, in order to understand an information-processing system, it is important to consider the computational resources that the system has at its disposal. Because the human brain is a type of neural network (albeit a type that is very different from the networks used in LLMs), many of these perspectives are couched in discussions of how our understanding of higher cognition should be informed by the fact that the mind emerges from the brain, as opposed to being explained solely using symbolic theories that are not rooted in neural networks; for an overview of perspectives on this topic, see (70). For our purposes, the perspectives that are particularly noteworthy are those saying that, even when the goal is to understand higher-level cognitive functions, it remains important to view the mind through the lens of the neural architecture that it emerges from—where this architecture is sometimes taken to be the primary consideration for cognitive theories (71) and is sometimes taken to be something that should be unified with higher-level symbolic analyses (72, 73). Outside of debates about the role of neural networks, our perspective overlaps with resource rationality (74)—the idea that cognition should be analyzed as the rational use of limited resources to accomplish a task. This view is a modification of more traditional rational analysis (16), which focuses on the task that one aims to perform but not on the resources that one has access to; since we consider not just training task but also model architecture, our approach is closer to a resource-rational view than a more traditional rational view.

Many lines of research in AI can be understood as ways to understand and improve models based on the limitations that their architectures impose. For instance, new modeling developments are typically motivated by insights about the limitations of previous architectures: RNNs (75), unlike preceding systems, were able to process variable-length sequential data; LSTMs (76) overcame the vanishing gradient problem inherent in non-gated RNNs; Transformers (77) afforded more efficient training than LSTMs; and current research now seeks to overcome the context-length limitations of standard Transformers by enabling them to efficiently handle longer contexts (78, 79). Below we specify two embers of autoregression that can be attributed to model architecture.

C.6.1 Sensitivity to tokenization and other aspects of input formatting

All models that take in data need to have that data represented in some way. The choice of input format can have important implications for model behavior. For instance, (80) provided an influential early criticism of neural-network modeling, focused on the model of (81), which was trained to take in an English verb and produce its past-tense form. Some of their points identify ways in which the model's input format makes it fundamentally incapable of handling certain cases; for instance, it does not distinguish between different words that are pronounced the same, meaning that it cannot capture cases where two identically-pronounced words have different past tenses (e.g., *break* and *brake* have the same pronunciation, yet their past tenses are pronounced differently: *broke* vs. *braked*).

In more recent models, the way in which the input is formatted continues to be important. Current models of language typically use subword tokenization schemes (82) in which each word is encoded as a sequence of one or more tokens, each of which can be made of one or more contiguous letters. One consequence of this choice is that some languages are more expensive to process than others due to differences in how straightforwardly this approach can handle a language's writing system (83); another consequence is that models can be brittle to perturbations (e.g., typos) that cause a word to be broken into unfamiliar token sequences, whereas approaches that do not rely on subword tokens can be more robust to such perturbations (84). The fact that our tasks were challenging for GPT models may also relate to their tokenization scheme: though GPT-3.5 and GPT-4 do encode the spellings of their tokens (see Section L), this information may be less readily accessible to them than it would be to models in which characters are fully listed in the input, which would be a source of difficulty in tasks that involve character-level manipulations (e.g., rot-13 decoding).

C.6.2 Limited compositionality and systematicity

(85) and (86) both presented influential arguments for why neural networks, due to the way they operate, are likely to struggle with the related phenomena of compositionality (combining familiar structures in novel ways) and systematicity (handling related stimuli in unified ways). Compositionality and systematicity have continued to be important targets of neural network evaluation (87–89). Although models have shown some impressive advances in evaluations of compositionality (e.g., 90), fully compositional behavior continues to elude them in at least some settings (91), motivating proposals that aim to enhance neural networks in ways that support symbolic abstractions (e.g., 92, 93).

We do not claim that neural networks are fundamentally incapable of compositional or systematic behavior. On the contrary, theoretical work has proven that neural networks are capable of capturing these properties (94). Our claims are not therefore about what is possible; rather, they are about what is straightforward. Even though neural networks *can* behave compositionally and systematically, it is not straightforward for them to do so, so it can be expected that their handling of these phenomena will encounter some difficulties.

C.7 Beneficial embers of autoregression

In discussing the ways in which models are shaped by the problem they solve, we have mostly discussed negative consequences, because we believe that the positive consequences are already well-recognized; indeed, they are some of the reasons why certain tasks, training distributions, and architectures have become prevalent.

First, the next-word prediction objective provides a major benefit by virtue of its generality (95): almost any task can be cast in terms of next-word prediction, such that training a model on this task will also give

it training on a wide range of other tasks (96). For instance, predicting what words will come after the phrase *In summary* requires a model to summarize the preceding text, giving it experience in the task of summarization.

Second, Internet text is useful as a training distribution because it gives models exposure to a wide range of types of examples. As a result, very few types of input are out-of-distribution for models, mitigating the ways in which neural networks are brittle to out-of-distribution examples.

Finally, the use of a neural network as the model architecture has several positive attributes compared to a symbolic approach, including similarity-based generalization, multiple constraint satisfaction, graceful degradation, and content-addressable memory (71), as well as the ability to learn efficiently from naturalistic data (97).

D Effect of chain-of-thought prompting

All of our experiments in the main paper used basic prompting, in which models are simply asked to provide the response to a query. For many of our tasks, it is likely that model performance could be increased by more advanced prompting techniques, such as chain-of-thought prompting (98) and step-by-step prompting (99); for instance, (98)'s chain-of-thought approach substantially improved performance on the task of last-letter concatenation, which is similar to our acronym task. Similarly, it is also likely that performance on our tasks will improve as models are further scaled up. However, it would not invalidate our conclusions if there are conditions in which LLM performance is greater than what we have observed. We do not claim to be highlighting fundamental incapabilities of LLMs but rather are claiming that some tasks and examples are harder for LLMs than others. Therefore, the existence of *any* setting that yields the predicted performance differences supports our claims, even if other settings exist where LLM performance is at ceiling for both conditions being compared.

As a first step toward extending our results to other prompting techniques, we used chain-of-thought prompting and step-by-step prompting to evaluate GPT-4 on shift ciphers (see Appendices G.7 through G.11 for details on the prompts). The results are in Figure S13 and show that, at least when decoding, these prompting techniques can indeed substantially increase performance (for encoding—shown in the bottom right of Figure S13—they appear to hurt performance). Nonetheless, it is worth noting that the basic effects we have identified still hold (see Appendix I.2.4 for significance tests): First, even with more advanced prompting, GPT-4 shows similar patterns of difficulty on different shift levels—e.g., showing a spike in accuracy at a shift of 13, and showing greater accuracy for rot-1 and rot-3 than rot-2 (Figure S13, top). Second, GPT-4 still performs better on high-probability sentences than low-probability ones, particularly when decoding (Figure S13, bottom). Thus, while more advanced prompting techniques improve overall accuracy, they do not erase the ways in which models are influenced by their autoregressive roots.

E Expanded results

In this section, we expand upon some of the results in the main paper by presenting additional examples, experimental conditions, and discussion.

E.1 Additional examples for experiments presented in the main paper

Several figures below provide more examples for experiments presented in the main paper. To save space, these figures only show results for GPT-3.5 and GPT-4; see the figures in the main paper for results with Llama 3, Claude 3, and Gemini 1.0.

1. Figure S14 provides examples of the five Pig Latin variants discussed in the main text.
2. Figure S15 provides examples of the two types of acronyms discussed in the main text (first-letter acronyms and second-letter acronyms).
3. Figure S16 provides examples of the two linear functions discussed in the main text.

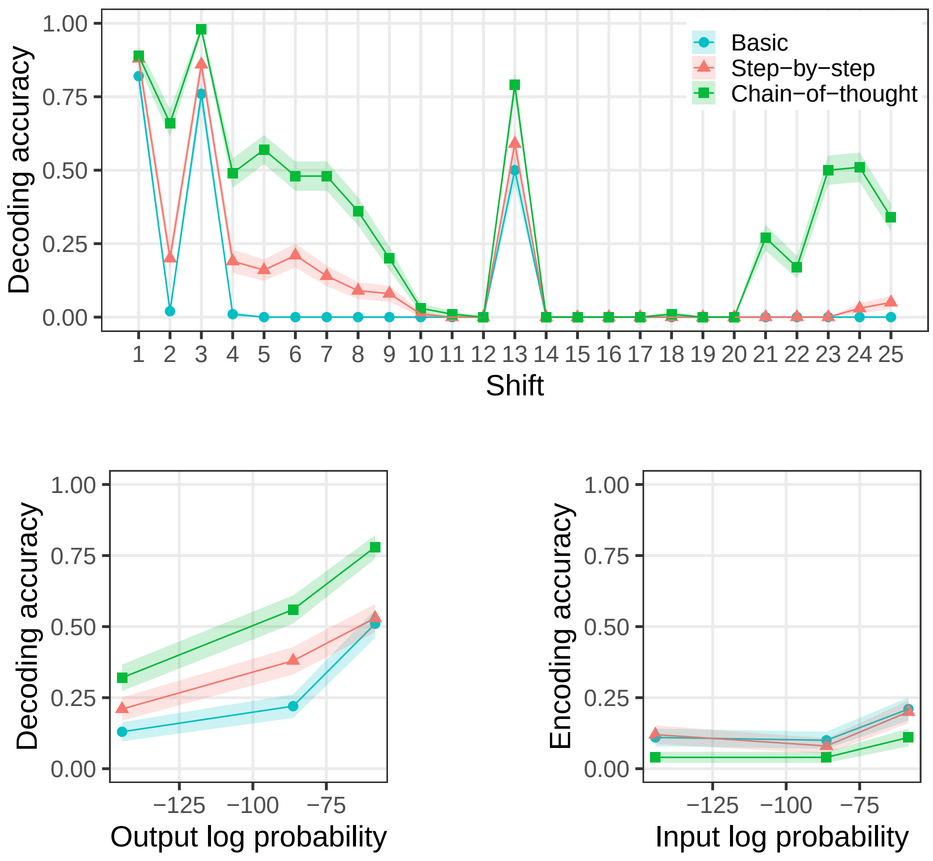

Figure S13: Comparing three prompting styles (basic, step-by-step, and chain-of-thought) on processing shift ciphers. In all plots, the intervals around the lines show one standard error. All results shown here are with GPT-4. **Top:** Accuracy on decoding shift-cipher-encrypted text with different shifts. **Bottom left:** Influence of output probability when decoding. **Bottom right:** Influence of input probability when encoding.

4. Figure S17 provides examples of the two sorting tasks discussed in the main text (sorting into alphabetical or reverse alphabetical order).

5. Figure S18 provides examples of the effects of output probability for the tasks of shift ciphers, reversal, and Pig Latin.

6. Figure S19 provides examples of the effect of output probability for the article swapping task.

7. Figure S20 provides examples of the effect of output probability for the acronym task.

E.2 Expanded counting experiments

In the main paper, we investigated how output probability influenced models' performance at counting lists of words. Here we add an analyses of their performance at counting lists of characters and of the effects of input probability.

We presented models with a list of either characters or words. The list length varied from 1 to 100; we showed 30 examples of each length and then computed an accuracy for each number based on those 30 examples. Figure S21 plots model accuracy as a function of the magnitude of the number being counted to (middle) or the log frequency of the number being counted to (bottom), where the frequency was estimated based on how often each number appears in the first 1 billion words in the C4 corpus (11). The curve is

often smoother when based on frequency than when based on magnitude, a point made more formally by seeing that accuracy has a higher Spearman rank correlation with frequency than with magnitude in every case except Llama 3 counting words (Table S2). Finally, in logistic regressions, output log probability had a highly significant effect for GPT-3.5, GPT-4, and Claude 3, and Gemini 1.0 ($p < 10^{-15}$ for counting words and for counting characters, for all four of these models; see Appendix I.7). It did not have a significant effect for Llama 3 counting words ($p = 0.12$) but did have a significant effect for Llama 3 counting characters ($p < 10^{-15}$). One possible explanation for why Llama 3 does not show clear output probability effects for counting words like the others does is that its counting accuracy is generally low, especially for the larger numbers where frequency and magnitude dissociate, leaving the Llama 3 results little room to display an effect of output probability that is distinct from the effect of magnitude. Output magnitude also had a significant effect in many cases; see Appendix I.7 for more details.

As another way to disentangle magnitude from frequency, we did a test that controlled for magnitude but varied frequency. We identified all numbers n from 1 to 100 that were at least twice as frequent as $n-2$, $n-1$, $n+1$, or $n+2$. Such numbers were labeled high-probability, while their neighbors ($n-2$, $n-1$, $n+1$, and $n+2$) were labeled low-probability. We then evaluated models on 100 examples for each high-probability number and 25 examples for each low-probability number; average magnitude was thus matched because the mean of $n-2$, $n-1$, $n+1$, and $n+2$ is n. Llama 3 counting words had an accuracy near 0% on this dataset,

Encoding with Pig Latin variants

Task: Convert the following English sentence into Pig Latin (using a variant of Pig Latin that is specified in the prompt): "`in times of crisis, art should be pragmatic.`"

Pig Latin with *-way*
Correct: `inway imestay ofway isiscray, artway ouldshay ebay agmaticpray.`
GPT-4: `inway imestay ofway isiscray, artway shouldway ebay ragmaticpay.`

Pig Latin with *-ay*
Correct: `inay imestay ofay isiscray, artay ouldshay ebay agmaticpray.`
GPT-4: `inay imestay ofay isiscray, artay shoulday ebay ragmaticpay.`

Pig Latin with *-yay*
Correct: `inyay imestay ofyay isiscray, artyay ouldshay ebay agmaticpray.`
GPT-4: `inay imestay ofay isiscray, artay shouldyay ebay ragmaticpay.`

Pig Latin with *-hay*
Correct: `inhay imestay ofhay isiscray, arthay ouldshay ebay agmaticpray.`
GPT-4: `inay imestay ofhay isiscray, artay houldsay ebay ragmaticpay.`

Pig Latin with *-say*
Correct: `insay imestay ofsay isiscray, artsay ouldshay ebay agmaticpray.`
GPT-4: `inay imestay ofsay isiscray, artsay shouldsay ebay ragmaticpray.`

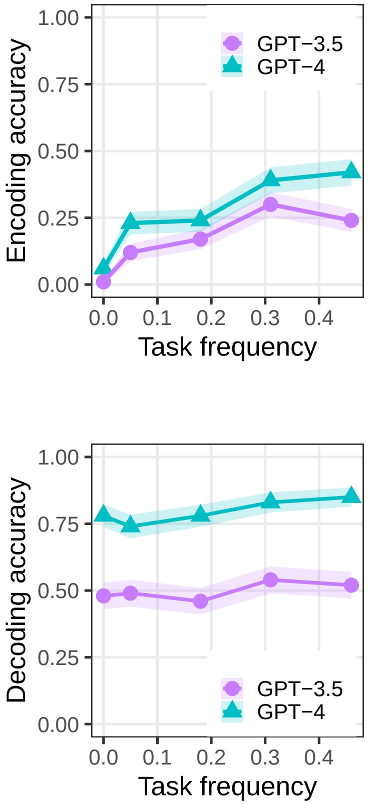

Figure S14: **Left:** An English sentence and its equivalent in five Pig Latin variants, along with GPT-4's predictions for the Pig Latin sentences in these variants. The variants are listed from most to least frequent in the Pile corpus. Errors are highlighted in red; cases where GPT-4 produced the correct variant-specific form of a vowel-initial word are highlighted in green (note that the variants only differ in how they handle vowel-initial words). **Right:** Model accuracy on these variants when encoding (top) or decoding (bottom), as a function of the variant's frequency. The intervals around the lines show one standard error. The plots repeat some information from Figure 8 from the main paper.

Acronyms: Task probability

Common task: First-letter acronym. Combine the **first** letters of the words in the sequence.

Input: `penance aplenty rooster trample impasse envious subtext`
Correct: `PARTIES`
✓ **GPT-4:** `PARTIES`

Input: `unbound newness cranium likable emerald abalone reissue`
Correct: `UNCLEAR`
✗ **GPT-4:** `UNCLERA`

Uncommon task: Second-letter acronym. Combine the **second** letters of the words in the sequence.

Input: `aplenty maestro precept strayed figment megaton ascetic`
Correct: `PARTIES`
✗ **GPT-4:** `PLEEGET`

Input: `quattro ennoble scissor fluency regency hawkish pricked`
Correct: `UNCLEAR`
✗ **GPT-4:** `UENLUEAW`

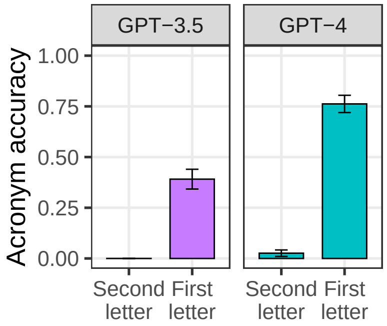

Figure S15: **Left:** Examples of GPT-4 predictions on the acronym tasks. **Right:** Model accuracy at acronym formation (this plot repeats some acronym results from Figure 9 in the main paper). Error bars show one standard error.

Linear functions: Task probability

Common function: $f(x) = (9/5)x + 32$

Input: `64`
Correct: `147.2`
✓ **GPT-4:** `147.2`

Input: `577`
Correct: `1070.6`
✗ **GPT-4:** `1069.6`

Rare function: $f(x) = (7/5)x + 31$

Input: `64`
Correct: `120.6`
✗ **GPT-4:** `89.8`

Input: `577`
Correct: `838.8`
✗ **GPT-4:** `805.4`

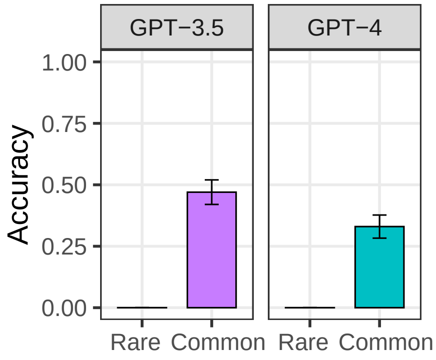

Figure S16: **Left:** Examples of GPT-4 predictions on the linear functions. **Right:** Model accuracy at linear functions (this plot repeats some linear function results from Figure 9 in the main paper). Error bars show one standard error.

but for all other cases accuracy was much higher for the high-probability outputs than the low-probability ones (Figure S21, top right), and all of these differences were statistically significant (see Appendix I.7).

In order to vary the input probability when having models count characters, we used letters as high-probability characters and emojis as low-probability characters. To modulate input probability when counting words, we estimated word probability using GPT-2 (as we did for acronyms) and then sorted the vocabulary by probability. We selected 450 words from the higher-probability half and 450 words from the

Sorting: Task probability

Common task: Sort in **alphabetical** order.

Input: `big, evergreen, flagrant, donkey, illegal, clean, exalted, normative, bountiful, archaic, insufferable, better, fair`

Correct: `archaic, better, big, bountiful, clean, donkey, evergreen, exalted, fair, flagrant, illegal, insufferable, normative`

✓ **GPT-4:** `archaic, better, big, bountiful, clean, donkey, evergreen, exalted, fair, flagrant, illegal, insufferable, normative`

Uncommon task: Sort in **reverse alphabetical** order.

Input: `big, evergreen, flagrant, donkey, illegal, clean, exalted, normative, bountiful, archaic, insufferable, better, fair`

Correct: `normative, insufferable, illegal, flagrant, fair, exalted, evergreen, donkey, clean, bountiful, big, better, archaic`

✗ **GPT-4:** `normative, insufferable, illegal, evergreen, exalted, flagrant, fair, donkey, clean, bountiful, big, better, archaic`

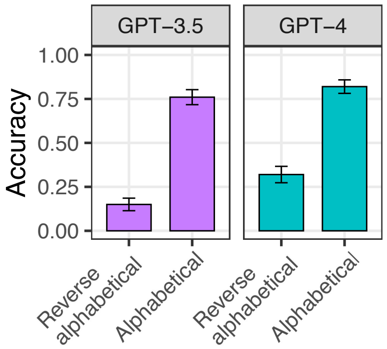

Figure S17: **Left:** Examples of GPT-4 predictions on sorting tasks. **Right:** Model accuracy at sorting (this plot repeats some sorting results from Figure 9 in the main paper). Error bars show one standard error.

lower-probability half to serve as our two types of words. The results of these comparisons are in Figure S22. Many of the differences are statistically significant (see Appendix I.7), though in some cases they are significant in the opposite direction from what is expected (with higher input probability being associated with lower accuracy). It is unclear why these cases show effects in the direction that is opposite from what we expected.

(100) and (101) also found that GPT-4 struggled with counting. Our experiment takes this result further by connecting it to probability, which is in turn connected to our broader teleological analysis of why models behave as they do.

E.3 Expanded analysis of shift cipher errors

In the main paper, we briefly discussed some of the types of errors that the models displayed when solving shift ciphers in two subsections entitled "Analysis of shift cipher errors." Here we provide more detail and quantitative analyses. For these analyses, we only consider GPT-3.5, GPT-4, and Claude 3, since the other models had accuracies of 0% on the relevant datasets.

Targeted examples: We tested models on examples that were more targeted, such that our hypotheses not only predict poor performance but also make predictions about *which* incorrect answers models will produce. To create such examples, we started with our high-probability sentences and then manually changed one word in each to a new word that, in the context of the sentence, was still grammatical but was unlikely. The new word always had a Levenshtein edit distance (102) of at most 2 from the original word (counting a single insertion, deletion, or substitution as 1 edit). For example, given (3a), we produced (3b). We evaluated GPT-3.5, GPT-4, and Claude 3 on decrypting rot-13 text where the correct outputs were our modified sentences.

(3) a. Well, if they don't come, so be it.
    b. Well, if they don't code, so be it.

Under the hypothesis that models favor high-probability answers, we predict that they will "regularize" their outputs for these cases by producing the high-probability sentence that is similar to the correct answer

Shift cipher: Output probability

Rot-13: Example of high-probability output

Input: `Gur erfbyhgvba bs pbasyvpgf unf gb or onfrq ba gur gehgu.`
Correct: `The resolution of conflicts has to be based on the truth.`
✓ **GPT-4:** `The resolution of conflicts has to be based on the truth.`

Rot-13: example of medium-probability output

Input: `Nyy rfpnyngvba bs shapgvbaf unq gb or onfrq ba uvf sbphf.`
Correct: `All escalation of functions had to be based on his focus.`
✗ **GPT-4:** `All escalation of conflicts had to be based on his proofs.`

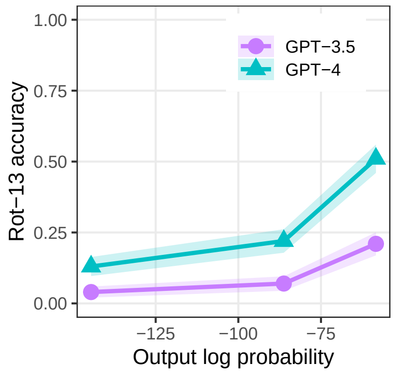

Reversal: Output probability

Reversal: Example of medium-probability output

Input: `paintings. the with pleased totally not was he True,`
Correct: `True, he was not totally pleased with the paintings.`
✓ **GPT-4:** `True, he was not totally pleased with the paintings.`

Reversal: example of low-probability output

Input: `paintings. the with pleased he totally was not True,`
Correct: `True, not was totally he pleased with the paintings.`
✗ **GPT-4:** `True, not was he totally pleased with the paintings.`

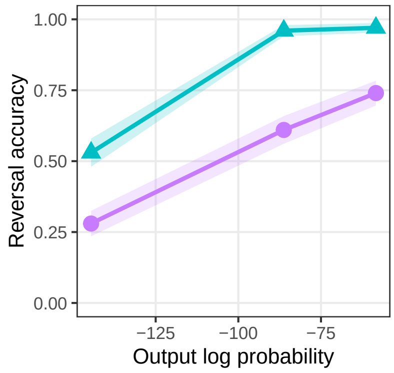

Pig Latin: Output probability

Pig Latin: Example of high-probability output

Input: `utbay isthay imetay, erethay aymay alsoay ebay anotheray easonray.`
Correct: `but this time, there may also be another reason.`
✓ **GPT-4:** `but this time, there may also be another reason.`

Pig Latin: example of medium-probability output

Input: `etyay ithway ustray, erethay aymay evenay ebay anotheray eformray.`
Correct: `yet with rust, there may even be another reform.`
✗ **GPT-4:** `yet with trust, there may even be another reform.`

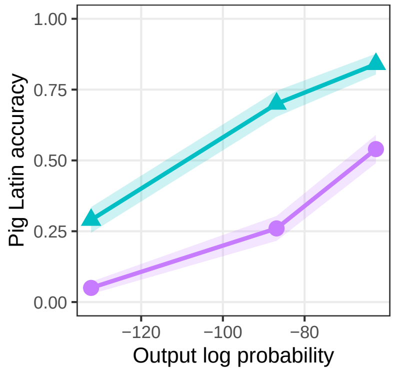

Figure S18: The effect of output probability on model accuracy across three tasks. In all plots, the intervals around the lines show one standard error. The plots repeat parts of Figure 4 in the main paper.

Article swapping: Output probability

Article swapping: Example of high-probability output

Input: `She distributed items the around room the and we tried them on.`
Correct: `She distributed the items around the room and we tried them on.`
✓ **GPT-4:** `She distributed the items around the room and we tried them on.`

Article swapping: example of medium-probability output

Input: `Hal distributed props the across park the and he takes more in.`
Correct: `Hal distributed the props across the park and he takes more in.`
✗ **GPT-4:** `Hal distributed the props across the park and he takes in more.`

Article swapping: example of low-probability output

Input: `Hal more he the props and the across distributed takes park in.`
Correct: `Hal more the he props the and across distributed takes park in.`
✗ **GPT-4:** `Hal takes the more props and distributed them across the park in.`

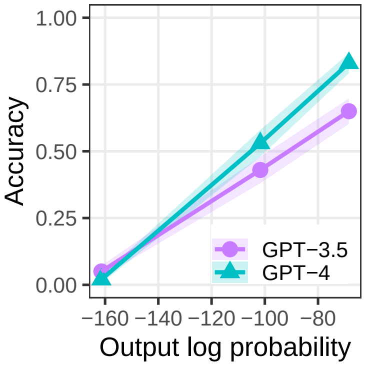

Figure S19: **Left:** Examples of the article swapping task. **Right:** The effect of output probability on model accuracy on this task. The intervals around the lines show one standard error. Due to collinearity, we cannot tell with certainty whether the accuracy differences are driven by output probability or input probability, but based on the results of an additional set of experiments we have concluded that the results are most likely driven by output probability; see Section I.5.2. The plot repeats parts of Figure 4 in the main paper.

Acronyms: Output probability

Example of high-probability output

Input: `salient tribune outlast parades panache emerald dweller`
Correct: `STOPPED`
✓ **GPT-4:** `STOPPED`

Example of low-probability output

Input: `gremlin affixed manatee empress lasagna impasse naively`
Correct: `GAMELIN`
✗ **GPT-4:** `GAMENIL`

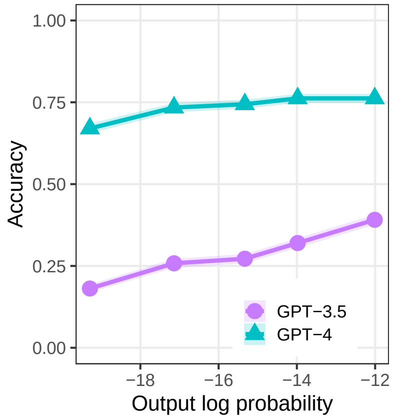

Figure S20: Effect of output probability on acronym performance. The target output was always a real word that appeared in the CMU Pronouncing Dictionary, but the commonness of that word varied; the horizontal axis shows different output probability levels (e.g., *STOPPED* is a common word, and *GAMELIN* is a rare word). The plot repeats parts of Figure 4 in the main paper.

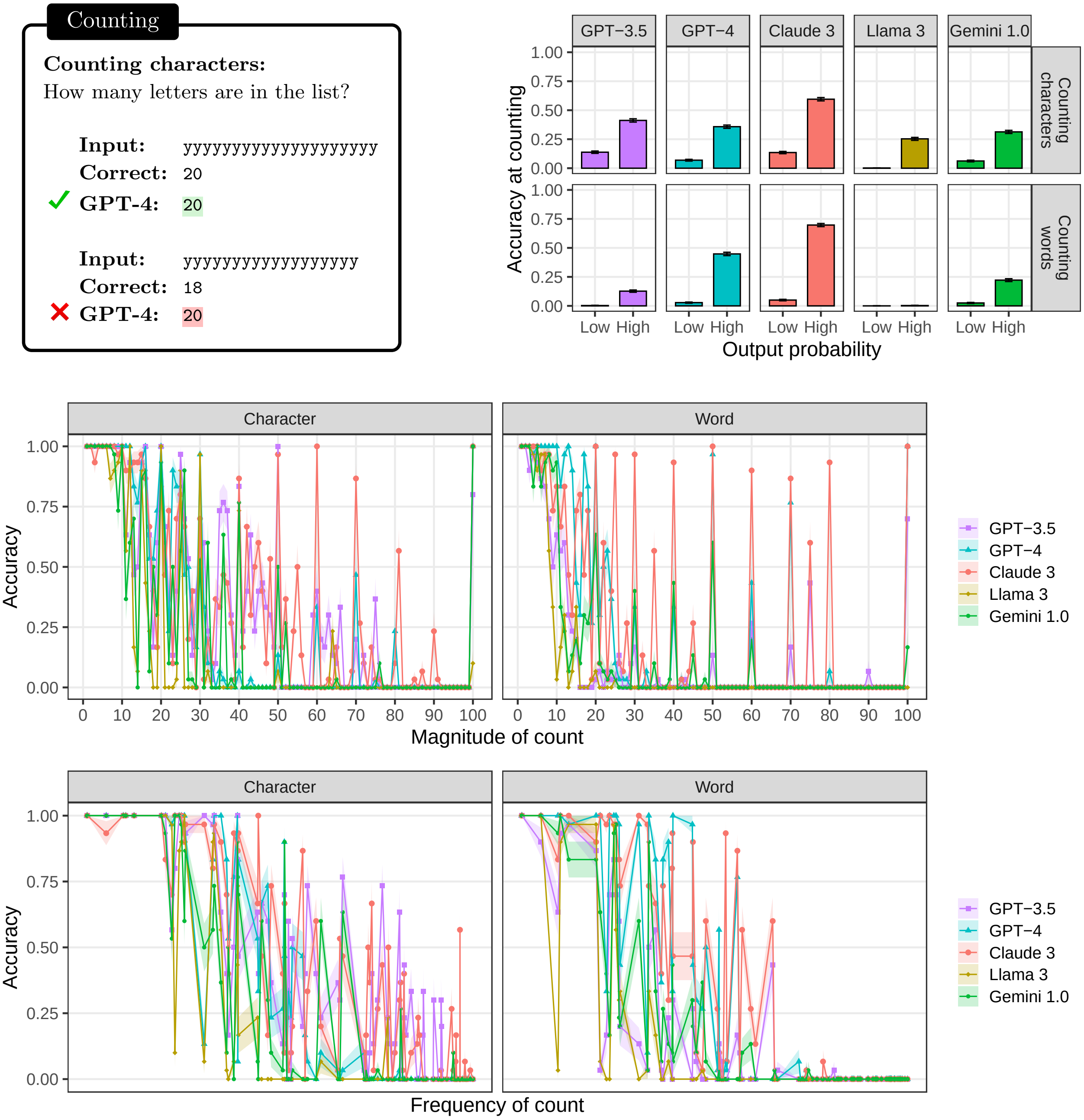

Figure S21: **Top left:** Examples of stimuli for counting characters; the stimuli for counting words are similar except that the characters being counted are all the same within an example, but the words being counted are all different. **Top right:** Counting accuracy in settings with low-probability and high-probability outputs. In this context, the output is a number (the number of elements in the list), and *output probability* refers to the frequency of that number in an Internet corpus. Error bars show one standard error. **Middle and bottom:** Counting performance by the magnitude (middle) or frequency (bottom) of the number being counted to. We use the C4 corpus (11) to estimate each number's frequency. The intervals around the line show one standard error. The "Word" plot in the middle row repeats Figure 5 from the main paper.

	|cor(Accuracy, Magnitude)|	|cor(Accuracy, Frequency)|
GPT-3.5: Counting words	0.57	0.74
GPT-3.5: Counting characters	0.81	0.84
GPT-4: Counting words	0.70	0.84
GPT-4: Counting characters	0.76	0.88
Llama 3: Counting words	0.64	0.62
Llama 3: Counting characters	0.64	0.73
Claude 3: Counting words	0.66	0.85
Claude 3: Counting characters	0.73	0.83
Gemini 1.0: Counting words	0.71	0.81
Gemini 1.0: Counting characters	0.71	0.78

Table S2: Investigating which features correlate with LLM accuracy at counting. The two features considered are the magnitude of the number being counted to and the frequency in a corpus of the number being counted to. The table shows the absolute value of the Spearman rank correlation coefficients between accuracy and each of these features. In all cases but one, accuracy correlates more strongly with frequency than with magnitude; the exception is Llama 3 counting words.

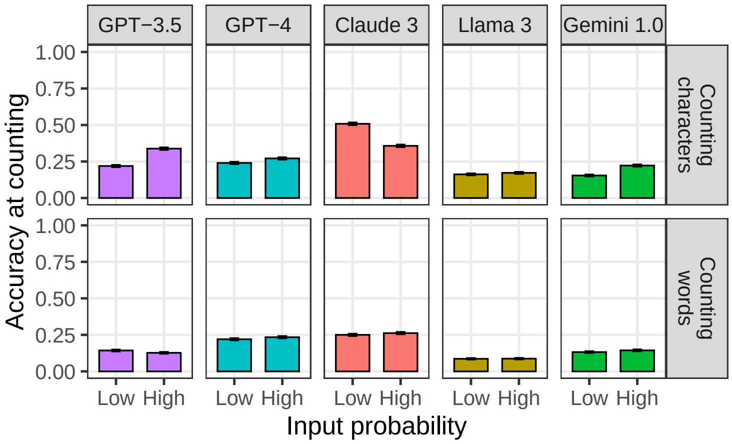

Figure S22: Accuracy on counting when input probability is modulated. Error bars show one standard error.

(e.g., (3a)) rather than the correct answer (e.g., (3b)). We evaluated both whether the full output sentence matched the correct or regularized answer, and also whether just the critical word matched the correct word or the regularized version. Under both metrics, both GPT models produced the regularized version more often than the correct version (Figure S23); Claude 3 also produced some regularized outputs, but it produced the correct answer more often than the regularized one. Here are some cases where GPT-4 produced an incorrect regularized answer:

(4) a. **Correct output:** Because of this, their names were chanted for security reasons.
b. **GPT-4 output:** Because of this, their names were changed for security reasons.

(5) a. **Correct output:** Are they now building a bridge of their owl?
b. **GPT-4 output:** Are they now building a bridge of their own?

(6) a. **Correct output:** We are very careful about the voices we want to address, so we need to collaborate with otters for sustainable impact.
b. **GPT-4 output:** We are very careful about the voices we want to address, so we need to collaborate with others for sustainable impact.

(7) a. **Correct output:** This is a great achievement for us and a very beige responsibility.
b. **GPT-4 output:** This is a great achievement for us and a very big responsibility.

A related type of regularization was shown in (103), whose "resisting correction" results showed that models, when asked to repeat back a sentence containing a typo, often fixed the typo. Our findings show that such modifications can occur even when the sentence could be correct as written but is simply low-probability.

These results provide further evidence for the conclusion that LLMs are sensitive to the probability of the outputs that they produce. There are certainly contexts where such regularization is desirable, such as when it leads a model to fix typos. Therefore, we included this experiment not as a way to showcase model failure but rather as a way to highlight the types of strategies used by LLMs—strategies that may have desirable effects in some contexts but undesirable effects in others.

Further analysis of model errors: For decoded rot-13 text, model predictions generally adhered fairly closely to the correct answer. Even when models made mistakes, they were not egregious, as in the following examples from rot-13 decoding:

(8) a. **Correct answer:** What are your hopes for the independent filmmaking scene in Indonesia?
b. **GPT-4 output:** What are your hopes for the impending filmmaking scene in Indianapolis?

As the overall lower accuracy would suggest, model errors in rarer shifts deviate more from the correct answer. Consider rot-10: GPT-4's responses in this case have an average Levenshtein distance of 68.4, vs. 3.4 for rot-13. In such cases where the model output diverges dramatically from the correct answer, an interesting pattern emerges where the outputs are often recognizable sayings or quotations. For instance, among the rot-10 predictions are a popular motivational saying (9)[1] and (a modified version of) a sentence famous for using all letters of the alphabet (10):

(9) a. **Correct answer:** We are very careful about the voices we want to address, so we need to collaborate with others for sustainable impact.
b. **GPT-4 output:** We are what we repeatedly do. Excellence, then, is not an act, but a habit.

(10) a. **Correct answer:** She never regretted her passion for the artistic craft, nor did she waver in her tireless dedication.
b. **GPT-4 output:** The quick brown fox jumps over the lazy dog, but not the sheep in the background.

In another case, GPT-4 produced the start of a Shakespearean soliloquy (11). Clearly something is rot-10 in the state of Denmark:

[1]This saying originated from Will Durant but is often attributed to Aristotle.

Shift ciphers: Output regularization

Full sentence correct:

Input: `Gung'f bhe jnl bs gunaxvat naq jung jr onfr bhe jbex ba.`
Correct: `That's our way of thanking and what we base our work on.`
✓ **GPT-4:** `That's our way of thanking and what we base our work on.`

Full sentence regularized

Input: `V jvyy abg tvir hc rira sbe n zbahzrag.`
Correct: `I will not give up even for a monument.`
✗ **GPT-4:** `I will not give up even for a moment.`

Critical word correct (but with an error elsewhere)

Input: `Gur eryngvbafuvc orgjrra gurfr tebhcf vf bhe znvy punyyratr.`
Correct: `The relationship between these groups is our mail challenge.`
✗ **GPT-4:** `The relationships between these groups is our mail challenge.`

Critical word regularized (with an additional error elsewhere)

Input: `Gur fvghngvba ng gur havirefvgl yrirr vf dhvgr qrcybenoyr.`
Correct: `The situation at the university levee is quite deplorable`
✗ **GPT-4:** `The situation at the university level is quite dependable.`

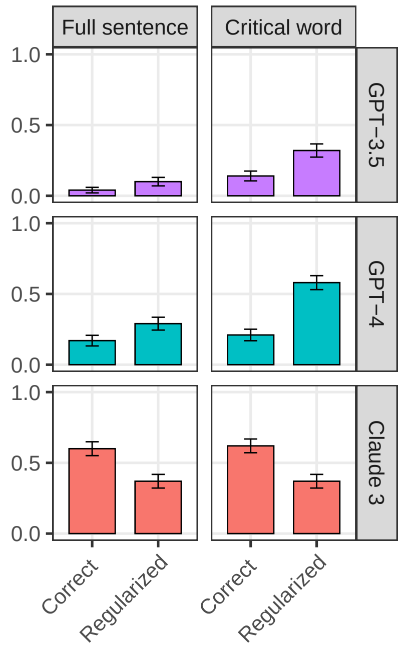

Figure S23: Shift cipher performance on locally modified sentences. **Left:** Examples of the stimuli. In each case, the correct answer contained one word (underlined) that was implausible in context but that was similar to a more plausible word; e.g., in the first example, the correct word *thanking* is similar to the incorrect but more plausible word *thinking*. We refer to the incorrect but plausible options as *regularized*. **Right:** The plots show how often model outputs matched the correct or regularized answer, either entirely (*Full sentence*) or at the *critical word* (e.g., *thanking* vs. *thinking*). Adjacent bars do not sum to 1.0 because models might produce an answer that is not the same as either option we consider. Error bars show one standard error.

(11) a. **Correct answer:** As a doctor of humanities, he was a university professor, founded a university and a newspaper, and won awards in journalism and literature.
b. **GPT-4 output:** To be or not to be, that is the question, whether tis nobler in the mind to suffer the slings and arrows of outrageous fortune.

This tendency to produce well-known sayings is consistent with the effect of output probability described in the main paper. The less well the model has learned a task, the more uncertainty it will have about the relationship between output and input, which is what defines the likelihood. It will therefore be more influenced by the prior, resulting in predictions that are not very closely connected to the input but that have a high probability in absolute terms, such as well-known quotations.

Lack of separation between instructions and input: One other type of model error is worth discussing as an illustration of how models rely on probability. Specifically, the models' outputs often include words relating to ciphers even when the correct answer does not. The following examples are all from GPT-4 decoding rot-2 text:

(12) a. **Correct answer:** This decision was influenced by the political climate of the time.
b. **GPT-4 output:** This decryption was engineered by the programming language of the time.

(13) a. **Correct answer:** We could suggest things, but we didn't know how a city council worked.
b. **GPT-4 output:** We could seriously damage, but we didn't know how a code breaker worked.

(14) a. **Correct answer:** The definition of a donor was also narrowed, making it difficult for foreign donors to fit this requirement.
b. **GPT-4 output:** The decryption of a cipher was also designed, making it easier for other people to get their messages to fit this protocol.

The previous sections showed ways in which models are sensitive to the unconditional probability of their output. However, these new examples cannot be explained in that way; e.g., in (12), the model-produced word *decryption* has a much lower unconditional probability than the correct word *decision*. Instead, these examples show models relying on the input but in an incorrect way: the input includes directions that talk about a cipher (using words such as *cipher* and *decode*), and this makes models likely to produce related words in their output, even when the output should not be influenced by the instructions in this way.

E.4 Expanded discussion of the asymmetry between input probability and output probability

Now that we have run experiments modulating output probability and input probability, we have the opportunity to compare these two factors to see if input probability is indeed less influential, as we have hypothesized. One immediate piece of evidence supporting this conclusion is that almost all of our output probability experiments yielded significant effects—often with large effect sizes—whereas only a few input probability conditions yielded significant effects.

In addition to noting which experiments produced significant effects, we can also look for an input/output asymmetry by considering model performance in the high-probability rot-13 conditions. GPT-4 scores 0.51 for decoding rot-13 in the high-probability setting but only 0.21 for encoding rot-13 in the high-probability setting; GPT-3.5 scores 0.21 for decoding and 0.02 for encoding; Llama 3 scores 0.05 for decoding and 0.00 for encoding; Claude 3 scores 0.98 for decoding and 0.70 for encoding; and Gemini 1.0 scores 0.00 in both decoding and encoding. Thus, all models except Gemini 1.0 perform substantially better on decoding than encoding (and Gemini 1.0's lack of a difference is likely due to floor effects, since its accuracy is 0.00 in both relevant conditions). This asymmetry between decoding performance and encoding performance supports the conclusion that output probability is more influential than input probability. The argument for why these results support this conclusion is as follows:

- In a shift cipher, encoded text is usually a very low-probability string of letters.[2] Therefore, encoding text involves turning a high-probability sequence into a low-probability one, while decoding text involves the opposite. (The fact that we want to ensure that the English text is high-probability is why, in the previous paragraph, we considered only the high-probability conditions within the encoding and decoding tasks).
- We have ample evidence to believe that low probability is a source of difficulty for LLMs. Therefore, we have one task (encoding) where the input is easy to process yet the output is hard to process, and one task (decoding) where the opposite is true.
- Whichever "side" (input vs. output) the LLM is more sensitive to is the one that the LLM's overall performance would pattern with, since the variations in difficulty in the less-influential side would be overwhelmed by the variations in difficulty in the more-influential side.
- Since LLM performance follows the difficulty of the output rather than the difficulty of the input (i.e., higher accuracy for decoding than encoding), we have evidence that LLMs are more sensitive to output probability than input probability.

One potential concern with the previous argument is that it assumes that encoding and decoding have no inherent difference in complexity as tasks, such that any differences in performance can be attributed to the specific text rather than the task being performed. In the general case, it is true that a function and its inverse may not always be equally easy to compute; e.g., it is much harder to find the prime factors of a

[2]There are rare exceptions where shifting text produces other recognizable text. For instance, the rot-13 encoding of *vex* is its synonym *irk*, the rot-13 encoding of *abjurer* is *nowhere*, and fans of *2001: A Space Odyssey* have pointed out that the name of the computer HAL becomes IBM when given a shift of 1.

	Count in C4	GPT-3.5	GPT-4	Llama 3	Claude 3	Gemini 1.0
Alphabetical order	95,942	0.76	0.82	0.67	0.92	0.65
Reverse alphabetical order	629	0.15	0.32	0.18	0.50	0.04
Ascending order	21,562	0.66	0.82	0.57	1.00	0.70
Descending order	31,378	0.59	0.80	0.42	1.00	0.46

Table S3: Corpus counts and model accuracies for sorting tasks.

large number than it is to multiply together prime factors. However, rot-13 is a special case because it is its own inverse—that is, rot-13 encoding and rot-13 decoding are in fact the same function. It still remains possible that the standard ways to perform each task differ in difficulty: encoding for rot-n is traditionally performed by moving forward n positions in the alphabet, while decoding is traditionally performed by moving backward n positions. However, if anything, we would intuitively expect that moving forward would be easier than moving backward because the alphabet is much more often said in forward order than reverse order. Therefore, if this factor does play a role it would be in the direction of making encoding easier than decoding. In sum, we have no reason to believe that encoding is inherently a more complex task than decoding, justifying the conclusion that the difference in accuracy is driven by the properties of the input and output strings, not by differences in the mapping that must be performed.

In addition to rot-13, we have one other task that is its own inverse: reversal. Here too most models performed better on decoding than encoding: GPT-4 scored 0.97 for high-probability decoding but only 0.87 for high-probability encoding; GPT-3.5 scored 0.74 for high-probability decoding but 0.46 for high-probability encoding; Llama 3 scored 0.52 for high-probability decoding but 0.05 for high-probability encoding; and Gemini 1.0 scored 0.61 for high-probability decoding but 0.30 for high-probability encoding. The one exception to the trend was Claude 3, which scored 0.91 for high-probability decoding but 0.95 for high-probability encoding. Setting aside this one exception, the reversal results, like the rot-13 results, support our hypothesis that LLM accuracy is affected more strongly by output probability than input probability.

E.5 Expanded sorting results

In the main paper, we compared model performance on sorting a list into alphabetical order (which is used fairly commonly) to performance on sorting a list into reverse alphabetical order (which is much rarer). As predicted, models performed better on the more common ordering than the rarer one Although these two ways of sorting *words* show a marked difference in frequency, the two most prevalent ways of sorting *numbers*—in ascending order or in descending order—do not show such a large difference. Specifically, we found that descending order is mentioned approximately 1.5 times as often as ascending order in the C4 corpus, a much smaller ratio than the ratio of 150 found for word orderings. We found that, for all five models, there is less of a gap between ascending order and descending order than there is between alphabetical order and reverse alphabetical order, potentially due to the more similar corpus frequencies that these orderings have (Table S3).

One aspect of these ascending vs. descending results that is potentially puzzling is that all models (except for Claude 3, whose performance was saturated at 1.00) performed better on ascending order than descending order (albeit often not by much), even though our corpus analysis found that descending order was mentioned more often. A potential explanation is that our corpus analysis was based on the frequencies of the phrases *in ascending order* and *in descending order*. It is possible that ascending order is in fact used more often but just not explicitly mentioned as much—that is, perhaps lists that are in descending order are usually accompanied by the phrase *in descending order* while lists in ascending order often appear without the phrase *in descending order*.

Task	Sample size
Article swapping	100
Reversal	100
Counting	See caption
Acronyms	1000
Linear functions	100
Multiplication	100
Sorting	100
Keyboard cipher	100
Shift cipher	100
Pig Latin	100
Birthdays	100

Table S4: Sample sizes for our datasets. Each number shows the sample size for one condition, corresponding to a single point in each of our line plots or a single bar in each of our bar plots; e.g., for reversal, we had 3 conditions, yielding a total of 300 examples. For counting, there were different sample sizes for different settings. The results in the top right of Figure S21 use a sample size of 1200 per bar; the plots in the middle and bottom of that figure use a sample size of 30 per point; and the results in Figure S22 use a sample size of 3000 per bar.

F Dataset details

F.1 Sample sizes

Table S4 specifies how many examples there were in each dataset that we created for evaluating models.

F.2 Shift cipher sentences

On June 16, 2023, we downloaded the 50 most recent new articles from Global Voices[3] (spanning from June 2, 2023, to June 16, 2023), to minimize the chances that models would have seen these particular sentences—though it would not cause problems for our conclusions if they had because we intended for these sentences to be high-probability for the models. We selected Global Voices because its content has a permissive license that allows sharing and modification, with attribution.[4] A full list of the articles we used is at `https://github.com/tommccoy1/embers-of-autoregression/blob/main/global_voices_articles.md`. We then sentence-tokenized these articles using the NLTK sentence tokenizer (104). For each sentence, we used GPT-2 (96) to find the maximum perplexity of any token in that sentence and ranked sentences in order of increasing maximum perplexity; the goal was to find sentences in which no individual words were overly surprising. We then selected the first 100 sentences in this list that met the following criteria:

- Did not include proper names (except for very common ones such as the names of countries)
- Did not discuss violence, death, or sex.
- Did not have grammatical errors.

The resulting 100 sentences served as the high-probability sentences for being the output of shift cipher decryption or the input for shift cipher encryption.

To produce medium-probability sentences, we started with the high-probability sentences and then modified each one in a way that reduced its probability. Specifically, we tokenized the sentence using the RoBERTa-large tokenizer and then masked one token at a time, from left to right. For each masked token, we sampled a replacement token from RoBERTa-large (105), where the replacement token had to meet the following criteria:

[3] `https://globalvoices.org/`

[4] `https://globalvoices.org/about/global-voices-attribution-policy/`

- Must be within the top 95% of the probability mass
- Must not be within the top 90% of the probability mass
- Must be the same number of characters as the token being replaced

The first two criteria were intended to find replacements that were low-probability but not so low-probability as to be ungrammatical. The last criterion served to preserve the length of the sentence. If there were no tokens that fit all criteria, the token was left unchanged. Once a new token was sampled, the sentence was changed to incorporate it before the next token's replacement was sampled. The full sentence was required to have the same number of subword tokens as the original, measured using the tokenizer that is used by both GPT-3.5 and GPT-4.[5] This procedure did not always produce grammatical sentences; to ensure grammaticality, for each sentence, we produced 20 candidates (ranked by perplexity) and manually chose the highest-perplexity one that was grammatical. In a few cases, we had to generate more than 20 candidates or had to put together parts of multiple candidates.

To create each low-probability sentence, we started with a medium-probability sentence and then shuffled all of its words except the first word and last word. For this purpose, words were defined as whitespace-delimited units. The first word and last word were left in place to avoid unusual capitalization or punctuation.

To create the targeted sentences which differ from the high-probability ones by just one or two characters, we started with the high-probability sentences and then manually replaced one word in each one with another word. The new word had to have a Levenshtein edit distance (102) of at most 2 from the original word (where one edit is defined as a single-character insertion, deletion, or substitution). The new word was selected to be one that was grammatical yet unlikely in the given context. In the codebase, these sentences are referred to as *adversarial*.

The prompts used for rot-13 (Appendices G.1 and G.2) included one in-context example that clarified two potential points of confusion:

- It indicated that capitalization was retained in the output.
- It indicated that non-letter characters were to be left unchanged.

In all conditions, there were 100 examples. Table S5 shows some statistics for these examples that were used in our regression tests. The tokens were counted using the tokenizer used by GPT-3.5 and GPT-4. The log probabilities were estimated as the log probability assigned by GPT-2 to the sentences.

F.3 Reversal sentences and keyboard cipher sentences

For the reversal task and keyboard cipher task, we used the same sentences as for shift ciphers (Appendix F.2). As with the shift ciphers, there were 100 sentences per condition.

F.4 Pig Latin sentences

The Pig Latin sentences were generated with the same basic technique as the rot-13 sentences (Appendix F.2). However, Pig Latin is more constrained than rot-13 because Pig Latin is mainly a spoken phenomenon, so when it is written down there can be some ambiguity due to the ways in which English spelling does not match English pronunciation. E.g., should the Pig Latin for *hour* be *ouray* or *ourhay* or *houray*? Therefore, we excluded any sentences with words that created any such ambiguities. This included abbreviations, numbers, and words (such as *hour*) for which the initial consonant cluster in writing does not match well with the initial consonant cluster in speech—such mismatches were automatically flagged using the CMU Pronouncing Dictionary[6] and then manually checked. Thus, to generate Pig Latin sentences, we followed the same procedure as for rot-13 but discarded any examples that failed to meet these criteria, and sampled new examples until we had the requisite 100 examples per condition. All inputs and outputs were lowercased to avoid ambiguity about how to deal with capitalization (e.g., in Pig Latin, should *Latin* become *atinLay* or *Atinlay* or *atinlay*?). There were 100 examples per condition.

[5] The tokenizer is `cl100k_base` from the `tiktoken` package: `https://github.com/openai/tiktoken`

[6] `http://www.speech.cs.cmu.edu/cgi-bin/cmudict`

	Input chars.	Output chars.	Input tokens	Output tokens	Input logprob	Output logprob
Rot-13 decryption: High-probability	98.7	98.7	46.4	19.7	-302.6	-58.4
Rot-13 decryption: Medium-probability	98.7	98.7	46.8	19.7	-304.5	-86.3
Rot-13 decryption: Low-probability	98.7	98.7	46.8	19.7	-306.9	-144.5
Pig Latin decryption: High-probability	118.3	88.1	40.8	17.6	-256.3	-62.5
Pig Latin decryption: Medium-probability	118.3	88.1	40.8	17.6	-256.6	-86.9
Pig Latin decryption: Low-probability	118.3	88.1	40.8	17.6	-258.2	-132.3
Reversal decryption: High-probability	98.7	98.7	20.0	19.7	-140.6	-58.4
Reversal decryption: Medium-probability	98.7	98.7	20.1	19.7	-149.7	-86.3
Reversal decryption: Low-probability	98.7	98.7	20.1	19.7	-148.4	-144.5

Table S5: Average dataset statistics for some of the datasets discussed in Appendix F. For the encryption versions of the decryption tasks, the numbers are the same, but the input and output values are swapped. For the decryption datasets, the intent was to vary output log probability yet keep all other factors similar; for encryption, the goal was the opposite.

The prompts for the Pig Latin tasks (Appendices G.18 and G.19) include an in-context example that clears up several points of confusion:

- It illustrates how to handle punctuation.
- It shows not to break words on apostrophes.
- It illustrates the handling of both consonant-initial and vowel-initial words (a distinction that is also described in words in the prompt).

F.5 Article swapping sentences

The article swapping sentences were generated with the same basic technique as the rot-13 sentences (Appendix F.2), but with additional constraints added. First, each sentence had to contain at least one article (*a*, *an*, or *the*), since the task becomes trivial if there are no articles to be moved. Second, the sentence cannot start or end with an article because that could create issues when attempting to swap (e.g., if we are swapping each article with the previous word, and if the sentence starts with an article, then that initial article will not have anything to swap with). There were 100 examples per probability level.

F.6 Acronyms

Our initial word list was created by extracting all seven-letter words in the CMU Pronouncing Dictionary that were two tokens long under the tokenizer used by GPT-3.5 and GPT-4; the token count had to be two both when the word was preceded by a space and when it was not. This word list had two versions—one in all lowercase letters and one in all capital letters. The lists differed because the number of tokens is often different between lowercase and uppercase versions of a word. Each of these word lists was ordered from highest to lowest probability, where the probability in question was the probability that GPT-2 assigned to the word in the context *The word is "WORD"*—chosen to provide a context in which any word should fit reasonably well, and using quotation marks to clearly indicate the word's boundaries.

Each word list was then split into bins by probability. Certain words had to be excluded because no acronyms could be formed from them (e.g., due to a lack of words starting with X, no output could contain an X). After removing such words, there were 8203 lowercase words and 2244 all-caps words. These were split into bins as shown in Table S6; there was some space left between bins to make them more distinct, and we also omitted the words at the end of each list since their low probability under GPT-2 might reflect that something is unusual about them.

When varying the input probability, the output words were fixed across conditions—i.e., spelling the same word but from input words of differing probabilities. When comparing first-letter and second-letter acronyms, the same output words were used in the two conditions. In all comparisons (varying output probability, varying input probability, and comparing first-letter vs. second-letter acronyms), each word in the nth example had the same split point as the corresponding word in the nth example in all other conditions, where the *split point* is the index of the break in between the two tokens within the word; we controlled for this factor in case different split points lead to different difficulty levels. Within these constraints, examples were generated by first sampling an output word and then, for each letter in that word, sampling an input word that would yield that letter. We generated 1000 examples per condition.

To determine the log probabilities of the output words (for purposes of plotting accuracy vs. log probability, or for purposes of statistical tests), we used the log probability that GPT-2 assigned to the whole sequence *The word is "WORD"* minus the log probability that it assigned to *The word is "*; this gives the probability of the word plus the closing quotation mark conditioned on *The word is "*. We included the closing quotation mark because some effects of the word's probability might manifest in the following quotation mark (as the indicator that the word has ended). To compute the log probability of the input, we used this same approach to get the log probability of each input word, and we then summed the log probabilities of all the input words to yield the log probability of the whole input.

	Start	End
Lowercase bin 1	0	999
Lowercase bin 2	1500	2499
Lowercase bin 3	3000	3999
Lowercase bin 4	4500	5499
Lowercase bin 5	6000	6999
Capital bin 1	0	399
Capital bin 2	440	839
Capital bin 3	880	1279
Capital bin 4	1320	1719
Capital bin 5	1760	2159

Table S6: Acronym vocabulary binning. Each bin is inclusive of both its starting point and endpoint.

F.7 Counting

In the case of counting characters, we used lowercase letters as the common characters and emojis as the rare characters. In the case of counting words, we extracted all words from the CMU Pronouncing Dictionary that fit the following criteria:

- Must be 1, 2, or 3 tokens long.
- Must have the same number of tokens whether preceded by a space or not.
- Must appear at least 20 times in the Wikitext-103 corpus (69).

We then sorted all these words by the probability assigned to them by GPT-2 in the context *The word is "WORD"*, as described in Appendix F.6. Then, we split the resulting words into the highest-probability 8000 words and the rest (this divided the list roughly in half, as there were about 16,000 words meeting our criteria). To create our candidate high-probability words, we took the 150 highest-probability words for each token count of 1, 2, or 3, after manually excluding profanity and words that could conceivably be considered not words (e.g., because they are names); since we were asking models to count words, we wanted to avoid an interpretation where some "words" were ignored on the grounds that they were not in fact words. We used the same procedure to create our list of rare words, except that in this case we used the 150 lowest-probability words of each token length. This gave us a list of 450 high-probability words and 450 low-probability words.

For each integer from 1 to 100, we created 30 examples in each condition. To create a character example for count n, we uniformly sampled a character from the possible candidates and then repeated it n times (that is, within each character example, all characters were the same). To create a word example, we sampled n words without replacement; the words were matched for token patterns across common/rare conditions (e.g., if the "common words" example had a word with 3 tokens of length 2, 5, and 7, the "rare words" example would also have a word in that position with the same token pattern). We sampled words without replacement so that, within each word sequence, there were no duplicated words, to avoid any ambiguity about whether we intended for the count to include duplicates or not.

The frequency of each number was estimated by counting its occurrences in the first one billion words of the C4 corpus (11). In the experiment where we compared common and rare numbers, a number n was categorized as common if it occurred at least twice as many times as each of $n-2$, $n-1$, $n+1$, and $n+2$; this yielded twelve common numbers (20, 30, 40, 45, 50, 60, 70, 75, 80, 90, 95, 100). We then used $n-2$, $n-1$, $n+1$, and $n+2$ as rare numbers. We generated 100 examples for each common number and 25 examples for each rare number; this setup guaranteed that the common-number and rare-number conditions were matched for average magnitude of the counts.

F.8 Sorting

For sorting both words and numbers, the input was a list of length 10 to 20 inclusive; the list could not contain repeats. Each example was generated by first selecting a length (by uniform sampling) and then randomly selecting (without replacement) list elements from the set of candidates. For sorting words, the candidates were the 450 common words used in the counting task (Appendix F.7). For sorting numbers, the candidates were the integers from 1 to 10,000 inclusive. We generated 100 examples for each condition.

F.9 Three-digit multiplication

We generated 100 multiplication problems in which both numbers being multiplied were three-digit integers. The two numbers were sampled uniformly from the range [100,999]. The same set of 100 problems was used across all methods of formatting the text.

F.10 Linear functions

We generated 100 examples for all conditions. For the initial test, in which we compared $(9/5)x + 32$ to $(7/5)x + 31$ using the same set of inputs, we sampled 100 input values uniformly from the integers 0 to 999 inclusive. For the test in which we instead held the outputs constant, we sampled 100 output values from the integers 0 to 999 inclusive and then, for each function, used the integer input value—if there was one that satisfied our constraints—which yielded that output value after the function had been applied and the output had been rounded to the nearest integer. If either function did not have a suitable integer input value for a given output, we rejected that output and sampled another one. For the out-of-distribution tests, we added two additional constraints:

- The first number that was sampled (i.e., the Celsius input in the initial version of the test, or the Fahrenheit output in the inverse version) had to be in the range 501 to 999, inclusive.
- Neither number could be evenly divisible by 10.

We chose these constraints based on a corpus analysis of the C4 dataset (11) in which we aimed to determine the types of temperatures that are frequently present with conversions in an Internet corpus. In this analysis, we identified all sentences that contained both the word "fahrenheit" preceded by a number and the word "celsius" preceded by a number, or the characters "°f" preceded by a number and the characters "°c" preceded by a number, and assumed that the two numbers were a Fahrenheit and Celsius temperate being presented as conversions of each other. (We ignored case when searching for these strings). Of these 10,000 occurrences, 99.8% are excluded by applying our two constraints when the first constraint is applied to the Celsius temperature, and 99.7% are excluded by applying the constraints when the first constraint is applied to the Fahrenheit temperature. Thus, by enforcing these constraints, we can yield only temperatures of a type that tend to occur very rarely with a conversion in natural Internet text, reducing—but certainly not removing—the risk that models might have seen and copied it.

F.11 Birthdays

We first extracted all name/birth date pairs from the WikiBio dataset (106), restricting the set to names containing exactly two words and containing only the 26 basic letters of the Roman alphabet. We then counted how many times each name occurred in the first 100 million lines (approximately 3.6 billion words) of the C4 corpus (11). Using this frequency information, we created four sets of names. The first set contained names mentioned 2,000 times or more in our sample of C4; the second set contained names mentioned between 200 and 1,999 times inclusive; the third set contained names mentioned between 20 and 199 times inclusive; and the fourth set contained names mentioned between 5 and 19 times inclusive. We only included names that were unambiguous on Wikipedia (i.e., when the name is searched for, Wikipedia goes directly to the person's page, without redirecting to a disambiguation page, and the top of the resulting page does not include a link to a disambiguation page); we checked for ambiguity by manually searching for each candidate name on Wikipedia until we had gathered sufficiently many unambiguous ones. We also verified that, for each person we included, Wikipedia included that person's birthday, and the birthday listed

by Wikipedia matched the one listed in the WikiBio dataset. Each set contains 100 names, and we matched the birth dates across sets (i.e., the nth names across all sets have the same birth dates; e.g., in each of the four sets, the eighteenth person was born on September 27, 1972).

G Prompts

In this section, we show one complete example of a prompt used for each task in our experiments. Within each experimental condition, the prompts had the same task description across all examples, with the only variation coming from the input for the function to be performed. In cases where an in-context example was provided, it was also held constant across all examples within each experimental condition. In some cases, the prompts shown in the following sections had slight variations across sub-experimental conditions, when differences in task variation necessitated it—e.g., the shift cipher prompts always indicated which specific shift cipher was being used (rot-1, rot-2, etc.), while the examples below (Appendices G.1 and G.2) show only rot-13 for illustration.

G.1 Shift ciphers: Encryption

Rot-13 is a cipher in which each letter is shifted 13 positions forward in the alphabet. For example, here is a message and its corresponding version in rot-13:
Original text: "Stay here!"
Rot-13 text: "Fgnl urer!"

Here is another message. Encode this message in rot-13:
Original text: "To this day, we continue to follow these principles."
Rot-13 text:

G.2 Shift ciphers: Decryption

Rot-13 is a cipher in which each letter is shifted 13 positions forward in the alphabet. For example, here is a message written in rot-13 along with the original text that it was created from:
Rot-13 text: "Fgnl urer!"
Original text: "Stay here!"

Here is another message in rot-13. Decode this message to produce the original text:
Rot-13 text: "Gb guvf qnl, jr pbagvahr gb sbyybj gurfr cevapvcyrf."
Original text:

G.3 Shift ciphers: Encryption (0-shot within few-shot experiments)

Rot-13 is a cipher in which each letter is shifted 13 positions forward in the alphabet. Encode the following message into rot-13.
Original text: "The success of this new transport route will provide landlocked Central Asia with access to the sea and global markets and help turn Turkey into a major energy transit hub."
Rot-13 text:

G.4 Shift ciphers: Encryption (5-shot within few-shot experiments)

Rot-13 is a cipher in which each letter is shifted 13 positions forward in the alphabet. Below are some messages and their corresponding versions in rot-13. For the last example, only the original text is provided; encode that text into rot-13.

Original text: "Discussions also revolved around security, energy, and economic development for the benefit of citizens."

Rot-13 text: “Qvfphffvbaf nyfb eribyirq nebhaq frphevgl, raretl, naq rpbabzvp qrirybczrag sbe gur orarsvg bs pvgvmraf.”

Original text: “That’s our way of thinking and what we base our work on.”
Rot-13 text: “Gung’f bhe jnl bs guvaxvat naq jung jr onfr bhe jbex ba.”

Original text: “Soon after, he built academic and literary careers.”
Rot-13 text: “Fbba nsgre, ur ohvyg npnqrzvp naq yvgrenel pnerref.”

Original text: “No longer just a gentleman’s agreement, the co-management framework is a validation of the work of stakeholders both within and beyond the boundaries of parks.”
Rot-13 text: “Ab ybatre whfg n tragyrzna’f nterrzrag, gur pb-znantrzrag senzrjbex vf n inyvqngvba bs gur jbex bs fgnxrubyqref obgu jvguva naq orlbaq gur obhaqnevrf bs cnexf.”

Original text: “In 2022, the total trade turnover reached a historic high of USD 12.33 billion.”
Rot-13 text: “Va 2022, gur gbgny genqr gheabire ernpurq n uvfgbevp uvtu bs HFQ 12.33 ovyyvba.”

Original text: “The success of this new transport route will provide landlocked Central Asia with access to the sea and global markets and help turn Turkey into a major energy transit hub.”
Rot-13 text:

G.5 Shift ciphers: Decryption (0-shot within few-shot experiments)

Rot-13 is a cipher in which each letter is shifted 13 positions forward in the alphabet. Below is a message in rot-13. Provide the original text that this encoded message was created from.
Rot-13 text: “Gur fhpprff bs guvf arj genafcbeg ebhgr jvyy cebivqr ynaqybpxrq Prageny Nfvn jvgu npprff gb gur frn naq tybony znexrgf naq uryc ghea Ghexrl vagb n znwbe raretl genafvg uho.”
Original text:

G.6 Shift ciphers: Decryption (5-shot within few-shot experiments)

Rot-13 is a cipher in which each letter is shifted 13 positions forward in the alphabet. Below are some messages in rot-13 along with the original text that they were created from. The last example is missing its original text; provide the original text for that example.

Rot-13 text: “Qvfphffvbaf nyfb eribyirq nebhaq frphevgl, raretl, naq rpbabzvp qrirybczrag sbe gur orarsvg bs pvgvmraf.”
Original text: “Discussions also revolved around security, energy, and economic development for the benefit of citizens.”

Rot-13 text: “Gung’f bhe jnl bs guvaxvat naq jung jr onfr bhe jbex ba.”
Original text: “That’s our way of thinking and what we base our work on.”

Rot-13 text: “Fbba nsgre, ur ohvyg npnqrzvp naq yvgrenel pnerref.”
Original text: “Soon after, he built academic and literary careers.”

Rot-13 text: “Ab ybatre whfg n tragyrzna’f nterrzrag, gur pb-znantrzrag senzrjbex vf n inyvqngvba bs gur jbex bs fgnxrubyqref obgu jvguva naq orlbaq gur obhaqnevrf bs cnexf.”
Original text: “No longer just a gentleman’s agreement, the co-management framework is a validation of the work of stakeholders both within and beyond the boundaries of parks.”

Rot-13 text: “Va 2022, gur gbgny genqr gheabire ernpurq n uvfgbevp uvtu bs HFQ 12.33 ovyyvba.”
Original text: Ïn 2022, the total trade turnover reached a historic high of USD 12.33 billion.”

Rot-13 text: “Gur fhpprff bs guvf arj genafcbeg ebhgr jvyy cebivqr ynaqybpxrq Prageny Nfvn jvgu npprff gb gur frn naq tybony znexrgf naq uryc ghea Ghexrl vagb n znwbe raretl genafvg uho.”
Original text:

G.7 Comments on step-by-step and chain-of-thought prompts

We used an initial informal exploration of several options to select the specific step-by-step and chain-of-thought prompts that we used. For step-by-step prompting, we compared two approaches: adding the sentence *Make sure to think step by step* into the middle of the prompt, or adding the sentence *Let's think step by step* at the end of the prompt. We found the former to be more successful, so we used it for step-by-step prompting (Appendices G.8 and G.9). For chain-of-thought prompting, we tried the following options:

- Convert one word at a time (e.g., given *Fgnl urer!*, first encode *Fgnl* and then *urer!*)
- Convert one letter at a time, each on its own numbered line of the generation (there were three variants: (i) do not include spaces; (ii) leave a blank line for a space; or (iii) leave a numbered line for a space exactly as if it were a letter).
- Convert one word at a time, but within the word, break it down into letters.
- Move the entire sentence back one position in the alphabet at a time. E.g., given *Fgnl urer!*, first shift it to *Efmk tqdq!*, then *Delj spcp!*, etc.

We were expecting the last option to perform the best, but we instead found that one of the letter-based variants performed the best, so we used that (Appendices G.10 and G.11).

G.8 Shift ciphers step-by-step: Encryption

Rot-13 is a cipher in which each letter is shifted 13 positions forward in the alphabet. For example, here is a message and its corresponding version in rot-13:
Original text: "Stay here!"
Rot-13 text: "Fgnl urer!"

Here is another message. Encode this message in rot-13. Make sure to think step by step. On the last line, write the words "Rot-13 text:" followed by the encoded message:
Original text: "But this time, there may also be another reason."

G.9 Shift ciphers step-by-step: Decryption

Rot-13 is a cipher in which each letter is shifted 13 positions forward in the alphabet. For example, here is a message written in rot-13 along with the original text that it was created from:
Rot-13 text: "Fgnl urer!"
Original text: "Stay here!"

Here is another message in rot-13. Decode the message to produce the original text. Make sure to think step by step. On the last line, write the words "Original text:" followed by the decoded message:
Rot-13 text: "Ohg guvf gvzr, gurer znl nyfb or nabgure ernfba."

G.10 Shift ciphers chain-of-thought: Encryption

Rot-13 is a cipher in which each letter is shifted 13 positions forward in the alphabet. For example, here is a message to be encoded:
Original text: "Stay here!"

To encode this message, we shift each letter 13 positions forward:
1. S -> F
2. t -> g
3. a -> n
4. y -> l
5. ->
6. h -> u

7. e -> r
8. r -> e
9. e -> r
10. ! -> !

Therefore, the rot-13 text is: “Fgnl urer!”

Here is another message. Encode the message one letter at a time. On the last line, write the words “Rot-13 text:” followed by the encoded message:
Original text: “But this time, there may also be another reason.”

G.11 Shift ciphers chain-of-thought: Decryption

Rot-13 is a cipher in which each letter is shifted 13 positions forward in the alphabet. For example, here is a message written in rot-13:
Rot-13 text: “Fgnl urer!”

To decode this message, we shift each letter 13 positions backward:
1. F -> S
2. g -> t
3. n -> a
4. l -> y
5. ->
6. u -> h
7. r -> e
8. e -> r
9. r -> e
10. ! -> !

Therefore, the original text is: “Stay here!”

Here is another message in rot-13. Decode the message one letter at a time. On the last line, write the words “Original text:” followed by the decoded message:
Rot-13 text: “Ohg guvf gvzr, gurer znl nyfb or nabgure ernfba.”

G.12 Reversal: Encryption

Reverse the following sequence of words. Punctuation marks should be moved along with the words they are attached to; e.g., the reversed version of “Good morning, everyone!” would be “everyone! morning, Good”: “But this time, there may also be another reason.”

G.13 Reversal: Decryption

Reverse the following sequence of words. Punctuation marks should be moved along with the words that they are attached to; e.g., the reversed version of “everyone! morning, Good” would be “Good morning, everyone!”: “letter. sons, may another also be there with Yet”

G.14 Reversal: Encryption (0-shot, within few-shot experiments)

Below is a sequences of words. Provide the sequence that is created when you reverse this sequence. (When the sequence is being reversed, punctuation marks should be moved along with the words that they are attached to.)
Original text: “The success of this new transport route will provide landlocked Central Asia with access to the sea and global markets and help turn Turkey into a major energy transit hub.”
Reversed text:

G.15 Reversal: Encryption (5-shot, within few-shot experiments)

Below are some sequences of words; each sequence is followed by the reversed version of that sequence. (When a sequence is being reversed, punctuation marks are moved along with the words that they are attached to.) The last example is missing the reversed sequence; provide the reversed sequence for this example.
Original text: “Discussions also revolved around security, energy, and economic development for the benefit of citizens.”
Reversed text: “citizens. of benefit the for development economic and energy, security, around revolved also Discussions”

Original text: “That’s our way of thinking and what we base our work on.”
Reversed text: “on. work our base we what and thinking of way our That’s”

Original text: “Soon after, he built academic and literary careers.”
Reversed text: “careers. literary and academic built he after, Soon”

Original text: “No longer just a gentleman’s agreement, the co-management framework is a validation of the work of stakeholders both within and beyond the boundaries of parks.”
Reversed text: “parks. of boundaries the beyond and within both stakeholders of work the of validation a is framework co-management the agreement, gentleman’s a just longer No”

Original text: “In 2022, the total trade turnover reached a historic high of USD 12.33 billion.”
Reversed text: “billion. 12.33 USD of high historic a reached turnover trade total the 2022, In”

Original text: “The success of this new transport route will provide landlocked Central Asia with access to the sea and global markets and help turn Turkey into a major energy transit hub.”
Reversed text:

G.16 Reversal: Decryption (0-shot, within few-shot experiments)

Below is a sequence of words in reversed order. Provide the original sequence that this sequence was created from. (When the sequence was reversed, punctuation marks were moved along with the words that they were attached to.)
Reversed text: “hub. transit energy major a into Turkey turn help and markets global and sea the to access with Asia Central landlocked provide will route transport new this of success The”
Original text:

G.17 Reversal: Decryption (5-shot, within few-shot experiments)

Below are some sequences of words in reversed order; each sequence is followed by the original sequence that the reversed sequence was created from. (When each sequence was reversed, punctuation marks were moved along with the words that they were attached to.) The last example is missing the original sequence; provide the original sequence for this example.
Reversed text: “citizens. of benefit the for development economic and energy, security, around revolved also Discussions”
Original text: “Discussions also revolved around security, energy, and economic development for the benefit of citizens.”

Reversed text: “on. work our base we what and thinking of way our That’s”
Original text: “That’s our way of thinking and what we base our work on.”

Reversed text: “careers. literary and academic built he after, Soon”
Original text: “Soon after, he built academic and literary careers.”

Reversed text: “parks. of boundaries the beyond and within both stakeholders of work the of validation a is framework co-management the agreement, gentleman’s a just longer No”

Original text: “No longer just a gentleman’s agreement, the co-management framework is a validation of the work of stakeholders both within and beyond the boundaries of parks.”

Reversed text: “billion. 12.33 USD of high historic a reached turnover trade total the 2022, In”
Original text: “In 2022, the total trade turnover reached a historic high of USD 12.33 billion.”

Reversed text: “hub. transit energy major a into Turkey turn help and markets global and sea the to access with Asia Central landlocked provide will route transport new this of success The”
Original text:

G.18 Pig Latin: Encryption

There is a secret way of talking called Pig Latin in which you move the first consonant cluster of each word to the end of the word, and then add -ay to the end of the word. If the word starts with a vowel, you simply add -ay to the end without otherwise changing the word. For example, below is a sentence in English and its equivalent in Pig Latin:
English: “the frogs aren’t noisy.”
Pig Latin: “ethay ogsfray aren’tay oisynay.”

Write this sentence in Pig Latin:
English: “turkey almost exclusively relies on energy imports as its domestic output covers only three percent of its energy needs.”
Pig Latin:

G.19 Pig Latin: Decryption

There is a secret way of talking called Pig Latin in which you move the first consonant cluster of each word to the end of the word, and then add -ay to the end of the word. If the word starts with a vowel, you simply add -ay to the end without otherwise changing the word. For example, below is a sentence in Pig Latin and its equivalent in English:
Pig Latin: “ethay ogsfray aren’tay oisynay.”
English: “the frogs aren’t noisy.”

Convert this sentence from Pig Latin to English:
Pig Latin: “urkeytay almostay exclusivelyay eliesray onay energyay importsay asay itsay omesticday outputay overscay onlyay eethray ercentpay ofay itsay energyay eedsnay.”
English:

G.20 Article swapping: Decoding, swap with previous

Repeat the sentence listed as Input 2 below, but every time there is an article (“the”, “a”, or “an”), swap it with the previous word. For example, if the input were Input 1, you should reply with Output 1:
Input 1: “When we boarded train the, we saw strange a sight.”
Output 1: “When we boarded the train, we saw a strange sight.”

Input 2: “Well, it was not exactly planned from beginning the.”
Output 2:

G.21 Article swapping: Decoding, swap with next

Repeat the sentence listed as Input 2 below, but every time there is an article (“the”, “a”, or “an”), swap it with the following word. For example, if the input were Input 1, you should reply with Output 1:
Input 1: “When we the boarded train, we a saw strange sight.”
Output 1: “When we boarded the train, we saw a strange sight.”

Input 2: “Well, it was not exactly planned the from beginning.”
Output 2:

G.22 Article swapping: Encoding, swap with previous

Repeat the sentence listed as Input 2 below, but every time there is an article ("the", "a", or "an"), swap it with the previous word. For example, if the input were Input 1, you should reply with Output 1:
Input 1: "When we boarded the train, we saw a strange sight."
Output 1: "When we the boarded train, we a saw strange sight."

Input 2: "Well, it was not exactly planned from the beginning."
Output 2:

G.23 Article swapping: Encoding, swap with next

Repeat the sentence listed as Input 2 below, but every time there is an article ("the", "a", or "an"), swap it with the following word. For example, if the input were Input 1, you should reply with Output 1:
Input 1: "When we boarded the train, we saw a strange sight."
Output 1: "When we boarded train the, we saw strange a sight."

Input 2: "Well, it was not exactly planned from the beginning."
Output 2:

G.24 Acronyms

What sequence of letters is created when you combine the first letters of the words in the sequence "chevron outback nomadic ferrous entitle splurge surfeit"? Write your answer in capital letters, with no spaces or punctuation.

G.25 Counting: Words

How many words are in the following list? "stilted hyperbolic secret charlatan normative malevolent flippant lawless gullible flagrant albatross purposeful"

G.26 Counting: Letters

How many letters are in the following list? "zzzzzzzzzzzzzz"

G.27 Counting: Emojis

How many tortoise emojis are in the following list? "🐢🐢🐢🐢🐢🐢🐢🐢🐢🐢🐢🐢"

G.28 Counting: Words (5-shot, in few-shot learning experiments)

Below are some lists of words. After each list, we have stated how many words are present in that list. For the final list, we have not specified the word count. Respond by saying how many words are in that list.

List: "albatross fake ukulele now experience right shenanigans almost workable incandescent autopilot exalted fire antipathy feisty sordid special ethereal hallowed heinous revolution more pretentious tenacious mediocrity matrimony truth scoundrel criminal inclusive magic mischievous most sequester evasive inevitability confederate archaic caviar donkey majesty canary safe cobbler expediency smug unilaterally discredited unqualified stilted crocodile implacable lost lullaby deluge frugal counterproductive despicable cauldron overrated haphazard unbridled awesome deluded beautiful poppy flammable unsatisfactory deep good tabloid super ghastly unprovoked yes wrong immortality"
Number of words: 77

List: "tabloid just evil common hot nomenclature"
Number of words: 6

List: “edgy tempestuous strong parallel not imprecise epiphany silent weak indeterminate overpriced pungent shenanigans inclusive unilaterally potential criminal uninvited elite precocious transparent provocation they probably public hallowed charlatan bickering emergency universal disintegration fake magic hyphenated experience inaudible soft”
Number of words: 37

List: “iconoclastic renegade flammable challenge obnoxious insinuation disingenuous unprofitable unsuitable power popular newsworthy overpriced disarray incendiary unprovoked insipid first truth fiduciary out cheap soft creative cranky abysmal race man serenity clean happy lost monolith still demagogue implacable pretentious virtuous autopilot ethereal boomerang saffron wild disheveled flimsy foreign insincere archaic evil inclusive natural heinous antipathy provocation peace realist insubstantial made unsatisfactory weak unique hidden disintegration deft insubordination brainwashing human wondrous uninvited deluded majesty willful peddler full social unilaterally parallel experience tricked alternative anonymous good fire unimpressive cool grandiose flagrant exuberant insufferable audacious”
Number of words: 90

List: “indelible modesty boomerang hooligan indestructible culture selfless immorality dominion free notoriety shadowy scavenge iconoclastic strident impervious precocious and tricked antithesis excitable mercantile inclusive normative expedited right overpriced anonymous uncontrolled war unremarkable smart virtuous nimble autopilot wrong amicable forceful true magic justifiable peddler emptiness significant fire marsupial unprofessional evil inevitability weak yes kind objective subliminal obnoxious indecent cordial parallel unbreakable may challenge complicit our brainwashing hasty laudable cauldron flimsy uneventful serious illegal people epiphany malice all delusion unbridled charlatan wild live raven ethereal incompatibility violent tyrant nonchalant pancake symmetrical exasperated gaffe uncooperative now strong overrated lawless expediency green lullaby scoundrel”
Number of words: 99

List: “bandit realist indelible devious leniency discontinue significant obnoxious violent hyperbolic epiphany idealistic insipid”
Number of words:

G.29 Sorting words: Alphabetical order

Sort the following list of words in alphabetical order: “illogical, hard, scoundrel, over, sequester, irrefutable, lost, anathema, raucous, wondrous”

G.30 Sorting words: Reverse alphabetical order

Sort the following list of words in reverse alphabetical order: “illogical, hard, scoundrel, over, sequester, irrefutable, lost, anathema, raucous, wondrous”

G.31 Sorting numbers: Ascending order

Sort the following list of numbers in ascending order: “1292, 3475, 8774, 7076, 3695, 6441, 6298, 9762, 1916, 6988, 8084”

G.32 Sorting numbers: Descending order

Sort the following list of numbers in descending order: “1292, 3475, 8774, 7076, 3695, 6441, 6298, 9762, 1916, 6988, 8084”

G.33 Sorting words: Alphabetical order (0-shot, in few-shot learning experiments)

Below is a list of words. Sort the words into alphabetical order.
Original list: “illogical, hard, scoundrel, over, sequester, irrefutable, lost, anathema, raucous, wondrous”
Sorted list:

G.34 Sorting words: Alphabetical order (5-shot, in few-shot learning experiments)

Below are several list of words, each of which is followed by the same list but sorted into alphabetical order. The sorted version of the last list has not been provided. Sort this list.

Original list: “official, brainwashing, now, amicable, cheap, utopian, transparent, penguin, scavenge, challenge, people, fast, strong, gift, bandit, family, fabricate, workable”
Sorted list: “amicable, bandit, brainwashing, challenge, cheap, fabricate, family, fast, gift, now, official, penguin, people, scavenge, strong, transparent, utopian, workable”

Original list: “laudable, peddler, wrong, pure, shoddy, retribution, over, discontinue, friendly, shadowy, like, hard, malevolent”
Sorted list: “discontinue, friendly, hard, laudable, like, malevolent, over, peddler, pure, retribution, shadowy, shoddy, wrong”

Original list: “abysmal, about, implacable, indestructible, inquisitive, incessant, salamander, unscientific, gaffe, unqualified, organic, unhelpful, lullaby”
Sorted list: “about, abysmal, gaffe, implacable, incessant, indestructible, inquisitive, lullaby, organic, salamander, unhelpful, unqualified, unscientific”

Original list: “vanguard, green, eternally, acrobatic, race, dynamic, irrefutable, foreign, bandit, albatross, ungrateful, epiphany, special, good, legal, dominion, tepid, overrated, willful, anathema”
Sorted list: “acrobatic, albatross, anathema, bandit, dominion, dynamic, epiphany, eternally, foreign, good, green, irrefutable, legal, overrated, race, special, tepid, ungrateful, vanguard, willful”

Original list: “libel, invincible, unremarkable, friendly, albatross, stilted, farcical, unfriendly, dexterity, normal, rapid, our, culture, confederate, almost, purposeful, epistemic, enchantment, disorganized, exorbitant”
Sorted list: “albatross, almost, confederate, culture, dexterity, disorganized, enchantment, epistemic, exorbitant, farcical, friendly, invincible, libel, normal, our, purposeful, rapid, stilted, unfriendly, unremarkable”

Original list: “illogical, hard, scoundrel, over, sequester, irrefutable, lost, anathema, raucous, wondrous”
Sorted list:

G.35 Sorting words: Reverse alphabetical order (0-shot, in few-shot learning experiments)

Below is a list of words. Sort the words into reverse alphabetical order.
Original list: “illogical, hard, scoundrel, over, sequester, irrefutable, lost, anathema, raucous, wondrous”
Sorted list:

G.36 Sorting words: Reverse alphabetical order (5-shot, in few-shot learning experiments)

Below are several list of words, each of which is followed by the same list but sorted into reverse alphabetical order. The sorted version of the last list has not been provided. Sort this list.

Original list: “official, brainwashing, now, amicable, cheap, utopian, transparent, penguin, scavenge, challenge, people, fast, strong, gift, bandit, family, fabricate, workable”
Sorted list: “workable, utopian, transparent, strong, scavenge, people, penguin, official, now, gift, fast, family, fabricate, cheap, challenge, brainwashing, bandit, amicable”

Original list: “laudable, peddler, wrong, pure, shoddy, retribution, over, discontinue, friendly, shadowy, like, hard, malevolent”
Sorted list: “wrong, shoddy, shadowy, retribution, pure, peddler, over, malevolent, like, laudable, hard, friendly, discontinue”

Original list: “abysmal, about, implacable, indestructible, inquisitive, incessant, salamander, unscientific, gaffe, unqualified, organic, unhelpful, lullaby”
Sorted list: “unscientific, unqualified, unhelpful, salamander, organic, lullaby, inquisitive, indestructible, incessant, implacable, gaffe, abysmal, about”

Original list: “vanguard, green, eternally, acrobatic, race, dynamic, irrefutable, foreign, bandit, albatross, ungrateful, epiphany, special, good, legal, dominion, tepid, overrated, willful, anathema”
Sorted list: “willful, vanguard, ungrateful, tepid, special, race, overrated, legal, irrefutable, green, good, foreign, eternally, epiphany, dynamic, dominion, bandit, anathema, albatross, acrobatic”

Original list: “libel, invincible, unremarkable, friendly, albatross, stilted, farcical, unfriendly, dexterity, normal, rapid, our, culture, confederate, almost, purposeful, epistemic, enchantment, disorganized, exorbitant”
Sorted list: “unremarkable, unfriendly, stilted, rapid, purposeful, our, normal, libel, invincible, friendly, farcical, exorbitant, epistemic, enchantment, disorganized, dexterity, culture, confederate, almost, albatross”

Original list: “illogical, hard, scoundrel, over, sequester, irrefutable, lost, anathema, raucous, wondrous”
Sorted list:

G.37 Three-digit multiplication: Number

Evaluate the mathematical expression in Question 2. For example, if you were asked Question 1, you would answer with Answer 1.
Question 1: 831 times 447
Answer 1: 371457

Question 2: 285 times 796
Answer 2:

G.38 Three-digit multiplication: Lowercase

Evaluate the mathematical expression in Question 2. For example, if you were asked Question 1, you would answer with Answer 1.
Question 1: eight hundred thirty-one times four hundred forty-seven
Answer 1: 371457

Question 2: two hundred eighty-five times seven hundred ninety-six
Answer 2:

G.39 Three-digit multiplication: All caps

Evaluate the mathematical expression in Question 2. For example, if you were asked Question 1, you would answer with Answer 1.
Question 1: EIGHT HUNDRED THIRTY-ONE times FOUR HUNDRED FORTY-SEVEN
Answer 1: 371457

Question 2: TWO HUNDRED EIGHTY-FIVE times SEVEN HUNDRED NINETY-SIX
Answer 2:

G.40 Three-digit multiplication: Alternating capitals

Evaluate the mathematical expression in Question 2. For example, if you were asked Question 1, you would answer with Answer 1.
Question 1: eIgHt HuNdReD tHiRtY-oNe times fOuR hUnDrEd FoRtY-sEvEn
Answer 1: 371457

Question 2: tWo HuNdReD eIgHtY-fIvE times sEvEn HuNdReD nInEtY-sIx
Answer 2:

G.41 Linear function

Below is a number. Multiply it by 9/5 and then add 32. Your answer should be a single number:
Input: 328
Answer:

G.42 Linear function with rounding

Below is a number. Multiply it by 9/5 and then add 32. Your answer should be a single number rounded to the nearest integer:
Input: 78
Answer:

G.43 Keyboard cipher: Basic

The keyboard cipher is a cipher where each letter is replaced with the letter to the right of it on a QWERTY keyboard. If the letter does not have a letter to its right, then you should wrap around to the start of the row; e.g., "p" maps to "q". For example, here is a message to be encoded:
Original message: "Hello world!"

To encode this message, we replace each letter with the one to the right of it on a QWERTY keyboard:
1. The first word is "Hello"
  * H -> J
  * e -> r
  * l -> a
  * l -> a
  * o -> p
  So "Hello" becomes "Jraap"
2. The second word is "world!"
  * w -> e
  * o -> p
  * r -> t
  * l -> a
  * d -> f
  * ! -> !
  So "world!" becomes "eptaf!"

Therefore, the message in the keyboard cipher is: "Jraap eptaf!"

Here is another message. Encode the message in the keyboard cipher, word by word and letter by letter. On the last line, write the words "Keyboard cipher:" followed by the encoded message:
Original message: "But this time, there may also be another reason."

G.44 Keyboard cipher: Keyboard provided

The keyboard cipher is a cipher where each letter is replaced with the letter to the right of it on a QWERTY keyboard. If the letter does not have a letter to its right, then you should wrap around to the start of the row; e.g., “p” maps to “q”. For reference, here are the three rows of a QWERTY keyboard:
q w e r t y u i o p
a s d f g h j k l
z x c v b n m

For example, here is a message to be encoded:
Original message: “Hello world!”

To encode this message, we replace each letter with the one to the right of it on a QWERTY keyboard:
1. The first word is “Hello”
  * H -> J
  * e -> r
  * l -> a
  * l -> a
  * o -> p
  So “Hello” becomes “Jraap”
2. The second word is “world!”
  * w -> e
  * o -> p
  * r -> t
  * l -> a
  * d -> f
  * ! -> !
  So “world!” becomes “eptaf!”

Therefore, the message in the keyboard cipher is: “Jraap eptaf!”

Here is another message. Encode the message in the keyboard cipher, word by word and letter by letter. On the last line, write the words “Keyboard cipher:” followed by the encoded message:
Original message: “But this time, there may also be another reason.”

G.45 Keyboard cipher: Correspondences provided

The keyboard cipher is a cipher where each letter is replaced with the letter to the right of it on a QWERTY keyboard. If the letter does not have a letter to its right, then you should wrap around to the start of the row; e.g., “p” maps to “q”. For reference, here are the correct replacements for all letters:
a -> s
b -> n
c -> v
d -> f
e -> r
f -> g
g -> h
h -> j
i -> o
j -> k
k -> l
l -> a
m -> z
n -> m
o -> p

p -> q
q -> w
r -> t
s -> d
t -> y
u -> i
v -> b
w -> e
x -> c
y -> u
z -> x

For example, here is a message to be encoded:
Original message: "Hello world!"

To encode this message, we replace each letter with the one to the right of it on a QWERTY keyboard:
1. The first word is "Hello"
  * H -> J
  * e -> r
  * l -> a
  * l -> a
  * o -> p
  So "Hello" becomes "Jraap"
2. The second word is "world!"
  * w -> e
  * o -> p
  * r -> t
  * l -> a
  * d -> f
  * ! -> !
  So "world!" becomes "eptaf!"

Therefore, the message in the keyboard cipher is: "Jraap eptaf!"

Here is another message. Encode the message in the keyboard cipher, word by word and letter by letter. On the last line, write the words "Keyboard cipher:" followed by the encoded message:
Original message: "But this time, there may also be another reason."

G.46 Birthdays

On what date was Michelle Pfeiffer born? You should format your answer as "Month Day, Year"; e.g., "July 19, 1743."

H Evaluation details

In this section we describe the criteria that we used to determine if a model's output was correct on a given example. One challenge for making this determination was that models often did not adhere to a systematic format for their answers, making it challenging to automatically extract their response from the narration surrounding their response. To address this difficulty, for tasks that have long answers (namely, shift ciphers, reversal, Pig Latin, sorting, and birthdays), we counted the answer as correct as long as the ground truth answer appeared anywhere in the model's response. For the remaining tasks, we extracted the answers from the responses by a combination of automatic methods (i.e., automating the processing of the response for cases that followed a systematic structure) and manual inspection. In the subsections below, we provide more evaluation details that are specific to particular tasks.

H.1 Shift ciphers and reversal

We ignored quotation marks at the start and end of the answer, but otherwise the exact ground truth string had to be contained in the model's output in order to be counted as correct.

H.2 Pig Latin

We ignored quotation marks at the start and end of the answer. When the task was decryption, we accepted any answer that contained a possible decryption of the input, even if it was different from the intended one. The reason to make this allowance is that Pig Latin decryption can be ambiguous; e.g., *identstray* could correspond to *tridents* or *strident*. We also ignored case; since we lowercased the text in the input, we decided not to penalize models for adding capitalization since inserting proper capitalization could reasonably be assumed to be part of the intended decoding process.

H.3 Acronyms

We ignored quotation marks, spaces, and capitalization in the answers. Models often placed their answer within a larger sentence (e.g., *The sequence of letters created when combining the first letters of the words in the given sequence is "CONCORD".*); thus, we manually inspected answers containing spaces to extract just the answer, in order to avoid penalizing models for including this extra material.

H.4 Sorting

We ignored quotation marks in the answers. The model's output had to contain the exact correct output to be counted as correct.

H.5 Counting, linear functions, and three-digit multiplication

A model was considered correct if it got exactly the right number, or incorrect otherwise. We allowed models to use any wording that communicated the correct number (e.g., just saying the number, vs. producing it within a longer sentence; or writing large numbers with or without commas); we allowed for such cases by manually checking any answers that could not be parsed as a number.

H.6 Birthdays

For each model output, we first tried to automatically parse it into a date. If we could not parse it automatically, we checked it manually and converted it into a date if the answer mentioned a date. To be counted as correct, a model had to mention exactly the correct date (day, month, and year).

H.7 Sampling from models

In all cases, we sampled from LLMs using a temperature of 0.0, in order to be generous to the LLMs: Our tasks are deterministic, so there is a single correct answer whose probability should be 1.0. With a temperature of 0.0, an LLM always produces the word that it assigns the highest probability. Thus, as long as the LLM is close to the correct distribution (i.e., correctly identifying which word should have the highest probability), a temperature of 0.0 will ensure that it gets the answer right.

I Statistical tests

I.1 General notes

In our comparisons across conditions (e.g., across output probability levels), we aimed to control our datasets for other variables (e.g., input probability). However, it was not always possible to exactly match these other variables. Therefore, here we run logistic and linear regressions that include predictors for our target variable as well as potential confounding variables, as a way to test whether the target variables have a statistically

significant effect even when other factors are taken into account. All statistical tests were run in R (107) using the `glmnet` package (108, 109). Plots were generated using `ggplot2` (110). In some cases, various predictors of interest are correlated, so we report variance inflation factors (VIFs) as a measure of the extent to which this correlation is problematic. We deem the VIF to be excessive if it is above a threshold of 5. In all cases, we z-scored continuous or integer predictors due to large differences in scale, and we ran separate statistical tests for each LLM. Unless otherwise stated, the response variable was a binary variable that was 1 if the LLM in question produced the correct answer or 0 otherwise.

When we used the number of tokens as a predictor, this number was always computed using the tokenizer for the LLM being analyzed. For example, when running tests focused on Llama 3, we counted tokens using Llama 3's tokenizer. Claude 3's tokenizer is not publicly available, so we did not include token-based predictors in the regressions involving Claude 3.

In some of the logistic regressions, the response variable was the same for all examples in a particular condition (either all examples had a value of 0, or all examples had a value of 1), which creates issues for logistic regression due to complete separation. To address this issue, we augmented the logistic regression with the weak Bayesian prior proposed by (111), implemented in the R package `arm`.[7] The cases where we used this Bayesian augmentation are noted below.

I.2 Shift ciphers

I.2.1 Comparing task variants

To compare rot-13 and rot-12, we used a logistic regression with predictors of shift (a binary variable that could either be a shift of 12 or 13), the number of characters in the sentence (which was the same for the input and the output), the number of subword tokens in the input, the number of subword tokens in the output, the log probability of the input (estimated using GPT-2), and the log probability of the output (estimated using GPT-2):

$$\text{correct} \sim \text{shift} + \text{input\_nchars} + \text{input\_ntokens} + \text{output\_ntokens} \quad (1)$$
$$+ \text{input\_logprob} + \text{output\_logprob} \quad (2)$$

In these tests, we used the weak Bayesian prior (Appendix I.1) to address complete separation issues. For Claude 3, we omitted the input_ntokens and output_ntokens predictors because Claude 3's tokenizer is not publicly available.

There was a statistically significant effect of shift for GPT-4 encoding ($p < 0.01$), Claude 3 encoding ($p < 10^{-9}$), GPT-4 decoding ($p < 0.001$), GPT-3.5 decoding ($p < 0.01$), and Claude 3 decoding ($p < 10^{-10}$). There was not a statistically significant effect of shift for GPT-3.5 encoding ($p = 0.28$) or Llama 3 decoding ($p = 0.09$), likely due to floor effects (GPT-3.5's encoding accuracy was 2% for rot-13 and 0% for rot-12, and LLama 3's decoding accuracy was 5% for rot-13 and 0% for rot-12). In all cases, the shift predictor had a negligible VIF (less than 1.2) meaning that collinearity is not a concern.

We did not run significance tests for Llama 3 encoding, Gemini 1.0 encoding, or Gemini 1.0 decoding, because accuracy was 0% across conditions in these cases.

I.2.2 Input probability

To investigate the effects of input probability, we used a logistic regression with predictors for input and output log probability (estimated using GPT-2), the number of subword tokens in the input and output, and the number of characters in the input (which was the same as the number of characters in the output):

$$\text{correct} \sim \text{input\_logprob} + \text{output\_logprob} + \text{input\_ntokens} + \text{output\_ntokens} + \text{input\_nchars} \quad (3)$$

In these tests, we used the weak Bayesian prior (Appendix I.1) to address complete separation issues. For Claude 3, we omitted the input_ntokens and output_ntokens predictors because Claude 3's tokenizer is not publicly available.

[7] `https://cran.r-project.org/package=arm`

This test was applied to the cases where LLMs were asked to encode sentences of varying levels of probability. The predictor of interest, input log probability, had a statistically significant effect for GPT-4 ($p < 0.05$) and Claude 3 ($p < 0.05$) but not GPT-3.5 ($p = 0.87$). The VIF was small (< 2.0) for input log probability in all three cases. We did not run tests for Llama 3 or Gemini 1.0 because their accuracy was 0.0 across conditions.

I.2.3 Output probability

To investigate the effects of output probability, we used the same variables as for input probability (Appendix I.2.2). Output probability had a statistically significant effect for GPT-3.5 ($p < 10^{-4}$), GPT-4 ($p < 10^{-8}$), Llama 3 ($p < 0.05$), and Claude 3 ($p < 0.001$). We did not run a test for Gemini 1.0 because its accuracy was 0.0 across conditions. The VIF for output log probability was below our threshold of 5.0 in all cases.

I.2.4 Step-by-step and chain-of-thought prompting

We applied the same statistical tests described above to the shift cipher results obtained with GPT-4 using step-by-step and chain-of-thought prompting. We did not use the weak Bayesian prior in these cases because there was no complete separation. Output log probability had a significant effect in all three cases ($p < 10^{-7}$ for basic prompting, $p < 10^{-6}$ for step-by-step prompting, and $p < 10^{-8}$ for chain-of-thought prompting). Input log probability had a significant effect for basic prompting ($p < 0.05$) and chain-of-thought prompting ($p < 0.05$); its effect was not significant for step-by-step prompting but did come close to significance ($p = 0.059$). Finally, when comparing rot-13 to rot-12 (which was done using the Bayesian prior due to complete separation issues), there was a significant effect of the task for both encoding and decoding and for all prompt styles. For encoding, we had $p < 0.01$ for basic prompting, $p < 0.01$ for step-by-step prompting, and $p < 0.05$ for chain-of-thought prompting. For decoding, we had $p < 0.001$ for basic prompting, $p < 0.001$ for step-by-step prompting, and $p < 10^{-4}$ for chain-of-thought prompting. For all the cases discussed in this paragraph, the VIF was small (less than 2.0).

I.2.5 Few-shot learning

We applied the same tests described above to model performance using few-shot learning to encode and decode shift ciphers. First, we tested the effects of input log probability on models encoding text into rot-13. Input log probability did not have a significant effect for GPT-4 with 0-shot in-context learning ($p = 0.29$), 5-shot in-context learning ($p = 0.08$), or 10-shot in-context learning ($p = 0.08$), nor for GPT-3.5 using 0-shot in-context learning ($p = 0.62$), 5-shot in-context learning ($p = 0.18$), or 10-shot in-context learning ($p = 0.34$), nor for Claude 3 using 0-shot in-context learning ($p = 0.75$) or 5-shot in-context learning ($p = 0.08$), nor for GPT-3.5 using 0-shot fine-tuning ($p = 0.62$), 10-shot fine-tuning ($p = 0.74$), or 100-shot fine-tuning ($p = 0.45$). It did have a significant effect for Claude 3 with 10-shot in-context learning ($p < 0.01$). We then tested the effects of output log probability on models decoding rot-13 text. Output log probability had a statistically significant effect ($p < 0.05$) in all 0-shot and few-shot cases except for Claude 3 using 10-shot in-context learning ($p = 0.07$). The relevant VIFs were below our threshold of 5 in all cases.

Finally, we studied the effect of task variant by comparing performance on rot-12 with performance on rot-13. For the GPT models, 0-shot and few-shot models do not differ from each in terms of whether they show a statistically significant effect of task. GPT-4 performing encoding shows a statistically significant effect of task across 0-shot, 5-shot and 10-shot in-context learning ($p < 0.05$). GPT-3.5 performing encoding does not show a significant effect of task whether doing 0-shot ($p = 0.07$), 5-shot ($p = 0.09$), or 10-shot ($p = 0.11$) in-context learning, or 0-shot ($p = 0.07$), 10-shot ($p = 0.15$), or 100-shot ($p = 0.17$) fine-tuning. When decoding, all GPT conditions (GPT-4 using in-context learning, GPT-3.5 using in-context learning, and GPT-3.5 using fine-tuning) showed a statistically significant effect of task across all numbers of demonstrations tested ($p < 0.01$). Unlike the GPT models, Claude 3 showed differences between 0-shot and few-shot models. The 0-shot conditions with Claude 3 showed significant effects of task for both encoding ($p < 10^{-6}$) and decoding ($p < 10^{-11}$), but the 5-shot and 10-shot conditions did not show significant effects for either encoding ($p = 0.96$ for the 5-shot condition, and $p = 0.57$ for the 10-shot condition) or decoding ($p = 0.53$ for the 5-shot condition, and $p = 0.84$ for the 10-shot condition).

In follow-up experiments based on words where there was no overlap in the letters used between the demonstration words and the test word, we found that task variant continued to have a statistically significant effect in all GPT-3.5 cases for both encoding and decoding ($p < 0.01$), all GPT-4 cases for both encoding and decoding ($p < 0.001$), and all Claude 3 cases for both encoding and decoding ($p < 0.001$). In the condition where there was overlap between the demonstration words and prompt words, shift level continued to have a statistically significant effect for all GPT-3.5 cases for both encoding and decoding ($p < 0.01$) and all GPT-4 cases for both encoding and decoding ($p < 0.001$). For Claude 3, task variant also continued to have a statistically significant effect for all decoding cases ($p < 0.001$), but for encoding it was significant only for the 0-shot case ($p < 10^{-9}$) but not the few-shot cases ($p = 0.13$ for 5-shot learning, and $p = 0.08$ for 10-shot learning). The relevant VIFs were below our threshold of 5 in all cases.

I.3 Reversal

I.3.1 Input probability

We used logistic regressions with the same variables as used for shift ciphers (Appendix I.2.2). Input probability did not have a statistically significant effect for GPT-3.5 ($p = 0.22$), GPT-4 ($p = 0.85$), or Llama 3 ($p = 0.33$), but it did have a statistically significant effect for Claude 3 ($p < 10^{-15}$) and Gemini 1.0 ($p < 10^{-4}$). The VIF for input log probability was below our threshold of 5.0 in all cases, meaning that collinearity is not a serious concern for these results.

I.3.2 Output probability

We used a logistic regression with the same variables as used for shift ciphers (Appendix I.2.2). Output probability had a statistically significant effect for all models, with $p < 10^{-7}$ for GPT-3.5, $p < 10^{-11}$ for GPT-4, $p < 10^{-5}$ for Llama 3, $p < 10^{-15}$ for Claude 3, and $p < 10^{-12}$ for Gemini 1.0. The VIF for output log probability was below our threshold of 5.0 in all cases, meaning that all were within the range we considered acceptable. We therefore conclude that collinearity is unlikely to be a serious problem for drawing conclusions from these tests.

I.3.3 Few-shot learning

We used the same tests described above to analyze models that performed reversal with few-shot learning. Specifically, we investigated both input probability and output probability for all few-shot learning setups that we considered: GPT-3.5, GPT-4, and Claude 3 with 0-shot, 5-shot, and 10-shot in-context learning, and GPT-3.5 with 0-shot, 10-shot, and 100-shot fine-tuning.

Input log probability had a statistically significant effect in GPT-4 with 5-shot in-context learning ($p < 0.001$), GPT-4 with 10-shot in-context learning ($p < 10^{-4}$), GPT-3.5 with 5-shot in-context learning ($p < 0.05$), and Claude 3 in 0-shot ($p < 10^{-7}$), 5-shot ($p < 10^{-15}$), and 10-shot ($p < 10^{-15}$) in-context learning. Input log probability did not have a statistically significant effect for GPT-4 with 0-shot in-context learning ($p = 0.84$), GPT-3.5 with 0-shot ($p = 0.35$) or 10-shot ($p = 0.18$) in-context learning, or any cases of GPT-3.5 with fine-tuning ($p = 0.35$ for 0-shot fine-tuning, $p = 0.43$ for 10-shot fine-tuning, and $p = 0.20$ for 100-shot fine-tuning).

Output log probability had a statistically significant effect in all few-shot cases (with $p < 0.001$) except for GPT-3.5 with 10-shot fine-tuning ($p = 0.40$) and GPT-3.5 with 100-shot fine-tuning ($p = 0.80$).

The relevant VIFs were below our threshold of 5.0 in all cases.

I.4 Pig Latin

I.4.1 Comparing task variants

To compare Pig Latin and Boar Etruscan, we used a logistic regression with predictors of task (a binary variable that could either be Pig Latin or Boar Etruscan), the number of characters in the input, the number of characters in the output, the number of subword tokens in the input, the number of subword tokens in the output, the log probability of the input (estimated using GPT-2), and the log probability of the output (estimated using GPT-2):

$$\begin{aligned} \text{correct} \sim\ & \text{task} + \text{input\_nchars} + \text{output\_nchars} + \text{input\_ntokens} + \text{output\_ntokens} && (4)\\ & + \text{input\_logprob} + \text{output\_logprob} && (5) \end{aligned}$$

In these tests, we used the weak Bayesian prior (Appendix I.1) to address complete separation issues. For Claude 3, we omitted the input_ntokens and output_ntokens predictors because Claude 3's tokenizer is not publicly available.

There was a statistically significant effect of task for GPT-3.5 encoding ($p < 0.01$), GPT-4 encoding ($p < 0.001$), Claude 3 encoding ($p < 10^{-11}$), GPT-4 decoding ($p < 0.05$), and Llama 3 decoding ($p < 0.001$), but not Llama 3 encoding ($p = 0.12$), GPT-3.5 decoding ($p = 0.15$), Claude 3 decoding ($p = 0.65$), or Gemini 1.0 decoding ($p = 0.33$). We do not include a test for Gemini 1.0 encoding because its accuracy was 0% for both variants. In all cases where we ran tests, the task predictor had a small VIF (less than 2.0) meaning that collinearity is not a concern.

To compare different variants of Pig Latin, we used a similar logistic regression as the one used for Pig Latin vs. Boar Etruscan, except instead of a binary categorical variable for task we used a continuous variable of task probability (which we defined as equal to the proportion of Pig Latin instances in C4 that used the Pig Latin variant in question). We continued to use the Bayesian prior due to complete separation. For encoding text into Pig Latin, we found a statistically significant effect of task for GPT-3.5 ($p < 10^{-6}$), GPT-4 ($p < 10^{-8}$), Claude 3 ($p < 0.01$), and Gemini 1.0 ($p < 0.01$), but not for Llama 3 ($p = 0.19$). For decoding text from Pig Latin, there was no significant effect of task for GPT-3.5 ($p = 0.31$), GPT-4 ($p = 0.06$), or Llama 3 ($p = 0.06$), but there was a significant effect for Claude 3 ($p < 0.01$) and Gemini 1.0 ($p < 0.01$). Across all tests, the VIF for the task probability variable was small (less than 1.1), meaning that collinearity was not a concern for these regressions.

I.4.2 Input probability

To test the effects of input probability, we analyzed the results for models translating from English to Pig Latin. We used a logistic regression with the same variables as used for shift ciphers (Appendix I.2.2) except with a predictor added for the number of characters in the output (output_nchars) because for Pig Latin (unlike for shift ciphers) this quantity differs from the number of characters in the input. Input probability did not have a statistically significant effect for GPT-3.5 ($p = 0.70$), GPT-4 ($p = 0.88$), or Llama 3 ($p = 0.21$), but it did have a significant effect for Claude 3 ($p < 0.01$). We do not include a test for Gemini 1.0 because its encoding accuracy was 0% across conditions. The VIF for input log probability was modest for all models (less than 2.1), meaning that collinearity is not a serious concern for these results.

I.4.3 Output probability

We used a logistic regression with the same variables as used for investigating input probability (Appendix I.4.2). Output probability had a statistically significant effect for all models ($p < 10^{-11}$ for GPT-3.5, $p < 10^{-14}$ for GPT-4, $p < 10^{-5}$ for Llama 3, $p < 10^{-13}$ for Claude 3, and $p < 10^{-6}$ for Gemini 1.0). The VIF for output log probability was below our threshold of 5.0 for all models, meaning that collinearity is not a major concern for these results.

I.5 Article swapping

I.5.1 Main experiment

We first analyzed the effect of example probability using a logistic regression with predictors for input and output log probability (estimated using GPT-2), the number of subword tokens in the input (which was the same as the number in the output), and the number of characters in the input (which was the same as the number in the output). For Claude 3, we omitted the input_ntokens predictor because Claude 3's tokenizer is not publicly available:

$$\text{correct} \sim \text{input\_nchars} + \text{input\_ntokens} + \text{input\_logprob} + \text{output\_logprob} \quad (6)$$

However, with this model, input log probability and output log probability had excessives VIFs of over 5 for all five LLMs. We therefore reran the test without input log probability, leaving just output log probability to stand for the overall example probability. In this case, probability had a significant effect for all models, with $p < 10^{-11}$ for GPT-3.5, $p < 10^{-15}$ for GPT-4, $p < 10^{-4}$ for Llama 3, $p < 10^{-10}$ for Claude 3, and $p < 10^{-10}$ for Gemini 1.0. The VIFs for probability were reasonable in all cases, with values less than 2.5.

I.5.2 Expanded experiment

The tests run in the previous subsection were unable to determine whether the effect of probability was due to the probability of the input or the probability of the output. We ran another series of experiments to attempt to disentangle these effects. In the article swapping task that we have focused on, the input is an ungrammatical sentence that becomes grammatical when each article is swapped with the word before it. We now consider three additional framings of this task (based on which direction the swap occurs in and whether it is the input or output that is ungrammatical), to give four total task versions, illustrated in Table S7. These variants yield widely varying accuracies.

We first consider GPT-3.5 and GPT-4, whose results showed similar patterns to each other. For these models, the direction of the swap (next vs. previous) does not appear to be the main driver of the differences in accuracy because, for both of these models, the "previous" direction yields both the highest-accuracy setting and the lowest-accuracy setting. The input also does not appear to be the main driver of accuracy differences for these two models: if it were, we would expect the highest accuracies in the conditions with a grammatical input, but in fact those conditions get the lowest accuracies. What does appear to matter most is the output: the conditions with the grammatical outputs yield the highest accuracies for these models. To be sure, this is not the only factor influencing the results: it appears the the "ungramm.-P" sentences are harder to process than the "ungramm.-N" ones, since for both encoding and decoding the condition with "ungramm.-P" has lower accuracies than the one with "ungramm.-N." However, given that the most extreme differences in accuracy seem to be driven by the type of output that is produced, we conclude that output probability (rather than input probability) is mainly responsible for the highly significant effect of probability that we observed in Appendix I.5.1.

Now we consider the other three models: Llama 3, Claude 3, and Gemini 1.0. If input probability were the main driver of performance, we would expect the two conditions with "gramm." input to have similar accuracies because they share inputs, but in fact these two conditions differ markedly in accuracy across models (e.g., in Claude 3, one has an accuracy of 0.62 while the other has an accuracy of 0.03). In contrast, the two conditions that have a grammatical output have roughly similar accuracies for all three of these models. In sum, sharing an output seems to lead to similar accuracies across conditions, while sharing an input does not. We take these observations as evidence that the output is a more important driver of model performance than the input, which supports the conclusion that output probability, not input probability, is mainly responsible for the significant effects of probability that we observed in Appendix I.5.1 for these three models (similar to the conclusion that we reached for GPT-3.5 and GPT-4 in the previous paragraph).

I.6 Acronyms

I.6.1 Comparing task variants

We evaluated the effect of task using the logistic regression below. The task variable was a binary categorical variable that could either be the first-letter acronym task or the second-letter acronym task. For GPT-3.5, GPT-4, and Llama 3, we did not include predictors for example length because all examples had the same length, whether counted in characters or in subword tokens. For Gemini 1.0, we added predictors for input_ntokens and output_ntokens because the number of tokens did vary for this model (the number of tokens was controlled for using the GPT tokenizer, but the Gemini 1.0 tokenizer sometimes varied from this tokenizer; the Llama 3 tokenizer did not vary from the GPT tokenizer for this dataset). We did not include token-based predictors for Claude 3 because its tokenizer is not publicly available.

$$\text{correct} \sim \text{task} + \text{input\_logprob} + \text{output\_logprob} \tag{7}$$

The log probability of an individual word was as described in Appendix F.6 (based on GPT-2's probability of the word in the context *The word is "WORD"*). Since the inputs were multiple words, their log probability

Task version	Example	Input	Output	Direction	Accuracy: GPT-3.5	Accuracy: GPT-4	Accuracy: Llama 3	Accuracy: Claude 3	Accuracy: Gemini 1.0
Encoding: Swap with next	I will not give up even for a moment. → I will not give up even for moment a.	Gramm.	Ungramm.-N	Next	0.23	0.57	0.57	0.62	0.56
Encoding: Swap with previous	I will not give up even for a moment. → I will not give up even a for moment.	Gramm.	Ungramm.-P	Previous	0.01	0.05	0.00	0.03	0.01
Decoding: Swap with next	I will not give up even a for moment. → I will not give up even for a moment.	Ungramm.-P	Gramm.	Next	0.32	0.62	0.22	0.42	0.60
Decoding: Swap with previous	I will not give up even for moment a. → I will not give up even for a moment.	Ungramm.-N	Gramm.	Previous	0.65	0.83	0.26	0.53	0.53

Table S7: Four variants of the article swapping task. *Encoding* refers to a variant that converts a grammatical sentence to an ungrammatical one; *decoding* is the reverse. *Gramm.* and *ungramm.* stand for *grammatical* and *ungrammatical*. In more detail, *ungramm.-N* refers to an ungrammatical sentence created by starting with a grammatical sentence and swapping each article with the next word; *ungramm.-P* is analogous but uses swapping with the previous word. *Direction* refers to whether the task involves swapping articles with the next or previous word. The accuracies shown here are all based on the high-probability set of grammatical sentences.

was taken to be the sum of the log probabilities of the words in them. In these tests, we used the weak Bayesian prior (Appendix I.1) to address complete separation issues.

The factor of interest was task, which had a significant effect for all models, with $p < 10^{-4}$ for GPT-3.5, $p < 10^{-15}$ for GPT-4, $p < 10^{-15}$ for Llama 3, $p < 10^{-5}$ for Claude 3, and $p < 10^{-5}$ for Gemini 1.0. In all cases, the VIF for this factor was negligible (less than 1.02), showing that collinearity was not a concern.

I.6.2 Input probability

We evaluated the effect of input probability using the logistic regression below. For the same reasons as discussed in the task variant comparison (Section I.6.1), we also added predictors for input_ntokens and output_ntokens for Gemini 1.0 but not for any other models. We did not use the Bayesian prior in this case because there was no complete separation:

$$\text{correct} \sim \text{input\_logprob} + \text{output\_logprob} \tag{8}$$

The factor of interest was input log probability, which had a significant effect for GPT-3.5 ($p < 10^{-5}$) and Llama 3 ($p < 10^{-4}$), but not GPT-4 ($p = 0.09$), Claude 3 ($p = 0.06$), or Gemini 1.0 ($p = 0.43$). In all cases, the VIF was below our threshold of 5.0, so collinearity was not a concern.

I.6.3 Output probability

To investigate the effect of output probability, we used the same logistic regression setup as for input probability (Appendix I.6.2). Output probability had a significant effect for GPT-3.5 ($p < 10^{-15}$), GPT-4 ($p < 10^{-9}$), Llama 3 ($p < 10^{-9}$), and Gemini 1.0 ($p < 0.01$), but not for Claude 3 ($p = 0.38$). The VIF was negligible (less than 1.1) in all cases, so collinearity was not a concern.

I.7 Counting

To investigate the effect of example probability on counting success, we used logistic regressions with predictors for the magnitude of the number being counted to, the log probability of the input sequence, and the

log probability of the output (i.e., the count):

$$\text{correct} \sim \text{magnitude} + \text{input\_logprob} + \text{output\_logprob} \tag{9}$$

For counting characters, we also included a predictor for the number of tokens in the input, to control for the fact that the characters in the low-probability case (i.e., emojis) typically require more tokens than the characters in the high-probability case (i.e., letters). This predictor was not used in the case of counting words because the number of tokens was matched across the conditions with common words and rare words. It was also not used for Claude 3, whose tokenizer is not publicly available.

For counting words, the input log probability was the mean of word log probabilities, where a word's log probability is the log probability assigned by GPT-2 to the sequence *The word is "WORD"* minus the log probability for *The word is "*. For characters, we counted how often each character appeared in the first 1 billion words of the C4 corpus (11), and added 1 to each count (add-1 smoothing) to deal with the nonappearance of some emojis. The output log probability was estimated by counting how many times each number appeared in the first 1 million integers between 1 and 100 mentioned in the C4 corpus, then normalizing.

When the inputs were all common and the outputs could be any number from 1 to 100, output log probability had a significant effect for GPT-3.5, GPT-4, Claude 3, and Gemini 1.0, whether they were processing characters or words ($p < 10^{-15}$ in all cases). It also had a significant effect for Llama 3 counting characters ($p < 10^{-15}$) but not for Llama 3 counting words ($p = 0.12$). Magnitude had a significant effect for Llama 3 counting words ($p < 10^{-15}$), Claude 3 counting words ($p < 10^{-15}$; note that the effect in this case is in the opposite direction of the others, with higher magnitudes being associated with higher accuracy), Gemini 1.0 counting words ($p < 10^{-6}$), GPT-3.5 counting characters ($p < 10^{-15}$), GPT-4 counting characters ($p < 10^{-14}$), Llama 3 counting characters ($p < 10^{-15}$), Claude 3 counting characters ($p < 0.01$), and Gemini 1.0 counting characters ($p < 10^{-4}$), but not for GPT-3.5 counting words ($p = 0.56$) or GPT-4 counting words ($p = 0.07$). For all cases, the VIFs for both magnitude and output log probability are below our threshold of 5.0, so we conclude that collinearity is not a concern.

We ran the same tests on the case where we performed a controlled comparison between high-probability counts and low-probability counts. Output log probability was also significant here for GPT-3.5, GPT-4, Claude 3, and Gemini 1.0 ($p < 10^{-15}$ for all four models, regardless of whether words or characters were counted), and it was also significant for Llama 3 counting characters ($p < 10^{-15}$) but not Llama 3 counting words ($p = 0.62$). The VIFs continued to be reasonable.

When we compared the counting of low-probability inputs with high-probability ones, we found that input log probability had a statistically significant effect for GPT-3.5 counting words ($p < 0.05$), GPT-4 counting words ($p < 0.05$), Gemini 1.0 counting words ($p < 0.05$), GPT-3.5 counting characters ($p < 10^{-10}$), GPT-4 counting characters ($p < 10^{-6}$), Llama 3 counting characters ($p < 0.01$), Claude 3 counting characters ($p < 10^{-15}$), and Gemini 1.0 counting characters ($p < 10^{-7}$), but not for Llama 3 counting words ($p = 0.71$) or Claude 3 counting words ($p = 0.10$). Note that, for GPT-3.5 counting words, GPT-4 counting characters, Llama 3 counting characters, and Claude 3 counting characters, the effect of input probability was in the opposite direction from what we expected: higher-probability inputs were associated with worse scores, rather than better ones. The VIF for input log probability was below our threshold of 5 for all conditions.

I.7.1 Few-shot learning

For the few-shot counting experiments, we used the same test as we did for the basic counting experiments described in Appendix I.7, except that we also added a predictor for demonstration_count, which was equal to the number of times that the correct answer had appeared as the answer for one of the demonstrations that were provided either in-context or during fine-tuning. This predictor was included for 5-shot, 10-shot, and 100-shot experiments, but not for 0-shot experiments because there were no demonstrations in those cases. The overall logistic regression with the demonstration count included can be expressed as follows:

$$\text{correct} \sim \text{magnitude} + \text{input\_logprob} + \text{demonstration\_count} + \text{output\_logprob} \tag{10}$$

We ran this test for all few-shot conditions: GPT-3.5 fine-tuned with 0-shot, 10-shot, or 100-shot datasets; and GPT-3.5, GPT-4, and Claude 3 performing in-context learning with 0-shot, 5-shot, and 10-shot datasets.

For all of these conditions, output log probability had a statistically significant effect ($p < 10^{-12}$ in all cases). Demonstration count had a statistically significant effect for GPT-4 doing 10-shot in-context learning ($p < 0.001$, but in the opposite direction from what is expected: lower demonstration frequency is associated with higher accuracy) and for GPT-3.5 doing 100-shot fine-tuning ($p < 0.001$), but it was not significant for the other cases. In all cases, the VIFs for output_logprob and demonstration_count were below our threshold of 5.

I.8 Sorting

To compare different sorting directions (alphabetical vs. reverse alphabetical, or ascending vs. descending) we used logistic regressions with predictors for the direction, the number of characters (which was the same for the input and output), the number of subword tokens (which was the same for the input and output), the input log probability, and the output log probability. For Claude 3, we omitted the ntokens predictor because Claude 3's tokenizer is not publicly available. For Gemini 1.0 sorting numbers, we omitted the ntokens predictor because, for this task, it was perfectly correlated with the nchars predictor:

$$\text{correct} \sim \text{direction} + \text{nchars} + \text{ntokens} + \text{input\_logprob} + \text{output\_logprob} \tag{11}$$

These regressions found that, for sorting words, direction had a significant effect for all models ($p < 10^{-8}$ for GPT-3.5, $p < 10^{-6}$ for GPT-4, $p < 10^{-6}$ for Llama 3, $p < 10^{-5}$ for Claude 3, and $p < 10^{-10}$ for Gemini 1.0). For sorting numbers, direction did not have a significant effect for any models ($p = 0.60$ for GPT-3.5, $p = 0.96$ for GPT-4, $p = 0.86$ for Llama 3, $p = 1.0$ for Claude 3, and $p = 0.18$ for Gemini 1.0). The VIF for the task predictor was below our threshold of 5.0 for all cases, so we conclude that collinearity is not a major concern.

We also used the same setup to test for the effect of direction in few-shot settings with alphabetical vs. reverse alphabetical order. Direction had a statistically significant effect in all few-shot settings ($p < 0.01$), and the VIFs were below our threshold of 5.0 in all few-shot cases.

I.9 Linear functions

I.9.1 Comparing task variants

To compare the two linear functions, we used a logistic regression with predictors for the task (a binary variable that could be the task $(9/5)x + 32$ or the task $(7/5)x + 31$), the continuous input value, and the continuous output value:

$$\text{correct} \sim \text{task} + \text{input} + \text{output} \tag{12}$$

In these tests, we used the weak Bayesian prior (Appendix I.1) to address complete separation issues. The factor of interest was task, which had a significant effect for GPT-3.5 ($p < 0.001$), GPT-4 ($p < 0.01$), and Claude 3 ($p < 10^{-8}$)) but not Llama 3 ($p = 0.22$) or Gemini 1.0 ($p = 0.63$). The VIF was below our threshold of 5.0 in all cases.

We repeated these tests for the setting in which the output (rather than the input) was controlled across tasks. The variable of task continued to have a significant effect for GPT-3.5 ($p < 10^{-7}$), GPT-4 ($p < 0.001$), and Claude 3 ($p < 10^{-8}$) but not for Llama 3 ($p = 0.19$) or Gemini 1.0 ($p = 0.18$), and the VIF was still below our threshold of 5.0 in all cases.

I.9.2 Comparing prompts

To compare the different prompts, we used a mixed-effects logistic regression with a fixed effect for prompt type (a 3-way contrast, sum coded) and a random intercept for item:

$$\text{correct} \sim \text{prompt} + (1|\text{item}) \tag{13}$$

We used the weak Bayesian prior (Appendix I.1) to address complete separation issues. We did not include effects for input or output since the by-item random intercept subsumes these factors. For Llama 3 and

	GPT-3.5	GPT-4	Llama 3	Claude 3	Gemini 1.0
Number vs. lowercase	***	n.s.	n.s.	n.s.	n.s.
Number vs. all caps	***	n.s.	n.s.	n.s.	n.s.
Number vs. alternating capitals	***	***	**	**	**
Lowercase vs. all caps	n.s.	n.s.	n.s.	n.s.	n.s.
Lowercase vs. alternating capitals	*	***	*	***	n.s.
All caps vs. alternating capitals	*	***	n.s.	***	n.s.

Table S8: Statistical significance of the differences between LLM accuracy for three-digit multiplication when using different prompt formats. Key: n.s. = not significant; * = $p < 0.05$; ** = $p < 0.01$; *** = $p < 0.001$.

Claude 3, the model failed to converge, so we backed off by removing the by-item random intercept and adding in effects for input and output (since there was no longer a by-item random intercept subsuming these factors).

For each LLM, we first tested whether there was a significant effect of prompt type by using a chi-square test to compare the full model described above with a null model that removed the fixed effect of prompt. These tests found a significant effect of prompt for all LLMs tested ($p < 10^{-18}$ for GPT-3.5; $p < 10^{-21}$ for GPT-4; $p < 0.01$ for Llama 3; $p < 10^{-28}$ for Claude 3; and $p < 10^{-61}$ for Gemini 1.0). Since the chi-square test found a significant effect of prompt, we then tested which pairwise comparisons of prompt types were significant by applying the R package `multcomp` (112), which controls for multiple comparisons, to the full model that had a fixed effect for prompt method. We found that, for GPT-3.5, GPT-4, and Gemini 1.0, all three comparisons were significant, with $p < 0.05$ in all cases; for Llama 3, no comparisons were significant; for Claude 3, there was a significant difference between the primed and basic conditions ($p < 0.001$), and there was a significant difference between the priming control and primed conditions ($p < 0.001$), but there was no significant difference between the priming control and basic conditions.

I.10 Three-digit multiplication

To compare the four different ways of formatting the numbers in the prompt, we used a mixed-effects logistic regression with a fixed effect for the prompt format (a 4-way contrast, sum-coded) and a random intercept for item:

$$\text{correct} \sim \text{format} + (1|\text{item}) \tag{14}$$

The procedure for assessing the significance of differences between prompt formats was the same as the procedure for comparing linear function prompts (Appendix I.9.2). For each LLM, we first tested if the format had a significant effect by running a chi-square test that compared the full model to a null model that removed the fixed effect of format. For all LLMs tested, the chi-square test found a significant effect for format ($p < 10^{-15}$ for GPT-3.5; $p < 10^{-8}$ for GPT-4; $p < 0.01$ for Llama 3; $p < 10^{-8}$ for Claude 3; and $p < 0.001$ for Gemini 1.0). Since the chi-square test found that the format had a significant effect, we tested which pairs of formats were significantly different from each other using the `multcomp` package. Many of the differences had a significant effect; see Table S8. Perhaps most notably, the difference between the number condition and the alternating capitals condition was significant for all five LLMs.

I.11 Birthdays

To investigate the effect of the frequency of a person being mentioned on model accuracy at naming the person's birthday, we ran logistic regressions with predictors for input log probability and output log probability:

$$\text{correct} \sim \text{input\_logprob} + \text{output\_logprob} \tag{15}$$

The input probability (i.e., the probability of the name that is being asked about) was proportional to the number of occurrences of the name in the C4 corpus. The output probability (i.e., the probability of the

date that was the answer) was proportional to the number of entries in the WikiBio dataset that were born on each date (106). For all five LLMs, input log probability had a highly statistically significant effect, with $p < 10^{-15}$. In all cases, the VIF of input log probability was negligible (less than 1.01).

J Estimating example probability

To estimate the probability of the input or output string used in an example, we used GPT-2 (96). GPT-2, like all autoregressive language models, assigns a probability to each token in a sequence. The probability of the whole sequence can then be obtained by multiplying the probabilities of the tokens that make up the sequence. Though GPT-2 is no longer the state-of-the-art language model, its scores at modeling word probabilities in text corpora are nonetheless strong (96), and the probabilities that it produces correlate well with psycholinguistic measures in humans (113), suggesting that GPT-2 is a reasonable choice for estimating probabilities of pieces of text. To estimate the probability of single words, we used the probability that GPT-2 assigned to *The word is "WORD"* minus the log probability that it assigned to *The word is "*; this difference would result in the probability of the word followed by the closing quotation mark conditioned on the prefix *The word is "*. We used this framing as a context in which any word could reasonably appear, and we included the closing quotation mark as a clear indicator of when the word ended. To estimate the probability of examples that were sentences, we used the probability assigned by GPT-2 to that sentence without any additional context.

K Estimating task probability

To estimate the probability of task variants, we performed corpus analyses of the C4 corpus (11), specifically the version hosted by Hugging Face Datasets (114).

It is challenging to precisely count how many occurrences of a task there are in a naturalistic corpus because task instances are typically not labeled as such. As a proxy for counting task instances, we counted phrases that are often used to invoke each task. Below we discuss precisely how we carried this out for each type of task, including discussion of the assumptions that underlie each investigation. The reliance on these (certainly imperfect) assumptions is a limitation of these analyses; the other main limitation is that, in many cases, we had to use manual analysis of data to ensure accuracy, but due to the large amount of data we did not scrutinize all examples carefully, meaning that some examples may have been missed or misclassified. Though these analyses are certainly imperfect, they are useful for giving a rough sense of how frequent each task variant is.

K.1 Shift ciphers

We extracted all examples within the corpus that contained any of the following types of strings. The search was done in a case-insensitive way. These string templates are intended to capture the most frequent ways of referring to shift ciphers:

- **shift cipher**
- **Caesar cipher** (another common term for a shift cipher)
- **rotN** (for all values of N from 1 to 25)
- **rot-N** (for all values of N from 1 to 25)
- **the next letter in the alphabet** (often used when discussing rot-1)
- **the previous letter in the alphabet** (often used when discussing rot-25)

We then manually checked all examples returned by this search. Many of the examples were not in fact discussing a shift cipher. For instance, there is a gene called ROT2 which should not be counted as a shift cipher; there is a scheme for encoding numbers called rot-5, which we also did not count because it is different

from the text encoding scheme rot-5; and the phrase *the next/previous letter in the alphabet* can be used in non-cipher-related contexts. We excluded such examples to give us only the examples that discuss shift ciphers. We then categorized which shift level was being discussed in each case (some examples were excluded because they did not mention a specific shift level). If a single document included several instances of the above phrases, we only counted that document once. In order to count an example as an instance of a shift, we did not require there to be examples of encoded text because checking for such examples would have further lengthened an already labor-intensive search. The resulting counts are shown in the top of Figure 7 in the main paper.

Instead of searching for phrases used to talk about shift ciphers, we considered searching for text encoded in shift ciphers by searching for the encoded forms of various common words. We attempted this approach but found that it ran into unexpected challenges. Words sometimes become other words when shifted, yielding far more false positives than we could manually check; yet we also could not simply exclude shifted candidates that become other words because often the false positives were strings that would not typically be recognized as a word; e.g., *stop* gave us the false positive *rsno*, which would be hard to exclude because it does not typically show up on word lists, yet which does occur in C4 as an abbreviation for the Royal Scottish National Orchestra.

The assumptions underlying this investigation include:

- The distribution of shifts in C4 is similar to the distribution of shifts in the training data for the LLMs that we tested.
- The texts returned by the key phrases described above are representative of the full set of texts mentioning shift ciphers.
- A shift's probability of being illustrated is proportional to its probability of being mentioned (this assumption relates to the fact that we did not require an example to be included, whereas models are most likely to only learn about a shift from cases with examples).

K.2 Pig Latin

We extracted all examples from the corpus that, when lowercased, contained the string *pig latin*. We then manually examined each occurrence to categorize which variant of Pig Latin was used. We only counted examples which specified a variant, either by verbally describing the rule or by providing examples that made the rule clear. As with shift ciphers, we did not require the text to include a translated example to be counted. This search resulted in a count of 32 instances of the variant using *-way*, 22 instances of the variant using *-ay*, 12 instances of the variant using *-yay*, and 9 instances of the variant using *-hay*.

This sample size is relatively small. However, we conducted a similar analysis on the Pile dataset (50) and got similar results: 68 instances of *-way*, 46 instances of *-ay*, 26 instances of *-yay*, and 8 instances of *-hay*. Thus, the rough frequencies of each variant seem reasonably reliable. Our results are also broadly similar to the counts found in the survey of (115), with the major exception that they had many more respondents use *-ay* than *-way*; their population of respondents may have different Pig Latin preferences due to the difference in medium (online text vs. survey response) or due to time (their survey was collected in 2003, whereas most text in C4 and the Pile is more recent).

The following assumptions underlie this investigation:

- The distribution of variants in C4 and the Pile is similar to the distribution of variants in the training data for the LLMs that we tested.
- The texts returned by searching for the phrase *pig latin* are representative of the full set of texts discussing Pig Latin.
- A variant's probability of being illustrated is proportional to its probability of being mentioned.

K.3 Sorting

To estimate the relative frequency of alphabetical order and reverse alphabetical order in the C4 corpus, we counted the occurrences of the phrases *in alphabetical order* and *in reverse alphabetical order*. They occurred

95,492 times and 629 times, respectively, from which we estimate that alphabetical order is approximately 150 times as common as reverse alphabetical order. Similarly, to compare ascending order and descending order, we counted the occurrences of the phrases *in ascending order*—which occurred 21,562 times—and *in descending order*—which occurred 31,378 times. We thus estimate that descending order is approximately 1.5 times as common as ascending order.

These analyses assume that the frequency of these phrases is proportional to the frequency of each type of ordering. This assumption is likely imperfect; in particular, it might be that alphabetical order and ascending order are the default ways of ordering words and numbers, such that they are sometimes used without any explicit mention of the type of ordering that occurs. Nonetheless, these analyses provide one piece of evidence that alphabetical order is much more common than reverse alphabetical order in Internet text, but that ascending and descending order do not have as large of a difference in usage frequency.

L Spelling

To evaluate whether models have access to the spelling of their tokens, we asked them to spell 1,000 words. We first produced a list of all words that appeared among the first 1 billion words in the Pile dataset that fulfilled two conditions. First, the word had to be a single token in the tokenization scheme used by GPT-3.5 and GPT-4, both when the word appeared after a space and when it did not.[8] Second, the word had to consist entirely of lowercase letters from the Roman alphabet. We then randomly selected 1,000 words from this list of candidates. Models were asked to spell the word with a prompt of the following form:

(15) Spell the word "additional" by listing its letters, in order, separated by spaces.

The inclusion of spaces in the output forces the model to break the token down into individual letters.

GPT-4 scored 99.9% on this test. Its only error was producing *b e c k e n d* when the correct answer was *b a c k e n d*. When evaluated strictly, GPT-3.5 scored 98.1%; of its 19 errors, 17 took the form of using hyphens rather than spaces (e.g., responding *i-n-d-u-s-t-r-y* when the target response was *i n d u s t r y*). We view these errors as orthogonal to our question of whether models encode the spelling of words—they reveal failure at following instructions rather than failure at spelling—so we counted GPT-3.5 as correct in these cases, yielding an accuracy of 99.8%. The remaining models also attained over 99% accuracy: 99.8% for Claude 3, 99.5% for Llama 3, and 99.6% for Gemini 1.0. We thus conclude that all of the models that we evaluated robustly encode the spellings of their subword tokens.

SI References

[1] S. Bubeck, et al., Sparks of artificial general intelligence: Early experiments with GPT-4. arXiv [Preprint] (2023) `https://arxiv.org/abs/2303.12712` (accessed 28 August 2024).

[2] T. Brown, et al., Language models are few-shot learners in Advances in Neural Information Processing Systems, eds. H. Larochelle, M. Ranzato, R. Hadsell, M. F. Balcan, H. Lin. Vol. 33, pp. 1877–1901 (2020).

[3] Z. Zhao, E. Wallace, S. Feng, D. Klein, S. Singh, Calibrate before use: Improving few-shot performance of language models in International Conference on Machine Learning. (PMLR), pp. 12697–12706 (2021).

[4] N. Kassner, H. Schütze, Negated and misprimed probes for pretrained language models: Birds can talk, but cannot fly in Proceedings of the 58th Annual Meeting of the Association for Computational Linguistics. (Association for Computational Linguistics, Online), pp. 7811–7818 (2020).

[5] F. Shi, et al., Large language models can be easily distracted by irrelevant context in International Conference on Machine Learning. (PMLR), pp. 31210–31227 (2023).

[8] For the non-GPT models, there is no guarantee that these words will be a single token. However, even if they contain multiple tokens, spelling them still would require those models to recognize the character-level composition of these tokens.

[6] K. Misra, J. Rayz, A. Ettinger, COMPS: Conceptual minimal pair sentences for testing robust property knowledge and its inheritance in pre-trained language models in Proc. EACL. pp. 2928–2949 (2023).

[7] P. Liang, et al., Holistic evaluation of language models. TMLR (2023).

[8] N. F. Liu, et al., Lost in the middle: How language models use long contexts. Transactions Assoc. for Comput. Linguist. **12**, 157–173 (2024).

[9] A. Webson, E. Pavlick, Do prompt-based models really understand the meaning of their prompts? in Proceedings of the 2022 Conference of the North American Chapter of the Association for Computational Linguistics: Human Language Technologies. (Association for Computational Linguistics, Seattle, United States), pp. 2300–2344 (2022).

[10] S. Min, et al., Rethinking the role of demonstrations: What makes in-context learning work? in Proceedings of the 2022 Conference on Empirical Methods in Natural Language Processing. (Association for Computational Linguistics, Abu Dhabi, United Arab Emirates), pp. 11048–11064 (2022).

[11] C. Raffel, et al., Exploring the limits of transfer learning with a unified text-to-text transformer. The J. Mach. Learn. Res. **21**, 5485–5551 (2020).

[12] S. J. Mielke, A. Szlam, E. Dinan, Y.-L. Boureau, Reducing conversational agents' overconfidence through linguistic calibration. Transactions Assoc. for Comput. Linguist. **10**, 857–872 (2022).

[13] S. Kadavath, et al., Language models (mostly) know what they know. arXiv [Preprint] (2022) `https://arxiv.org/abs/2207.05221` (accessed 28 August 2024).

[14] D. Marr, Vision. (W.H. Freeman), (1982).

[15] R. N. Shepard, Toward a universal law of generalization for psychological science. Science **237**, 1317–1323 (1987).

[16] J. R. Anderson, The Adaptive Character of Thought. (Erlbaum), (1990).

[17] E. Grant, C. Finn, S. Levine, T. Darrell, T. Griffiths, Recasting gradient-based meta-learning as hierarchical Bayes in International Conference on Learning Representations. (2018).

[18] C. Finn, P. Abbeel, S. Levine, Model-agnostic meta-learning for fast adaptation of deep networks in International Conference on Machine Learning. (PMLR), pp. 1126–1135 (2017).

[19] I. Tenney, D. Das, E. Pavlick, BERT rediscovers the classical NLP pipeline in Proceedings of the 57th Annual Meeting of the Association for Computational Linguistics. (Association for Computational Linguistics, Florence, Italy), pp. 4593–4601 (2019).

[20] J. Devlin, M.-W. Chang, K. Lee, K. Toutanova, BERT: Pre-training of deep bidirectional Transformers for language understanding in Proceedings of the 2019 Conference of the North American Chapter of the Association for Computational Linguistics: Human Language Technologies, Volume 1 (Long and Short Papers). (Association for Computational Linguistics, Minneapolis, Minnesota), pp. 4171–4186 (2019).

[21] M. Schrimpf, et al., The neural architecture of language: Integrative modeling converges on predictive processing. Proc. Natl. Acad. Sci. **118**, e2105646118 (2021).

[22] C. Caucheteux, J.-R. King, Brains and algorithms partially converge in natural language processing. Commun. Biol. **5**, 134 (2022).

[23] M. Shanahan, Talking about large language models. Commun. ACM (2024).

[24] A. Wang, et al., Can you tell me how to get past Sesame Street? Sentence-level pretraining beyond language modeling in Proceedings of the 57th Annual Meeting of the Association for Computational Linguistics. (Association for Computational Linguistics, Florence, Italy), pp. 4465–4476 (2019).

[25] C.-C. Lin, A. Jaech, X. Li, M. R. Gormley, J. Eisner, Limitations of autoregressive models and their alternatives in Proceedings of the 2021 Conference of the North American Chapter of the Association for Computational Linguistics: Human Language Technologies. (Association for Computational Linguistics, Online), pp. 5147–5173 (2021).

[26] R. T. McCoy, E. Grant, P. Smolensky, T. L. Griffiths, T. Linzen, Universal linguistic inductive biases via meta-learning. Proc. 42nd Annu. Conf. Cogn. Sci. Soc. pp. 737–743 (2020).

[27] S. Müller, N. Hollmann, S. P. Arango, J. Grabocka, F. Hutter, Transformers can do Bayesian inference in International Conference on Learning Representations. (2022).

[28] R. T. McCoy, T. L. Griffiths, Modeling rapid language learning by distilling Bayesian priors into artificial neural networks. arXiv [Preprint] (2023) `https://arxiv.org/abs/2305.14701` (accessed 28 August 2024).

[29] S. Abnar, M. Dehghani, W. Zuidema, Transferring inductive biases through knowledge distillation. arXiv [Preprint] (2020) `https://arxiv.org/abs/2006.00555` (accessed 28 August 2024).

[30] K. Mahowald, et al., Dissociating language and thought in large language models. TiCS (2024).

[31] S. Schuster, T. Linzen, When a sentence does not introduce a discourse entity, transformer-based models still sometimes refer to it in Proceedings of the 2022 Conference of the North American Chapter of the Association for Computational Linguistics: Human Language Technologies. (Association for Computational Linguistics, Seattle, United States), pp. 969–982 (2022).

[32] N. Kim, S. Schuster, Entity tracking in language models in Proceedings of the 61st Annual Meeting of the Association for Computational Linguistics (Volume 1: Long Papers). (Association for Computational Linguistics, Toronto, Canada), pp. 3835–3855 (2023).

[33] A. Ettinger, What BERT is not: Lessons from a new suite of psycholinguistic diagnostics for language models. Transactions Assoc. for Comput. Linguist. **8**, 34–48 (2020).

[34] E. Yiu, E. Kosoy, A. Gopnik, Imitation versus innovation: What children can do that large language and language-and-vision models cannot (yet)? arXiv [Preprint] (2023) `https://arxiv.org/abs/2305.07666` (accessed 28 August 2024).

[35] E. Kosoy, E. R. Reagan, L. Lai, A. Gopnik, D. K. Cobb, Comparing machines and children: Using developmental psychology experiments to assess the strengths and weaknesses of LaMDA responses. arXiv [Preprint] (2023) `https://arxiv.org/abs/2305.11243` (accessed 28 August 2024).

[36] M. Mitchell, D. C. Krakauer, The debate over understanding in AI's large language models. Proc. Natl. Acad. Sci. **120**, e2215907120 (2023).

[37] E. M. Bender, A. Koller, Climbing towards NLU: On meaning, form, and understanding in the age of data in Proceedings of the 58th Annual Meeting of the Association for Computational Linguistics. pp. 5185–5198 (2020).

[38] Y. Bisk, et al., Experience grounds language in Proceedings of the 2020 Conference on Empirical Methods in Natural Language Processing (EMNLP). (Association for Computational Linguistics, Online), pp. 8718–8735 (2020).

[39] D. C. Mollo, R. Millière, The vector grounding problem. arXiv [Preprint] (2023) `https://arxiv.org/abs/2304.01481` (accessed 28 August 2024).

[40] W. Merrill, A. Warstadt, T. Linzen, Entailment semantics can be extracted from an ideal language model in Proceedings of the 26th Conference on Computational Natural Language Learning (CoNLL). (Association for Computational Linguistics, Abu Dhabi, United Arab Emirates (Hybrid)), pp. 176–193 (2022).

[41] B. Z. Li, M. Nye, J. Andreas, Implicit representations of meaning in neural language models in Proceedings of the 59th Annual Meeting of the Association for Computational Linguistics and the 11th International Joint Conference on Natural Language Processing (Volume 1: Long Papers). (Association for Computational Linguistics, Online), pp. 1813–1827 (2021).

[42] S. Piantadosi, F. Hill, Meaning without reference in large language models in NeurIPS 2022 Workshop on Neuro Causal and Symbolic AI (nCSI). (2022).

[43] P. A. Ortega, et al., Shaking the foundations: Delusions in sequence models for interaction and control. arXiv [Preprint] (2021) https://arxiv.org/abs/2110.10819 (accessed 28 August 2024).

[44] G. K. Pullum, B. C. Scholz, Empirical assessment of stimulus poverty arguments. The Linguist. Rev. **19**, 9–50 (2002).

[45] T. Linzen, How can we accelerate progress towards human-like linguistic generalization? in Proceedings of the 58th Annual Meeting of the Association for Computational Linguistics. (Association for Computational Linguistics, Online), pp. 5210–5217 (2020).

[46] N. Kim, T. Linzen, P. Smolensky, Uncontrolled lexical exposure leads to overestimation of compositional generalization in pretrained models. arXiv [Preprint] (2022) https://arxiv.org/abs/2212.10769 (accessed 28 August 2024).

[47] D. Hupkes, et al., A taxonomy and review of generalization research in NLP. Nat. Mach. Intell. **5**, 1161–1174 (2023).

[48] A. Piktus, et al., The ROOTS search tool: Data transparency for LLMs in Proceedings of the 61st Annual Meeting of the Association for Computational Linguistics (Volume 3: System Demonstrations). (Association for Computational Linguistics, Toronto, Canada), pp. 304–314 (2023).

[49] P. A. Huebner, E. Sulem, F. Cynthia, D. Roth, BabyBERTa: Learning more grammar with small-scale child-directed language in Proceedings of the 25th Conference on Computational Natural Language Learning. pp. 624–646 (2021).

[50] L. Gao, et al., The Pile: An 800GB dataset of diverse text for language modeling. arXiv [Preprint] (2020) https://arxiv.org/abs/2101.00027 (accessed 28 August 2024).

[51] K. Lee, et al., Deduplicating training data makes language models better in Proceedings of the 60th Annual Meeting of the Association for Computational Linguistics (Volume 1: Long Papers). (Association for Computational Linguistics, Dublin, Ireland), pp. 8424–8445 (2022).

[52] X. Han, et al., Understanding in-context learning via supportive pretraining data in Proceedings of the 61st Annual Meeting of the Association for Computational Linguistics (Volume 1: Long Papers). (Association for Computational Linguistics, Toronto, Canada), pp. 12660–12673 (2023).

[53] J. Wei, D. Garrette, T. Linzen, E. Pavlick, Frequency effects on syntactic rule learning in Transformers in Proceedings of the 2021 Conference on Empirical Methods in Natural Language Processing. (Association for Computational Linguistics, Online and Punta Cana, Dominican Republic), pp. 932–948 (2021).

[54] Y. Razeghi, R. L. Logan IV, M. Gardner, S. Singh, Impact of pretraining term frequencies on few-shot numerical reasoning in Findings of the Association for Computational Linguistics: EMNLP 2022. pp. 840–854 (2022).

[55] X. Han, Y. Tsvetkov, ORCA: Interpreting prompted language models via locating supporting data evidence in the ocean of pretraining data. arXiv [Preprint] (2022) https://arxiv.org/abs/2205.12600 (accessed 28 August 2024).

[56] Y. Elazar, et al., Measuring causal effects of data statistics on language model's factual predictions. arXiv [Preprint] (2022) https://arxiv.org/abs/2207.14251 (accessed 28 August 2024).

[57] R. T. McCoy, P. Smolensky, T. Linzen, J. Gao, A. Celikyilmaz, How much do language models copy from their training data? evaluating linguistic novelty in text generation using RAVEN. Transactions Assoc. for Comput. Linguist. **11**, 652–670 (2023).

[58] E. Akyurek, et al., Towards tracing knowledge in language models back to the training data in Findings of the Association for Computational Linguistics: EMNLP 2022. (Association for Computational Linguistics, Abu Dhabi, United Arab Emirates), pp. 2429–2446 (2022).

[59] T. Blevins, L. Zettlemoyer, Language contamination helps explains the cross-lingual capabilities of English pretrained models in Proceedings of the 2022 Conference on Empirical Methods in Natural Language Processing. (Association for Computational Linguistics, Abu Dhabi, United Arab Emirates), pp. 3563–3574 (2022).

[60] S. Chan, et al., Data distributional properties drive emergent in-context learning in Transformers. Adv. Neural Inf. Process. Syst. **35**, 18878–18891 (2022).

[61] T. Bolukbasi, K.-W. Chang, J. Y. Zou, V. Saligrama, A. T. Kalai, Man is to computer programmer as woman is to homemaker? Debiasing word embeddings. Adv. Neural Inf. Process. Syst. **29** (2016).

[62] A. Caliskan, J. J. Bryson, A. Narayanan, Semantics derived automatically from language corpora contain human-like biases. Science **356**, 183–186 (2017).

[63] E. M. Bender, T. Gebru, A. McMillan-Major, S. Shmitchell, On the dangers of stochastic parrots: Can language models be too big? in Proceedings of the 2021 ACM Conference on Fairness, Accountability, and Transparency. pp. 610–623 (2021).

[64] S. Gehman, S. Gururangan, M. Sap, Y. Choi, N. A. Smith, RealToxicityPrompts: Evaluating neural toxic degeneration in language models in Findings of the Association for Computational Linguistics: EMNLP 2020. (Association for Computational Linguistics, Online), pp. 3356–3369 (2020).

[65] A. Deshpande, V. Murahari, T. Rajpurohit, A. Kalyan, K. Narasimhan, Toxicity in ChatGPT: Analyzing persona-assigned language models in Findings of the Association for Computational Linguistics: EMNLP 2023, eds. H. Bouamor, J. Pino, K. Bali. (Association for Computational Linguistics, Singapore), pp. 1236–1270 (2023).

[66] R. T. McCoy, E. Pavlick, T. Linzen, Right for the wrong reasons: Diagnosing syntactic heuristics in natural language inference in Proceedings of the 57th Annual Meeting of the Association for Computational Linguistics. (Association for Computational Linguistics, Florence, Italy), pp. 3428–3448 (2019).

[67] T. Niven, H.-Y. Kao, Probing neural network comprehension of natural language arguments in Proceedings of the 57th Annual Meeting of the Association for Computational Linguistics. (Association for Computational Linguistics, Florence, Italy), pp. 4658–4664 (2019).

[68] A. Rogers, Changing the world by changing the data in Proceedings of the 59th Annual Meeting of the Association for Computational Linguistics and the 11th International Joint Conference on Natural Language Processing (Volume 1: Long Papers). (Association for Computational Linguistics, Online), pp. 2182–2194 (2021).

[69] S. Merity, C. Xiong, J. Bradbury, R. Socher, Pointer sentinel mixture models in International Conference on Learning Representations. (2017).

[70] P. Smolensky, On the proper treatment of connectionism. Behav. Brain Sci. **11**, 1–23 (1988).

[71] J. L. McClelland, D. E. Rumelhart, G. E. Hinton, The Appeal of Parallel Distributed Processing. MIT Press. Camb. MA pp. 3–44 (1986).

[72] P. Smolensky, G. Legendre, Harmony optimization and the computational architecture of the mind/brain. The Harmon. Mind **1**, 1–61 (2006).

[73] P. Smolensky, R. T. McCoy, R. Fernandez, M. Goldrick, J. Gao, Neurocompositional computing: From the central paradox of cognition to a new generation of ai systems. AI Mag. **43**, 308–322 (2022).

[74] F. Lieder, T. L. Griffiths, Resource-rational analysis: Understanding human cognition as the optimal use of limited computational resources. Behav. brain sciences **43**, e1 (2020).

[75] J. L. Elman, Finding structure in time. Cogn. Sci. **14**, 179–211 (1990).

[76] S. Hochreiter, J. Schmidhuber, Long short-term memory. Neural Comput. **9**, 1735–1780 (1997).

[77] A. Vaswani, et al., Attention is all you need in Advances in Neural Information Processing Systems. pp. 5998–6008 (2017).

[78] I. Beltagy, M. E. Peters, A. Cohan, Longformer: The long-document Transformer. arXiv [Preprint] (2020) `https://arxiv.org/abs/2004.05150` (accessed 28 August 2024).

[79] J. Ainslie, et al., CoLT5: Faster long-range transformers with conditional computation in Proceedings of the 2023 Conference on Empirical Methods in Natural Language Processing, eds. H. Bouamor, J. Pino, K. Bali. (Association for Computational Linguistics, Singapore), pp. 5085–5100 (2023).

[80] S. Pinker, A. Prince, On language and connectionism: Analysis of a parallel distributed processing model of language acquisition. Cognition **28**, 73–193 (1988).

[81] D. E. Rumelhart, J. L. McClelland, On learning the past tenses of English verbs. Parallel Distributed Process. Explor. Microstruct. Cogn. (1986).

[82] R. Sennrich, B. Haddow, A. Birch, Neural machine translation of rare words with subword units in Proceedings of the 54th Annual Meeting of the Association for Computational Linguistics (Volume 1: Long Papers). (Association for Computational Linguistics), pp. 1715–1725 (2016).

[83] O. Ahia, et al., Do all languages cost the same? Tokenization in the era of commercial language models in Proceedings of the 2023 Conference on Empirical Methods in Natural Language Processing, eds. H. Bouamor, J. Pino, K. Bali. (Association for Computational Linguistics, Singapore), pp. 9904–9923 (2023).

[84] E. Salesky, D. Etter, M. Post, Robust open-vocabulary translation from visual text representations in Proceedings of the 2021 Conference on Empirical Methods in Natural Language Processing. (Association for Computational Linguistics, Online and Punta Cana, Dominican Republic), pp. 7235–7252 (2021).

[85] J. A. Fodor, Z. W. Pylyshyn, Connectionism and cognitive architecture: A critical analysis. Cognition **28**, 3–71 (1988).

[86] G. F. Marcus, Rethinking eliminative connectionism. Cogn. Psychol. **37**, 243–282 (1998).

[87] B. M. Lake, M. Baroni, Generalization without systematicity: On the compositional skills of sequence-to-sequence recurrent networks in International Conference on Machine Learning. pp. 2879–2888 (2018).

[88] D. Hupkes, V. Dankers, M. Mul, E. Bruni, Compositionality decomposed: How do neural networks generalise? J. Artif. Intell. Res. **67**, 757–795 (2020).

[89] N. Kim, T. Linzen, COGS: A compositional generalization challenge based on semantic interpretation in Proceedings of the 2020 Conference on Empirical Methods in Natural Language Processing (EMNLP). (Association for Computational Linguistics, Online), pp. 9087–9105 (2020).

[90] D. Zhou, et al., Least-to-most prompting enables complex reasoning in large language models in The Eleventh International Conference on Learning Representations. (2023).

[91] N. Dziri, et al., Faith and fate: Limits of transformers on compositionality in Thirty-seventh Conference on Neural Information Processing Systems. (2023).

[92] I. Schlag, et al., Enhancing the Transformer with explicit relational encoding for math problem solving in NeurIPS Workshop on Context and Composition in Biological and Artificial Neural Systems. (2019).

[93] T. W. Webb, I. Sinha, J. Cohen, Emergent symbols through binding in external memory in International Conference on Learning Representations. (2021).

[94] P. Smolensky, Tensor product variable binding and the representation of symbolic structures in connectionist systems. Artif. Intell. **46**, 159–216 (1990).

[95] E. Malach, Auto-regressive next-token predictors are universal learners. arXiv [Preprint] (2023) `https://arxiv.org/abs/2309.06979` (accessed 28 August 2024).

[96] A. Radford, et al., Language models are unsupervised multitask learners. OpenAI [Preprint] (2019) `https://openai.com/index/better-language-models` (accessed 28 August 2024).

[97] Y. LeCun, Y. Bengio, G. Hinton, Deep learning. Nature **521**, 436–444 (2015).

[98] J. Wei, et al., Chain-of-thought prompting elicits reasoning in large language models. Adv. Neural Inf. Process. Syst. **35**, 24824–24837 (2022).

[99] T. Kojima, S. S. Gu, M. Reid, Y. Matsuo, Y. Iwasawa, Large language models are zero-shot reasoners. Adv. Neural Inf. Process. Syst. **35**, 22199–22213 (2022).

[100] E. Davis, Mathematics, word problems, common sense, and artificial intelligence. arXiv [Preprint] (2023) `https://arxiv.org/abs/2301.09723` (accessed 28 August 2024).

[101] K. Arkoudas, GPT-4 can't reason. arXiv [Preprint] (2023) `https://arxiv.org/abs/2308.03762` (accessed 28 August 2024).

[102] V. I. Levenshtein, Binary codes capable of correcting deletions, insertions, and reversals in Soviet Physics Doklady. Vol. 10, pp. 707–710 (1966).

[103] I. R. McKenzie, et al., Inverse scaling: When bigger isn't better. arXiv [Preprint] (2023) `https://arxiv.org/abs/2306.09479` (accessed 28 August 2024).

[104] S. Bird, E. Loper, E. Klein, Natural Language Processing with Python. (O'Reilly Media Inc.), (2009).

[105] Y. Liu, et al., RoBERTa: A robustly optimized BERT pretraining approach. arXiv [Preprint] (2019) `https://arxiv.org/abs/1907.11692` (accessed 28 August 2024).

[106] R. Lebret, D. Grangier, M. Auli, Neural text generation from structured data with application to the biography domain in Proceedings of the 2016 Conference on Empirical Methods in Natural Language Processing. (Association for Computational Linguistics, Austin, Texas), pp. 1203–1213 (2016).

[107] R Core Team, R: A Language and Environment for Statistical Computing (R Foundation for Statistical Computing, Vienna, Austria), (2022).

[108] J. Friedman, T. Hastie, R. Tibshirani, Regularization paths for generalized linear models via coordinate descent. J. Stat. Softw. **33**, 1 (2010).

[109] J. K. Tay, B. Narasimhan, T. Hastie, Elastic net regularization paths for all generalized linear models. J. Stat. Softw. **106** (2023).

[110] H. Wickham, ggplot2: Elegant Graphics for Data Analysis. (Springer-Verlag New York), (2016).

[111] A. Gelman, A. Jakulin, M. G. Pittau, Y.-S. Su, A weakly informative default prior distribution for logistic and other regression models. The Annals Appl. Stat. **2**, 1360–1383 (2008).

[112] T. Hothorn, F. Bretz, P. Westfall, Simultaneous inference in general parametric models. Biom. J. **50**, 346–363 (2008).

[113] E. G. Wilcox, J. Gauthier, J. Hu, P. Qian, R. Levy, On the predictive power of neural language models for human real-time comprehension behavior. Proceeedings Cogn. Sci. Soc. (2020).

[114] Q. Lhoest, et al., Datasets: A community library for natural language processing in Proceedings of the 2021 Conference on Empirical Methods in Natural Language Processing: System Demonstrations. (Association for Computational Linguistics, Online and Punta Cana, Dominican Republic), pp. 175–184 (2021).

[115] B. Vaux, A. I. Nevins, Underdetermination in language games: Survey & analysis of Pig Latin dialects in 77th Annual Meeting of the LSA, Atlanta. pp. 2–5 (2003).
